# Comparative Transcriptome-Based Mining and Expression Profiling of Transcription Factors Related to Cold Tolerance in Peanut

**DOI:** 10.3390/ijms21061921

**Published:** 2020-03-11

**Authors:** Chunji Jiang, He Zhang, Jingyao Ren, Jiale Dong, Xinhua Zhao, Xiaoguang Wang, Jing Wang, Chao Zhong, Shuli Zhao, Xibo Liu, Shibo Gao, Haiqiu Yu

**Affiliations:** Peanut Research Institute, College of Agronomy, Shenyang Agricultural University, Shenyang 110161, China; jiangchunji2002@163.com (C.J.); ZhangHe_517@163.com (H.Z.); renjingyao163@163.com (J.R.); DJL0230@163.com (J.D.); xinhua_zhao@syau.edu.cn (X.Z.); wxglyj@163.com (X.W.); wj2105@163.com (J.W.); zhongchao1123@syau.edu.cn (C.Z.); zhaoshuli798@163.com (S.Z.); liuxibo@syau.edu.cn (X.L.); sgao@syau.edu.cn (S.G.)

**Keywords:** peanut, cold stress, transcription factor, comparative transcriptomic analysis, gene expression

## Abstract

Plants tolerate cold stress by regulating gene networks controlling cellular and physiological traits to modify growth and development. Transcription factor (TF)-directed regulation of transcription within these gene networks is key to eliciting appropriate responses. Identifying TFs related to cold tolerance contributes to cold-tolerant crop breeding. In this study, a comparative transcriptome analysis was carried out to investigate global gene expression of entire TFs in two peanut varieties with different cold-tolerant abilities. A total of 87 TF families including 2328 TF genes were identified. Among them, 445 TF genes were significantly differentially expressed in two peanut varieties under cold stress. The TF families represented by the largest numbers of differentially expressed members were bHLH (basic helix—loop—helix protein), C2H2 (Cys2/His2 zinc finger protein), ERF (ethylene-responsive factor), MYB (v-myb avian myeloblastosis viral oncogene homolog), NAC (NAM, ATAF1/2, CUC2) and WRKY TFs. Phylogenetic evolutionary analysis, temporal expression profiling, protein–protein interaction (PPI) network, and functional enrichment of differentially expressed TFs revealed the importance of plant hormone signal transduction and plant-pathogen interaction pathways and their possible mechanism in peanut cold tolerance. This study contributes to a better understanding of the complex mechanism of TFs in response to cold stress in peanut and provides valuable resources for the investigation of evolutionary history and biological functions of peanut TFs genes involved in cold tolerance.

## 1. Introduction

Peanut (*Arachis hypogaea L.*), one of the most important grain legumes as the source of edible oils and proteins, is cultivated in the tropical and subtropical regions of the world [1]. In recent years, with the increasing demand for peanuts, the planting areas are rapidly expanding in high-latitude areas such as Northeast China. However, extreme climate events such as low temperature caused by global climate change occur frequently and severely restrict peanut growth, development, productivity, and geographical distribution in temperate zones and high-elevation environments [2]. Understanding the specific mechanisms of peanut respond to and tolerate cold stress is the crucial first step to devise novel and rational strategies for improving peanut cold tolerance.

Most temperate or hardy plants have evolved specific strategies to survive low temperature, which involves a series of physiological, biochemical, molecular, and metabolic processes [3]. The multifaceted nature of the responses of the plant to cold stress and the intricate relationships between growth and response mechanisms, which in themselves are complex, dictate that cold tolerance is a highly complex, multigenic trait, as evidenced by a large number of quantitative trait locus (QTLs) that have been associated with cold tolerance [4,5]. Transcriptomic studies in model species under controlled conditions have indicated that the transcript abundances of hundreds of genes are altered under the imposition of cold stress [6,7]. These transcriptome changes are regulated by a complex network of transcription factors (TFs) and other regulatory proteins and RNAs. TFs act as transcriptional activators or repressors and play a central role in the regulation of development, metabolic processes, and biotic and abiotic stresses [8]. For example, members of APETALA2/ETHYLENE RESPONSIVE FACTOR (AP2/ERF) TF family have been suggested to positively regulate plant shoot branching, lateral root, and drought tolerance [9]. WRKY TFs are involved in the plant immune system mediated by jasmonic acid (JA) and salicylic acid (SA) to respond to attacks by pathogens [10,11]. Basic helix—loop—helix protein (bHLH) TF family is not only universally involved in plant growth and metabolism, including photomorphogenesis, light signal transduction, and secondary metabolism, but also plays an important role in plant response to stress [12].

Considering the importance of TFs in plant growth and development, there has been great progress in the past few years linking specific TF members with plant cold stress responses. A vast array of cold-responsive TFs has been identified in succession, which lays a foundation to illustrate the regulation network of plant cold tolerance. For example, the C-repeat binding factors/dehydration responsive element binding factor1 proteins (CBFs/DREB1s), which belong to the AP2/ERF TF family, are proved to be closely related to plant cold tolerance [13]. When plants are subject to cold stress, the *CBFs* can be rapidly induced in a very short period of time (about 15 min) [14]. *CBFs* are reported to play a central role in cold-responsive network by directly regulating the expression levels of *cold-responsive* (*COR*) genes, thus improving cold tolerance in many plant species [15,16,17]. Of course, there are different cis elements in CBF promoters, which can be recognized and combined by various TFs to regulate the expression of *CBFs* under cold stress. ICE1 (inducer of CBF expression 1) is an MYC-like bHLH transcriptional activator, which can bind to CBFs’ promoters and induce the expression of *CBF* genes under cold stress [18]. Ding et al. [19] reported that the overexpression of *ICE1* in Arabidopsis can induce the expression of *CBF1-3* and improved cold tolerance, while the knockout of *ICE1* significantly prevents the induction of *CBF* genes and reduces the ability to adapt to cold stress. CAMTA (calmodulin-binding transcription activator) is a group of transcriptional activators containing conserved calmodulin-binding sites and also can regulate the expression of *CBFs* [20]. In addition, different members of CAMTA family interact with specific *CBF* genes. Similar to *ICE1*, in Arabidopsis, *CAMTA1* and *CAMTA2* also can induce the upregulated expression of *CBF1-3* and other downstream genes under cold stress, while *CAMTA3* can activate the expression of *CBF2* by binding to the CM2 motif (CGCG-box) and then negatively regulate the cold tolerance [21]. Of course, there are also some members in various TF families that significantly suppress the activation of *CBFs* under cold stress, like MYB15, MYB30, and PIFs (Phytochrome-interacting factors) [22,23,24].

Given the evidence from model plants and multiple crops that TFs are central to plant cold tolerance, the cold tolerance mechanism of peanut based on TF regulation has not been reported. In recent years, several TF families have been identified from peanut genome with the completion of whole-genome sequencing, including bHLH, WRKY, bZIP (basic region/leucine zipper motif), etc. [25,26,27], while no information is available on peanut TFs in response to cold stress. In order to obtain a comprehensive insight to gene expression profiles of peanut TF families in response to cold stress, and identify the crucial candidate TF genes that may contribute to peanut cold tolerance, in this study, the comparative transcriptomic analyses of cold-tolerant peanut variety Nonghua5 (NH5) and cold-sensitive peanut variety Fuhua18 (FH18) were carried out. A set of TF genes in response to cold stress were identified in peanut and were classified into various families based on their homology and phylogenetic evolutionary relationships to that of model plants, including bHLH, C2H2, ERF, MYB, NAC, and WRKY superfamily. The differential expression and functional enrichment of these families clearly demonstrated their important roles in peanut cold tolerance. These results allow for the future targeting of relevant TFs that impact the peanut cold tolerance and for developing an understanding of the genetic networks and processes that affect growth under the imposition of stress and that impact stress acclimation and tolerance.

## 2. Results

### 2.1. Evaluation of Cold Tolerance of the Two Peanut Varieties

**Figure 1:**
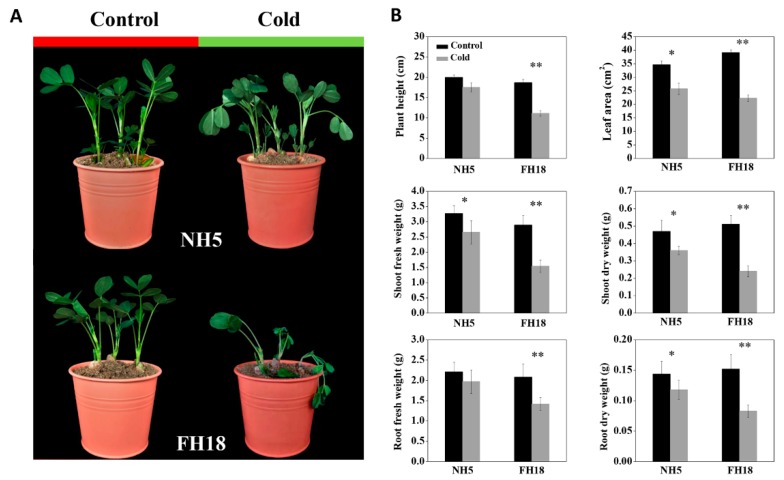
The changes in phenotype and morphology before and after cold treatment in Nonghua5 (NH5) and Fuhua18 (FH18). (**A**) Phenotypes of NH5 and FH18 under control and cold conditions, respectively. (**B**) Morphological indexes of NH5 and FH18 under control and cold conditions including plant height, leaf area, shoot fresh weight, shoot dry weight, root fresh weight, and root dry weight. Error bars represent the SD of the means (*n* = 3). * *p* < 0.05; ** *p* < 0.01.

The differences of peanut seedlings response to cold stress were evaluated based on the phenotypic changes and the changes of plant height (PH), total leaf area (TLA), shoot fresh weight (SFW), root fresh weight (RFW), shoot dry weight (SDW), and root dry weight (RDW). Highly significant differences in the phenotypic and morphological characteristics between NH5 and FH18 were observed after 6 °C cold stress for 7 days (Figure 1). Although the growth of both varieties was affected by cold stress, the magnitude of the decreases of FH18 was greater than those of NH5, especially for SFW, SDW, and RDW, which suggested that NH5 had better cold tolerance ability compared with the cold-sensitive variety FH18.

### 2.2. Physiological Responses of the Two Peanut Varieties to Cold Stress

**Figure 2:**
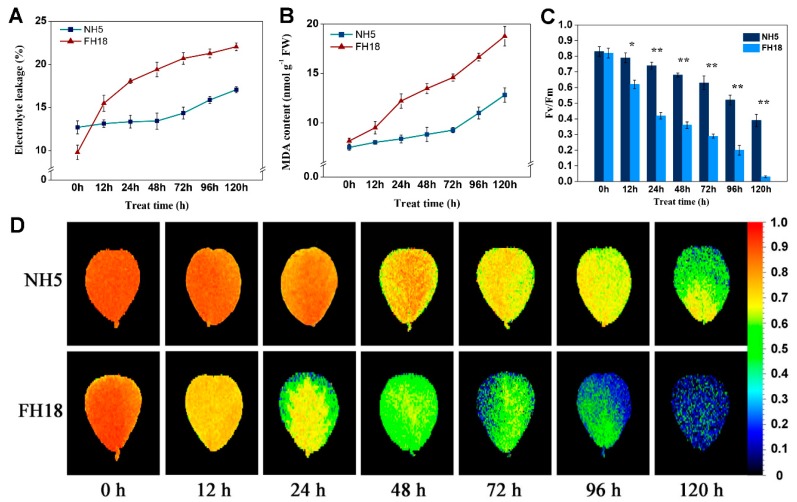
The physiological responses of the two peanut varieties to cold stress. (**A**) Electrolyte leakage of Nonghua 5 (NH5) and Fuhua18 (FH18) under cold stress. (**B**) Malondialdehyde of NH5 and FH18 under cold stress. (**C**) The maximum photochemical efficiency of PSII of NH5 and FH18 under cold stress. (**D**) The chlorophyll fluorescence in-situ imaging of NH5 and FH18 under cold stress. Error bars represent the SD of the means (*n* = 3). * *p* < 0.05; ** *p* < 0.01.

Electrolyte leakage (EL) and malondialdehyde (MDA) are important indicators for evaluating membrane stability. Under continuous cold stress, the EL and MDA content both showed an extremely increasing trend over the whole cold stress period in FH18 but were almost unchanged during the first 48 h of cold stress in NH5 (Figure 2A,B), which indicated the membrane stability of peanut seedlings was destroyed during the early stage of cold stress in FH18. The photosynthetic capacity of NH5 and FH18 seedlings after 0-h, 12-h, 24-h, 48-h, 72-h, 96-h, and 120-h cold stress were estimated by the maximum photochemical efficiency of PSII (Fv/Fm). As shown in Figure 2C,D, under cold stress, the Fv/Fm was significantly decreased in both peanut varieties, while the levels in NH5 were higher than those in FH18 at all time points. The differences between NH5 and FH18 reached an extremely significant level after 24 h of cold stress. Through the chlorophyll fluorescence in-situ imaging, it can be clearly observed that the edges of FH18 leaves began to turn blue after 24-h cold stress, which indicated the PSII was damaged during the early stage of cold stress in FH18.

### 2.3. Transcriptome Sequencing and de Novo Assembly

In order to identify genes involved in peanut cold tolerance during the early stage (first 24 h) of cold stress, a global transcriptomic analysis using the Illumina RNA-seq was carried out. The RNA was prepared from peanut leaf samples collected from 2-week old seedlings after 0 (control), 12, and 24 h of 6 °C cold treatment in NH5 and FH18, respectively. A total of 113.61 Gb clean data were generated for eighteen runs (three replicates for each treatment), and at least 5.82 Gb clean data was generated for every single run with more than 93.17% Q30 bases. Approximately 19.47–25.37 million clean reads were generated for each sample, and 85.83–95.36% of them were mapped to the reference genome (Table S1). A total of 64,999 predicted genes were obtained and analyzed to measure gene expression levels by the values of fragments per kilobase of transcript per million fragments mapped (FPKM). The quantitative real-time PCR (qRT-PCR) analyses were performed on ten differentially expressed genes and the reliability of RNA-seq data validated (Figure S1).

### 2.4. Identification of Transcription Factors from Peanut Transcriptome

The iTAK and HMMER analysis of peanut transcripts allowed the identification of 4193 non-redundant TFs, of which 2328 TF genes were effectively expressed (a gene whose FPKM value ≥1 was considered to be expressed in the RNA-Seq, and the detailed information was given in Table S2) and classified into 87 TF families (Table 1), among them, the MYB superfamily was the largest, including 92 (3.95%) MYB and 95 (4.08%) MYB-related members. The second-largest family was the bHLH family with 144 (6.19%) members, followed by the C2H2 (129, 5.54%), WRKY (95, 4.08%), C3H (86, 3.69%), ERF (79, 3.39%), bZIP (74, 3.18%), and NAC (73, 3.14%) families.

### 2.5. Differential Expression Analysis of the Transcription Factors under Cold Stress

Among all the 2328 TFs expressed in peanut transcriptome, a total of 1125 TFs were significantly differentially expressed under cold stress based on the false discovery rate (FDR) and the fold change (FC) of FPKM (FDR < 0.01 and FC ≥ 2) (Table S3). Of these, 694 TFs were commonly differentially expressed in both varieties, while 242 and 189 TFs were specifically expressed in NH5 and FH18, respectively (Table S4). Moreover, the differential expression level of 203 TFs of 694 common differentially expressed TFs was significantly higher in NH5 than in FH18 [FC(NH5/FH18) ≥ 2], which comprised the “cold-tolerant TF set” together with the 242 TFs differentially expressed only in NH5 and may play a central role in peanut cold tolerance (Table S4).

**Figure 3:**
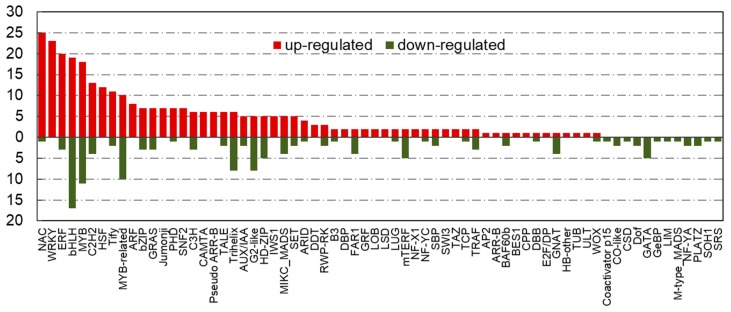
The number of differentially expressed transcriptional factors in “cold-tolerant transcription factor (TF) set” under cold stress. X-axis indicates various transcription factor families; Y-axis indicates the number of differentially expressed transcriptional factors. Red bars indicate upregulated; green bars indicate downregulated.

There were 69 TF families included in “cold-tolerant TF set”, which exhibited a significant difference in their expression levels in the two peanut cultivars under cold stress. The bHLH was the TF family with the most members (36 genes), MYB was the second-largest TF family with 29 gene members, followed by the NAC (26), ERF (23), WRKY (23), MYB-related (20), and C2H2 (17) (Figure 3). Of which, the most TF members in NAC (25), WRKY (23), ERF (20), MYB (18), and C2H2 (13) families were differentially upregulated expression in cold-tolerant peanut cultivar compared with cold-sensitive peanut cultivar, while the number of significantly upregulated and downregulated genes in bHLH and MYB-related TF families was almost equal. In addition, a large number of TFs in “cold-tolerant TF set” belonged to the extensively studied and well-characterized TF families viz. bHLH, C2H2, ERF, MYB, NAC, and WRKY, which have been investigated as key regulatory genes involved in plant abiotic stress responses and might play important roles in the regulation of peanut cold tolerance. Therefore, this study focused on the characterization of 154 TFs belonging to bHLH, C2H2, ERF, MYB, NAC, and WRKY families (Table S5).

### 2.6. Phylogenetic Analysis of Peanut Transcription Factor Families

**Figure 4:**
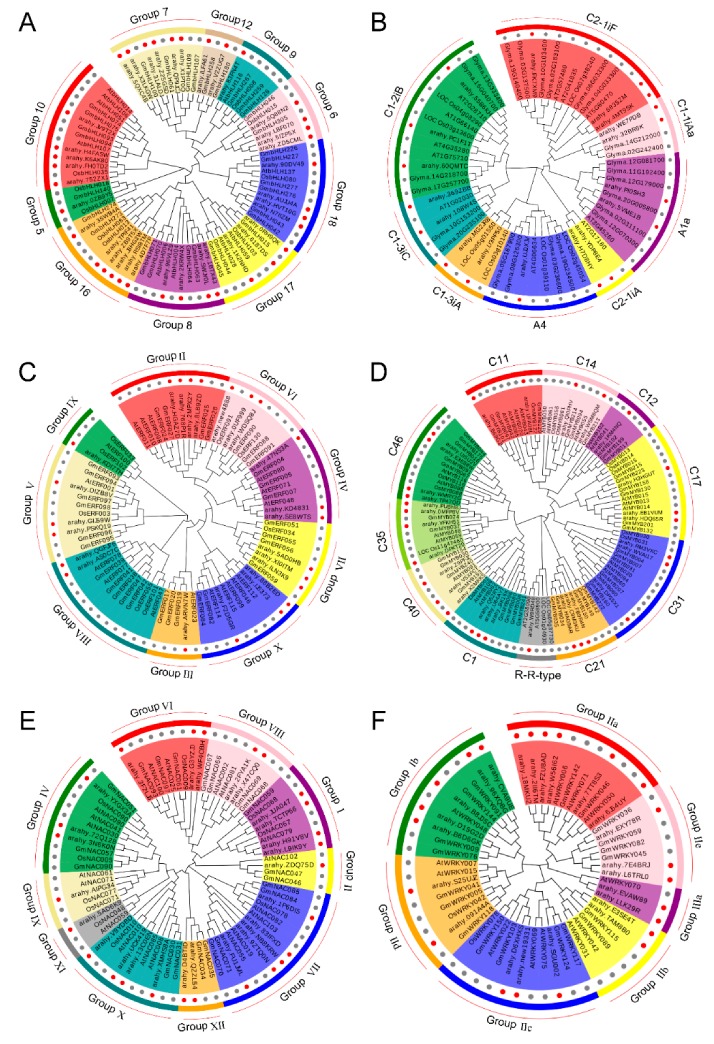
Phylogenetic relationships and subfamily designations of peanut transcription factor (TF) families along with model species *Arabidopsis thaliana*, *Oryza sativa*, and *Glycine max*. The colored branch indicates the different subfamilies. (**A**) bHLH (basic helix—loop—helix protein), (**B**) C2H2 (Cys2/His2 zinc finger protein), (**C**) ERF (ethylene-responsive factor), (**D**) MYB (v-myb avian myeloblastosis viral oncogene homolog), (**E**) NAC (NAM, ATAF1/2, CUC2), and (**F**) WRKY. Red dots indicate peanut TF genes.

To reveal the functions of uncharacterized TF genes in peanut, the unrooted neighbor-joining clustering phylogenetic trees were constructed based on the conserved domain alignment of bHLH, C2H2, ERF, MYB, NAC, and WRKY TF genes from peanut, Arabidopsis, rice, and soybean, respectively. As evident from Figure 4 (Figure S2), all the 154 peanut TF proteins could be efficiently grouped into their respective families with subgroups based on their phylogenetic affinity. A total of 21, 22, and 24 groups have been described for the bHLH family in Arabidopsis, rice, and soybean, respectively [28,29]. In our study, the 36 peanut bHLH TFs were grouped into 10 groups, the Group 7, Group 16, and Group 10 formed the major groups with 6, 6, and 5 members, respectively (Figure 4A). Of the 8 main C2H2 groups (A1, A2, A3, A4, B, C1, C2, and C3) identified in Arabidopsis, the phylogenetic tree separated the 17 peanut C2H2 into Group A1a, A4, C1-1iAa, C1-2iB, C1-3iA, C1-3iC, C2-1iA, and C2-1iF (Figure 4B), suggesting that most members belonged to Group C1 C2H2 [30]. Previous studies have divided the ERF gene family of Arabidopsis, rice, and soybean into 12, 15, and 12 subfamilies, respectively [31,32]. Here, 23 peanut ERF genes were assigned to 9 subfamilies, except for fewer members in the Group III and IX, most members were evenly distributed in respective subgroups, suggesting that most of ERF groups might have a vital function in peanut cold tolerance (Figure 4C). The MYB superfamily has been extensively studied in well-characterized model species [33,34]. In this study, the phylogram clustered the 29 peanut MYBs into 11 groups (Figure 4D). The analysis revealed that almost all the cold-tolerant MYBs in peanut belonged to the R2R3 group with only one member belonging to R-R-type group having a single MYB domain. Moreover, the 26 peanut NAC genes could be assigned to 10 groups (Figure 4E), together with reference NACs used for the analysis [35,36]. Group VII, I, and X formed the major group with 13 peanut NAC genes totally, whereas group II, XI, and IX contained only one number, respectively, suggesting diversified NAC subgroups may take effect in peanut under cold stress. As illustrated in Figure 4F, of the 3 main WRKY groups identified in Arabidopsis, rice, and soybean [37,38], the 23 peanut WRKYs were distributed into 7 groups (I b, II a-e, and III a) with Group II forming the major clade with 16 members.

### 2.7. Analysis of Protein Characterization and Conversed Motifs of Peanut Transcription Factor Families

Protein primary structure, the amino acid sequence, determines the protein function. Many functional features of proteins can be obtained directly from the analysis of their sequence characteristics, including amino acid composition, molecular mass, and isoelectric point, etc. The ExPASy is an expert protein analysis system and can calculate various physical and chemical properties of proteins. Amino acid sequence analysis of peanut TFs showed a range of variation in their amino acid length (107–1666), molecular weight (12.06–181.06 kDa), and isoelectric point (4.54–9.92). The average value of the aliphatic index was 67.25. The vast majority of TFs (more than 91.56%) were predicted as unstable proteins with an instability index value of more than 40, which envisaged that these variations could be attributed to the presence of putative novel variants and needed to be validated by further research. Subcellular location analysis showed that almost 86.36% peanut TF proteins were located in the nucleus and only a few TFs were predicted in the cytoplasm, chloroplast, mitochondria, and plasma membrane (Table S5).

In order to investigate the structural diversity and conservatism of motif compositions in peanut TFs, a total of 10 conserved motifs for each family were captured by MEME software (Figure S3), and the motif sequences were shown in Figure S4. For the bHLH family, Motif 2 and Motif 3 were presented in almost all the sequences irrespective of the subgroups and were confirmed to be the highly conserved helix regions. The Motif 1-Motif 4 in MYB TF sequences were the most conserved regions, which contained conversed W loci and were considered as the R2/R3 domain. In general, there were always several conserved motifs existed in each TF family, and the motif composition of TF members in the same group was almost similar, which indicated these motifs may be crucial for TF functional grouping. However, the motif structures were diverse between different subgroups even in the same TF family. For example, in C2H2 TF family, some sequences contained conserved Motif 2, Motif 3, and Motif 4, but others were comprised only by Motif 8. This result reflected the complex function nature of TF genes in peanut. The motif distribution indicated that the genes containing the same motifs were likely produced via gene expansion within the same groups.

### 2.8. Temporal Expression Profiles and Gene Ontology Enrichment

**Figure 5:**
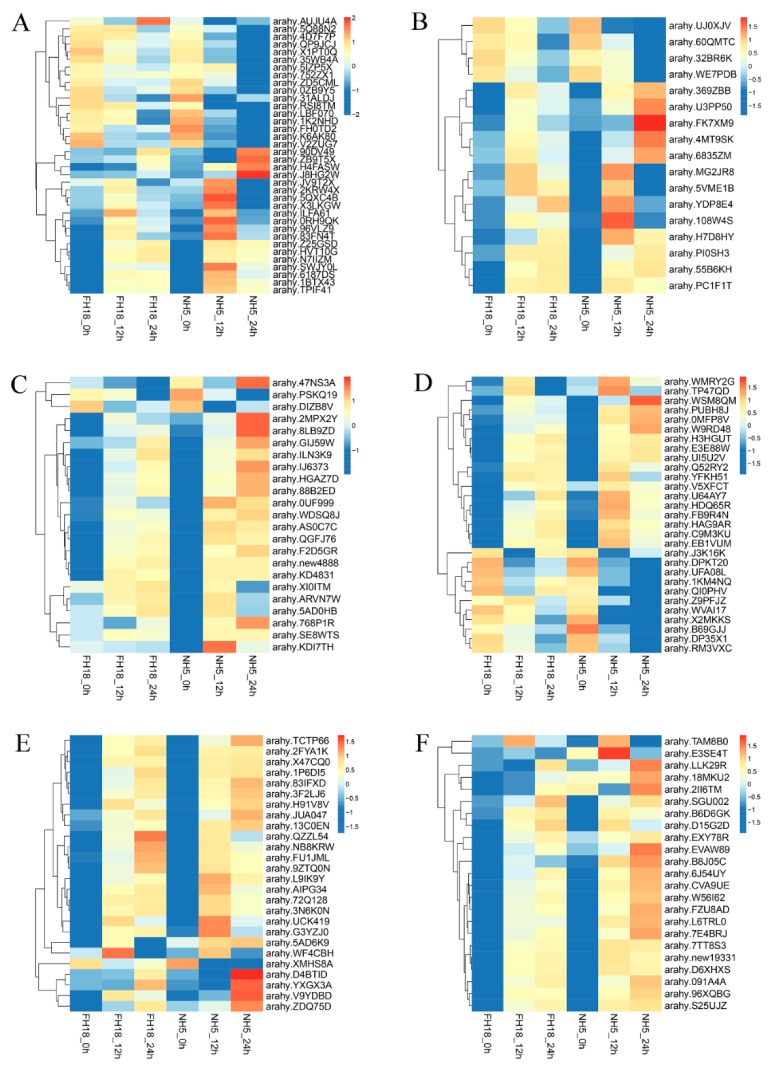
Heat map representing temporal expression profiles of peanut transcription factors under cold stress. The samples were collected at 0, 12, and 24 h cold stress. (**A**) bHLH (basic helix—loop—helix protein), (**B**) C2H2 (Cys2/His2 zinc finger protein), (**C**) ERF (ethylene-responsive factor), (**D**) MYB (v-myb avian myeloblastosis viral oncogene homolog), (**E**) NAC (NAM, ATAF1/2, CUC2), and (**F**) WRKY.

The activation of the cold-responsive transcriptome is a complex and multidimensional process. Analyzing temporal gene expression patterns may provide important clues for gene functions. Here, we analyzed the temporal expression profiles of six peanut TF families during the early stage of cold stress. A total of 154 TF genes holding a significant expression at least in one time frame as compared to control were shortlisted (Figure 5). Modulation varied in different time frames, and some TFs showed a steady upregulation or downregulation, for example, most TF genes in ERF, NAC, and WRKY families showed continuous upregulation in NH5 under cold stress. While others depicted variable expression profiles, *arahy.5QXC4B* (*bHLH25*), *arahy.0RH9QK* (*MYC2*), *arahy.83FN4T* (*bHLH13*), *arahy.108W4S* (*MGP*), *arahy.KDI7TH* (*AP2.7*), and *arahy.E3SE4T* (*WRKY70*) showed most significant upregulation only at 12 h cold stress, and *arahy.J8HG2W* (*ICE1*), *arahy.FK7XM9* (*SAP11*), *arahy.2MPX2Y*/*arahy.8LB9ZD* (*DREB2C*), *arahy.WSM8QM* (*MYB55*), *arahy.D4BTID* (*NAC82*), and *arahy.EVAW89* (*WRKY53*) depicted most significant upregulation only at 24 h cold stress in NH5. The differential expression levels of genes of the same TF family demonstrated that the different members have distinct mechanisms in response to cold stress and appeared to exhibit differential biological functions.

**Figure 6:**
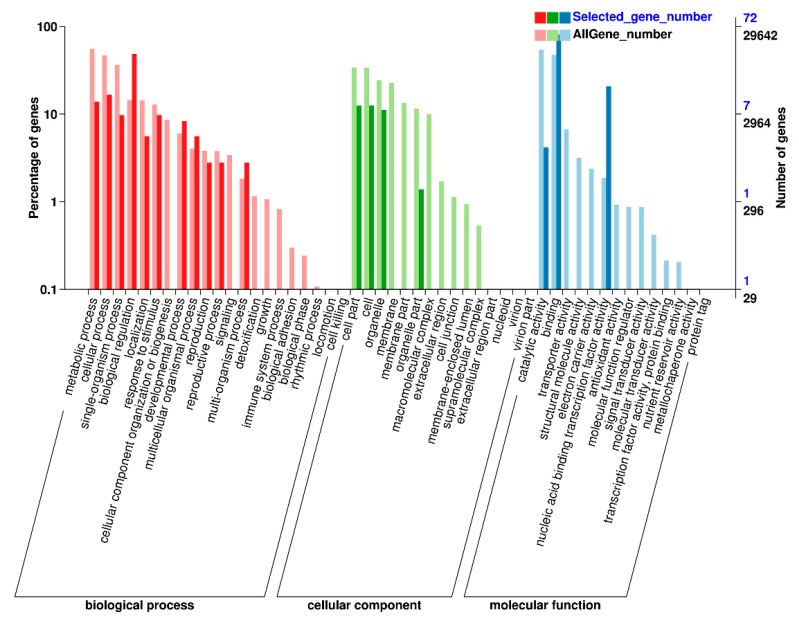
Gene ontology enrichment analysis of 154 differentially expressed transcription factors in peanut under cold stress.

The Gene ontology (GO) analysis using Blast2GO software distributed these 154 TFs into three main functional biological categories: biological process, cellular component, and molecular function (Figure 6). Of all the GO terms, the biological process had the most abundant differentially expressed TFs enriched, and a predominant portion of TFs was found to be involved in biological regulation, cellular process, and metabolic process. In cellular component category, cell part, cell, organelle, and organelle part were enriched. The molecular function analysis revealed that most of the 154 TFs corresponded to binding, nucleic acid binding transcription factor activity, and catalytic activity.

### 2.9. Protein Interactions and Module Analysis

**Figure 7:**
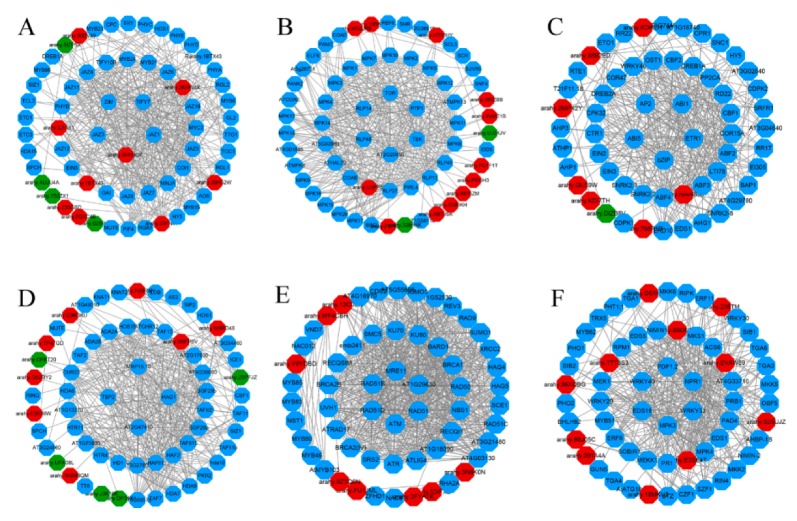
Protein–protein interaction (PPI) Network analysis of peanut transcription factors (TFs) in Clusters of Orthologous Group (COG) mode using STRING 11.0 database showing all connections of TF genes with a confidence score>0.4. Red nodes, upregulated TF genes; green nodes, downregulated TF genes; blue nodes, predicted genes interacting with peanut TFs. (**A**) bHLH (basic helix—loop—helix protein), (**B**) C2H2 (Cys2/His2 zinc finger protein), (**C**) ERF (ethylene-responsive factor), (**D**) MYB (v-myb avian myeloblastosis viral oncogene homolog), (**E**) NAC (NAM, ATAF1/2, CUC2), and (**F**) WRKY.

Based on the STRING database, the interaction networks of six peanut TF families were constructed (Figure 7), which highlighted several protein functional groups interacting with each other and demonstrated their role in diverse biological processes to adapt to cold stress. A total of 13, 14, 8, 13, 8, and 11 peanut TFs in bHLH, C2H2, ERF, MYB, NAC, and WRKY were interacted, respectively (Table S6). Of which, *arahy.0RH9QK* (*MYC2*), *arahy.2KRW4X* (*MYC4*), *arahy.ILFA61* (*PIF3*), *arahy.U3PP50* (*HAL3A*), *arahy.new4888* (*ERF13*), *arahy.E3SE4T* (*WRKY70*), *arahy.EVAW89* (*WRKY53*), and *arahy.B6D6GK* (*WRKY22*) had the most nodes and protein pairs, and all of them were upregulated, suggesting their essential role in peanut cold tolerance. Pathway analysis of six protein-protein interaction (PPI) networks showed that plant hormone signal transduction (49 genes), plant-pathogen interaction (26 genes), and plant mitogen-activated protein kinase (MAPK) signaling pathway (14 genes) were most significantly enriched (Table S6).

Furthermore, several hub genes with a high degree of connectivity of each TF family were selected (Table S6). In order to understand the biological function of these hub genes under cold stress, the Kyoto Encyclopedia of Genes and Genomes (KEGG) enrichment analysis was carried out. The hub genes of bHLH TF family mainly involved in JA signaling pathway. Most hub genes of C2H2 and ERF PPI networks participated in abscisic acid (ABA) signaling pathway. Furthermore, hub genes of WRKY gene family mostly enriched in JA signaling, SA signaling, MAPK signaling, and plant-pathogen interaction pathways.

## 3. Discussion

Cold stress seriously affects the growth, development, yield, and quality of crops, which has become the key limiting factor for the peanut planting in high altitude and severe cold region [39]. Therefore, it is considered as the critical importance for peanut extension to elucidate the specific mechanism and promote the genetic improvement of cold tolerance in peanut. The TFs are essential regulators involved in various biological processes. Numerous studies have identified TFs and other regulatory molecules that regulate transcription and signal transduction through stress-responsive genetic networks as prime targets for understanding and improving plant cold tolerance [40,41]. However, research on the mechanism of peanut cold tolerance is still at the morphological and physiological level, and the signal transduction of cold-tolerant peanut is poorly studied. In this study, a comparative transcriptomic analysis of cold-tolerant peanut cultivar and cold-sensitive peanut cultivar was carried out and some key candidate TFs involved in peanut cold tolerance were identified. To the best of our knowledge, this is the first comprehensive analysis of expression profiles of peanut TFs in response to cold stress at the whole genome level, which may provide a reference for studying the molecular mechanism of peanut cold tolerance.

TFs exist in the upstream of plant growth and development regulatory network, which can simultaneously regulate the expression of multiple genes. In molecular breeding, improving or enhancing the regulatory capacity of one crucial transcription factor can promote the function of several genes, which can obtain a comprehensive improvement effect compared with changing the expression of a single gene. Although some TFs responsive to cold stress have been identified and analyzed in previous reports [42,43], there were few studies on the cold-induced changes of expression profiles of the entire plant TF families based on transcriptome scale. Here, a total of 2328 TF genes were identified in the transcriptome and classified into 87 TF families. Of these, 445 TFs (19.12% of all 2328 detected TF genes) were differentially expressed in the two peanut varieties under cold stress. A large number of TFs that were differentially expressed was not surprising as cold stress affect an array of plant developmental and biochemical and physiological processes [44], which also proved the vital position of TFs in peanut cold tolerance.

The TF families represented by the largest numbers of differentially expressed members were the bHLH (36), MYB (29), NAC (26), ERF (23), WRKY (23), MYB-related (20), and C2H2 (17) TFs, which are also some of the largest TF families predicted in the peanut genome. The majority of NAC, WRKY, ERF, bHLH, MYB, and C2H2 members were significantly upregulated in cold-tolerant variety, whereas MYB-related family members were downregulated. Similar to this study, a comparative transcriptomic analysis of seedlings in two indica rice genotypes under chilling stress identified 1583 TFs, of which, WRKY, bZIP, NAC, ERF, MYB/MYB-related, and bHLH families had the most differentially expressed gene members [45]. These TF genes in respective families were divided into diverse subgroups based on their phylogenetic affinity and possessed specific motif structures, indicating they may perform their respective biological functions under cold stress. Therefore, these differentially expressed TFs identified in plants that respond to cold stress are potentially cold-tolerant genes and may serve as an important genetic resource for cold-tolerance crop breeding.

In addition, individual TFs within these families have previously been shown to have important roles in regulating plant cold tolerance. ICE1, belonging to bHLH TF family, is considered as a conserved transcription activator of CBF expression under cold stress [46]. Here, *ICE1* (ortholog of *arahy.J8HG2W*) was continuously differentially expressed in NH5 under cold stress, indicating that ICE1 also played an important role in peanut cold tolerance. In ERF TF family, two *ERFs arahy.2MPX2Y* and *arahy.8LB9ZD* (orthologs of Arabidopsis ERF gene *DREB2C*) were continuously upregulated in NH5 under cold stress. DREB2C could bind C-repeat/dehydration response element in vitro and possesses transcriptional activity [47]. Lee et al. [48] reported that DREB2C physically interacts with ABF2, a bZIP protein that regulates ABA-responsive gene expression, and *DREB2C* overexpression lines displayed increased ABA sensitivity. Therefore, *DREB2C* TF gene may regulate peanut cold tolerance by mediating ABA signaling pathway. WRKY is one of the largest families of TFs in plants and is divided into three groups, and Group II formed the major clade with 16 members in this study. Zhang et al. [49] suggested that overexpression of *VaWRKY12*, whose nuclear translocation increased under low temperature, enhanced the cold tolerance of Arabidopsis and grapevine calli and significantly increased the expression of antioxidant-related genes. Under cold stress, *VaWRKY33* can be regulated by ethylene response factor *VaERF092* through binding to its promoter GCC-box, leading to enhanced cold stress tolerance [50]. In our study, *arahy.E3SE4T* and *arahy.EVAW89*, orthologous to *A. thaliana WRKY70* and *WRKY53*, were significantly upregulated in NH5 under cold stress. While the information about *WRKY70* and *WRKY53* involve in regulation of plant cold tolerance has not been reported. The PPI network analysis indicated that *WRKY70* and *WRKY53* both can interact with hub genes *mitogen-activated protein kinase 3* (*MPK3*), *WRKY33,* and *WRKY40*, which were all involved in plant defense responses [51,52,53]. Therefore, *WRKY70* and *WRKY53* may regulate peanut cold tolerance through plant–pathogen interaction pathway.

KEGG pathway analysis of six PPI networks showed that plant hormone signal transduction (49 genes), plant-pathogen interaction (26 genes), and plant MAPK signaling pathway (14 genes) were most significantly enriched under cold stress. In fact, plants have evolved sophisticated strategies in which hormone and cold signaling pathways are coordinated to better adapt to cold stress. ABA regulates a number of physiological and developmental processes in plants and it is also considered as a major stress factor since abiotic stresses upregulate its synthesis [54]. Many TFs from the bZIP, ERF, MYC, MYB, and NAC protein families are known to function in an ABA-dependent manner under cold stress [55]. Here, several repressors (ABI1, ABI5, and PP2C) of ABA signaling pathway were hub genes and interacted with C2H2 and ERF TF members, which may play an important role in peanut cold tolerance. Recent studies have revealed unexpected roles for JAs as positive regulators of cold tolerance. Firstly, exposure to cold rapidly elevates endogenous JA levels by inducing JA biosynthesis genes such as *LOX1*, *AOS1*, *AOC1*, and *JAR1* in Arabidopsis. JA as a lipid-derived plant hormone can positively regulate CBF signaling by mediating the interaction between JAZ1/4 protein and ICE1/2, thus modulating the transcriptional activity of ICE proteins and *CBF1–3* gene expression [56]. Here, *arahy.J8HG2W*, an ortholog of *ICE1,* showed significant upregulation in NH5 under cold stress and can interact with JAZ1 and JAZ3 to mediate peanut cold tolerance. Besides, two MYC-type TFs in bHLH family *arahy.0RH9QK* and *arahy.2KRW4X* (orthologs of Arabidopsis *MYC2* and *MYC4*) were also upregulated. Min et al. [57] reported *SlMYC2* might be involved in MeJA-induced chilling tolerance, possibly by ameliorating the antioxidant enzyme system of tomato fruit and increasing proline and lycopene levels. These results indicated *ICE1* (*arahy.J8HG2W*), *MYC2* (*arahy.0RH9QK*), and *MYC4* (*arahy.2KRW4X*) in bHLH family played an important role in peanut cold tolerance, which were positively regulated by JA signaling pathway. Cold stress alters endogenous ethylene (ET) levels in many plant species. Accordingly, associations between enhanced ET levels and cold tolerance were observed [58]. However, the role of ET in cold tolerance is somewhat controversial in plants. EIN3 (ethylene-insensitive 3) is a key TF working in the ET signaling pathway. Shi et al. [59] suggested that EIN3 can combine with the EBS motifs existed in CBFs’ promoters and interfere with the expression of *CBFs* under cold stress, this result proved EIN3 can negatively regulate cold tolerance in Arabidopsis through ethylene signaling pathway. Conformably, EBF1 and EBF2 are two F-box proteins in ethylene signaling pathway, which can mediate the degradation of EIN3 and PIF3 (phytochrome-interacting factor) through a ubiquitin/proteasome pathway thus to induce the upregulation of *CBFs* [60]. The above results provide enough evidence for the standpoint that ethylene negatively regulates cold tolerance. However, by contrast, there are also several studies that suggested ethylene positively regulates plant cold tolerance. ERF2 is the crucial TF in ethylene biosynthesis and ethylene signaling pathway. Zhang et al. [61] proved the overexpression of tomato *ERF2* gene can enhance plant cold tolerance. In this study, *ERF13* and *ERF98* (orthologs of *arahy.new4888* and *arahy.GIJ59W*) were significantly upregulated in NH5, which indicated that ethylene may have a positive role in regulating peanut cold tolerance.

In summary, cold tolerance of plants was a complex process, involving the regulation of a set of TF families and various genes. This study presents a comprehensive identification, characterization, and temporal expression profiling of the entire TF families in peanut, which reveal the obvious differences in the gene expression of TFs between cold-tolerant peanut variety NH5 and cold-sensitive peanut variety FH18 under cold stress. Further phylogenetic analysis, PPI network analysis, and functional enrichment of differentially expressed members in bHLH, C2H2, ERF, MYB, NAC, and WRKY identify the crucial candidate TF genes and reveal the importance of plant hormone signal transduction and plant-pathogen interaction pathways in peanut cold tolerance. Our study may provide a foundation for in-depth analysis of molecular mechanism of TFs involved in peanut cold tolerance. These results provide a reference for identifying central TFs related to crop cold tolerance and enhancing the ability of crops to adapt to cold stress at the molecular level by means of genetic engineering.

## 4. Materials and Methods

### 4.1. Plant Materials, Growth Conditions, and Treatments

Two peanut cultivars with contrasting responses to low temperature were used in this study: the cold-tolerant cultivar Nonghua5 (NH5) and the cold-sensitive cultivar Fuhua18 (FH18). NH5 is the core parent used for breeding cold-tolerant peanut germplasm in Northeast China. FH18 is a representative peanut variety planted in large areas of northeast China. The peanut seedlings cultivation and all experiments were carried out at the Peanut Research Institute, Shenyang Agricultural University (Shenyang, China).

Plump seeds were surface sterilized with 3% sodium hypochlorite for 10 min, washed with distilled water five times, soaked in distilled water for 12 h, then placed in Petri dishes with moistened filter papers, and germinated in the dark at 28 °C in a growth chamber. After 2 days, the germinated seeds were sown in round plastic pots filled with clean river sand and half-strength Hoagland’s solution and subsequently transferred to a climate chamber under a 16 h light (28 °C)/8 h dark (23 °C) cycle, a photosynthetic photon flux density of 700 µmol m^−2^ s^−1^, and a relative humidity of 70%.

Two-week-old seedlings were grouped into two groups. One group was transferred to another climate chamber maintained under 6 °C, a 16 h/8 h cycle (light/dark), a photosynthetic photon flux density of 700 µmol m^−2^ s^−1^, and relative humidity of 70%. The other group with the same developmental progression was used as a control and maintained under normal conditions. The second leaves from the treatment and control were collected at 0 h, 12 h, 24 h, 48 h, 72 h, 96 h, and 120 h, respectively, frozen in liquid nitrogen, and stored at −80 °C for measurements. All experiments were repeated at least three times.

### 4.2. Morphological Parameters

The cold tolerance of the two peanut cultivars were evaluated by observing the phenotypes and measuring the changes of plant height (PH), total leaf area (TLA), shoot (including leaves) fresh weight (SFW), root fresh weight (RFW), shoot dry weight (SDW), and root dry weight (RDW) after 7 days of 6 °C cold treatment. Fifteen seedlings from each of the three treatments (five seedlings per replicate) were selected, and the PH per plant was recorded. The TLA per plant was measured using an electronic area meter (Li-Cor3000, Li-Cor, Lincoln, NE, USA). Then, shoots and roots were separated, and the SFW and RFW were recorded. All plant tissues were dried at 105 °C for 15 min and at 80 °C for 72 h, and then the SDW and RDW were recorded.

### 4.3. Physiological Characteristics

Electrolyte leakage (EL) was determined using an electrical conductivity meter (DDSJ-308F, Shanghai, China). Small circular leaf samples were obtained by a 7-mm-diameter hole punch. Then, these circles were rinsed three times with deionized water and dried with filter papers. Twenty circles per replicate were put into a test tube with 20 mL deionized water. The initial electrical conductivity (EC1) of the washing solutions was measured after the samples were incubated at 25 °C for 3 h. Then, the tubes were placed in a boiling water bath for 30 min, and the electrical conduction (EC2) was measured again after the solution cooled to room temperature. The EL was calculated using the formula EL = EC1/EC2 × 100.

Fresh leaves (0.5 g) were homogenized in 5 mL of 5% (*m/v*) trichloroacetic acid (TCA) and centrifuged at 12,000× *g* and 4 °C for 10 min. The supernatant (2 mL) was mixed with an equal volume of 0.5% thiobarbituric acid (TBA). The mixture was placed in boiling water for 15 min and then instantly cooled in an ice bath and centrifuged at 10,000× *g* and 4 °C for 10 min. The absorbance of the supernatant was measured at 450, 532, and 600 nm. The MDA content was calculated as follows, where Vt and Vs are the total volume of the extract solution and the volume of the extract solution contained in the reaction mixture, respectively, and m is the mass of the sample: MDA (nmol g^−1^ FW) = [6.45 × (A532 − A600) − 0.56 × A450] × Vt/(Vs × m).

The fluorescence parameter Fv/Fm and chlorophyll fluorescence images of the second leaves under cold and normal conditions were analyzed after 30 min of dark adaptation using a chlorophyll fluorescence imaging system FluorCam 7 (Photon Systems Instruments, Brno, Czech Republic).

### 4.4. Total RNA Extraction, Library Construction, and Sequence Analysis

Total RNA from the second leaves of peanut seedlings subjected to 0 (control), 12, and 24 h of cold stress was extracted using TRIzol reagent (Carlsbad, CA, USA). Then, RNA concentration was tested by a micro-spectrophotometer (OD_260/280_), and the RNA integrity was tested using Agilent Bioanalyzer 2100 system. After the RNA quality of each sample meets the standards of library construction, a total amount of 20 μg RNA per sample was used for library construction. mRNA was purified from total RNA using poly-T oligo-attached magnetic beads. Fragmentation was carried out using divalent cations under elevated temperature in NEBNext First Strand Synthesis Reaction Buffer. First-strand cDNA was synthesized using random hexamer primer and M-MuLV Reverse Transcriptase. Second-strand cDNA synthesis was subsequently performed using DNA Polymerase I and RNase H. In order to select cDNA fragments of preferentially 240 bp in length, the library fragments were purified with AMPure XP system (Beckman Coulter, Beverly, MA, USA). Then, 3 μL USER Enzyme (NEB, Ipswich, MA, USA) was used with size-selected, adaptor-ligated cDNA at 37 °C for 15 min followed by 5 min at 95 °C before PCR. Then, PCR was performed with Phusion High-Fidelity DNA polymerase, Universal PCR primers, and Index (X) Primer. At last, PCR products were purified (AMPure XP system) and library quality was assessed on the Agilent Bioanalyzer 2100 system. The library preparations were sequenced on an Illumina platform and paired-end reads were generated. Raw data (raw reads) of fastq format were firstly processed through in-house perl scripts. The adaptor sequences and low-quality sequence reads were removed from the data sets. Raw sequences were transformed into clean reads after data processing. Finally, these clean reads were mapped to the peanut reference genome version Tifrunner.gnm1. ann1. CCJH, the RNA-Seq data has been submitted to the online SRA (Sequence Reads Archive) database with the accession number PRJNA602777.

### 4.5. Quantitative RT-PCR

Quantitative real-time RT-PCR (qRT-PCR) was performed to validate the RNA-seq results. Ten differentially expressed genes (DEGs) were randomly selected for qRT-PCR. All primers were designed using Primer Premier v5.0 (Premier, San Francisco, CA, USA) (Table S7). The peanut *actin* gene (GenBank accession NC_037620) served as internal control. The SYBR Premix Ex Taq™ (TaKaRa, Inc., Dalian, China) was used for real-time quantification. According to the manufacturer’s instructions, PCR mixtures (10 μL) contained 1.0 μL cDNA, 0.3 μL each primer, 3.4 μL ddH_2_O, and 5.0 μL SYBR^®^ Green Master Mix. The amplification conditions were as follows: 60 s denaturation at 95 °C, followed by 40 cycles of 95 °C for 15 s, 55 °C for 30 s, and 72 °C for 60 s. Three biological replicates were used per experiment.

### 4.6. Identification of Peanut Transcription Factors

In order to identify the peanut TFs completely, the homologous search of RNA-Seq database against the plant TF database (http://planttfdb.cbi.pku.edu.cn/) (accessed on 2 December 2019) was carried out using the software iTAK v 1.7a [62]. Then, the conserved domains of each TF sequence were identified based on the Hidden Markov Model (HMM) profile, which was download from the Pfam protein family database (http://pfam.xfam.org/) (accessed on 4 December 2019). To ensure the authenticity of each member in TF families, all sequences were double-checked using a batch CD-search based on the NCBI conserved domain database (http://www.ncbi.nlm.nih.gov/Structure/cdd/cdd.shtml) (accessed on 4 December 2019) and the Pfam protein database. Finally, all TF amino acid sequences were blast to NCBI (http://blast.ncbi.nlm.nih.gov/Blast.cgi) (accessed on 7 December 2019) to search for their homology.

### 4.7. Multiple Sequence Alignments and Phylogenetic Analysis

To infer the phylogenetic relationships, multiple sequence alignments of peanut TFs along with reference TF proteins of *Arabidopsis thaliana*, *Oryza sativa,* and *Glycine max* were conducted using Clustal W with default settings. The amino acid sequences of these well-characterized model species were downloaded from phytozome v12.1 (https://phytozome.jgi.doe.gov/pz/portal.html) (accessed on 13 December 2019). Subsequently, the unrooted phylogenetic trees based on amino acid sequences were constructed using the MEGA v 7.0 software; the neighbor-joining (NJ) method was selected and the parameters were as follows: JTTmodel, pairwise gap deletion, and 1000 bootstraps [63].

### 4.8. Protein Characterization and Conversed Motif Analysis

The amino acid length, molecular weight, theoretical isoelectric points, instability index, and aliphatic index of all TF amino sequences were analyzed using ProtParam tool available on Expert Protein Analysis System (ExPASy) (http://web.expasy.org/protparam/) (accessed on 17 December 2019). The subcellular localization of all TF members was predicted by the CELLO online tool v 2.5 (http://cello.life.nctu.edu.tw/) (accessed on 17 December 2019) [64].

The conserved motif analysis of all TF amino sequences was carried out using the Multiple Expectation-maximization for Motif Elicitation (MEME) program (http://meme-suite.org/tools/meme) (accessed on 15 December 2019), the parameters were as follows: maximum number of motifs was 10, motif length was 6–100, motif sites was 2–120.

### 4.9. Expression Profile and Functional Enrichment Analyses

The temporal expression profiles of peanut TFs under cold stress were characterized based on the RNA-Seq data. The quantification of gene expression levels was estimated by the values of fragments per kilobase of transcript per million fragments mapped (FPKM) [65]. The formula is shown as follow:

(1) $$\mathrm{FPKM}=\frac{\mathrm{cDNA}\;\mathrm{Fragments}}{\mathrm{Mapped}\;\mathrm{Fragments}\;\left(\mathrm{Millions}\right)\times \mathrm{Transcript}\;\mathrm{Lenghth}\;\left(\mathrm{kb}\right)}$$

The cluster heat maps of gene expression were depicted by Cluster (http://bonsai.hgc.jp/~mdehoon/software/cluster/software.htm) (accessed on 22 December 2019) and Tree view (http://jtreeview.sourceforge.net/) (accessed on 22 December 2019); the red regions indicated upregulation and the blue regions indicated downregulation.

To gain insights into the functional versatility of the identified TFs, Gene ontology (GO) enrichment analysis was carried out. GO annotation was implemented by the GOseq R package, in which gene length bias was corrected. GO terms with corrected *p*-value < 0.05 were considered significantly enriched [66].

### 4.10. Protein Interactions and Module Analysis

In order to explore the interactions between TFs and genes, the protein–protein interaction (PPI) analysis was carried out based on the STRING database (https://string-db.org/) (accessed on 24 December 2019) in Clusters of Orthologous Group (COG) mode [67]. The well-characterized model plant *Arabidopsis thaliana* was the query organism, and a combined score of ≥0.4 was used as the cut-off value. Moreover, the use of Cytoscape software v 3.7.2 was to construct PPI networks [68].

In order to obtain the core network of each TF family and hub genes, the module analysis of PPI networks was performed using the Cytoscape plug-in Molecular Complex Detection (MCODE), the parameters were as follows: degree cutoff is 2, node score cutoff is 0.2, and K-core is 2.

### 4.11. Statistical Analysis

All statistical analyses of physiological and metabolic data were performed with SPSS version 19.0 (SPSS Inc.) using a one-way analysis of variance (ANOVA). Mean separations were performed using least significant difference (SD). The difference at *p* < 0.05 level was considered significant, and the difference at *p* < 0.01 level was remarkably significant.

## Figures and Tables

**Table 1:** List of peanut transcription factor families identified in the transcriptome analysis.

Serial Number	TF Family	Number of Genes	Serial Number	TF Family	Number of Genes	Serial Number	TF Family	Number of Genes
1	bHLH	144	30	MIKC_MADS	29	59	PLATZ	9
2	C2H2	129	31	TCP	23	60	SRS	9
3	MYB-related	95	32	IWS1	22	61	LSD	8
4	WRKY	95	33	Tify	22	62	SWI3	8
5	MYB	92	34	LOB	21	63	WOX	8
6	C3H	86	35	BAF60b	20	64	AP2	6
7	ERF	79	36	ARID	18	65	Coactivator p15	6
8	bZIP	74	37	RWP-RK	18	66	DBB	6
9	NAC	73	38	HB-other	17	67	GeBP	6
10	SET	73	39	CO-like	16	68	LIM	6
11	SNF2	72	40	NF-YA	16	69	ZF-HD	6
12	HD-ZIP	67	41	TUB	16	70	MBF1	5
13	G2-like	58	42	NF-YC	15	71	DBP	4
14	GNAT	53	43	HMG	13	72	EIL	4
15	PHD	53	44	M-type MADS	13	73	MED7	4
16	FAR1	47	45	Alfin-like	12	74	NF-X1	4
17	ARF	43	46	CAMTA	12	75	Rcd1-like	4
18	B3	42	47	E2F/DP	12	76	S1Fa-like	4
19	Trihelix	42	48	Pseudo ARR-B	12	77	VOZ	4
20	GRAS	38	49	ARR-B	11	78	Whirly	4
21	mTERF	38	50	BES1	11	79	CSD	3
22	AUX/IAA	37	51	GRF	11	80	HB-PHD	3
23	Dof	35	52	LUG	11	81	OFP	3
24	HSF	34	53	TAZ	11	82	RAV	3
25	SBP	33	54	BBR-BPC	10	83	ULT	3
26	TALE	33	55	CPP	10	84	MED6	2
27	TRAF	33	56	DDT	10	85	RB	2
28	GATA	32	57	YABBY	10	86	SOH1	2
29	Jumonji	29	58	NF-YB	9	87	STAT	2
							Total	2328

## Supplementary Materials

The following are available online at https://www.mdpi.com/1422-0067/21/6/1921/s1. Figure S1: Quantitative real-time PCR (qRT-PCR) validation. Figure S2: Phylogenetic relationships and subfamily designations of peanut TF families along with model species Arabidopsis thaliana, Oryza sativa, and Glycine max. Figure S3: Conserved motifs of six peanut TF families. Figure S4: Motif logos of peanut TFs obtained from MEME analysis. Table S1: Summary of RNA-Seq reads and their mapping on the peanut genome. Table S2: Blast hit results and gene expression of 2328 peanut transcriptional factors. Table S3: The 1125 transcriptional factors differentially expressed in NH5 and FH18 under cold stress. Table S4: List of 445 transcriptional factors (TFs) in “cold-tolerant TF set”. Table S5: Description of protein properties and predicted subcellular localization of peanut transcription factors. Table S6: Information about six protein–protein interaction networks. Table S7: List of primers used in this study for quantitative real-time PCR.

## Abbreviations

TF	Transcription factor
PPI	Protein-protein interaction
JA	Jasmonic acid
SA	Salicylic acid
CBFs/DREB1s	C-repeat binding factors
DREB1s	Dehydration responsive element binding factor1 proteins
AP2	APETALA2
ERF	ETHYLENE RESPONSIVE FACTOR
COR	Cold responsive genes
ICE1	Inducer of CBF expression 1
CAMTA	Calmodulin binding transcription activator
PIF	Phytochrome-interacting factor
PH	Plant height
TLA	Total leaf area
SFW	Shoot fresh weight
RFW	Root fresh weight
SDW	Shoot dry weight
RDW	Root dry weight
EL	Electrolyte leakage
MDA	Malondialdehyde
FDR	False discovery rate
GO	Gene Ontology
KEGG	Kyoto Encyclopedia of Genes and Genomes
ABA	Abscisic acid
MAPK	Mitogen-activated protein kinase
ET	Ethylene
EIN3	Ethylene-insensitive 3
FPKM	Fragments per kilobase of transcript per million fragments mapped
bHLH	Basic helix—loop—helix protein
C2H2	Cys2/His2 zinc finger protein
ERF	Ethylene-responsive factor
MYB	v-myb avian myeloblastosis viral oncogene homolog
NAC	NAM, ATAF1/2, CUC2
