# Supplementary material for: Comparative Transcriptome-Based Mining and Expression Profiling of Transcription Factors Related to Cold Tolerance in Peanut
Source: Int J Mol Sci. 2020 Mar 11;21(6):1921. doi: 10.3390/ijms21061921 (PMC7139623; doi:10.3390/ijms21061921)
Supplement: Supplementary file 1 [file ijms-21-01921-s001.zip › Supplementary Material/Figure S1.pdf]

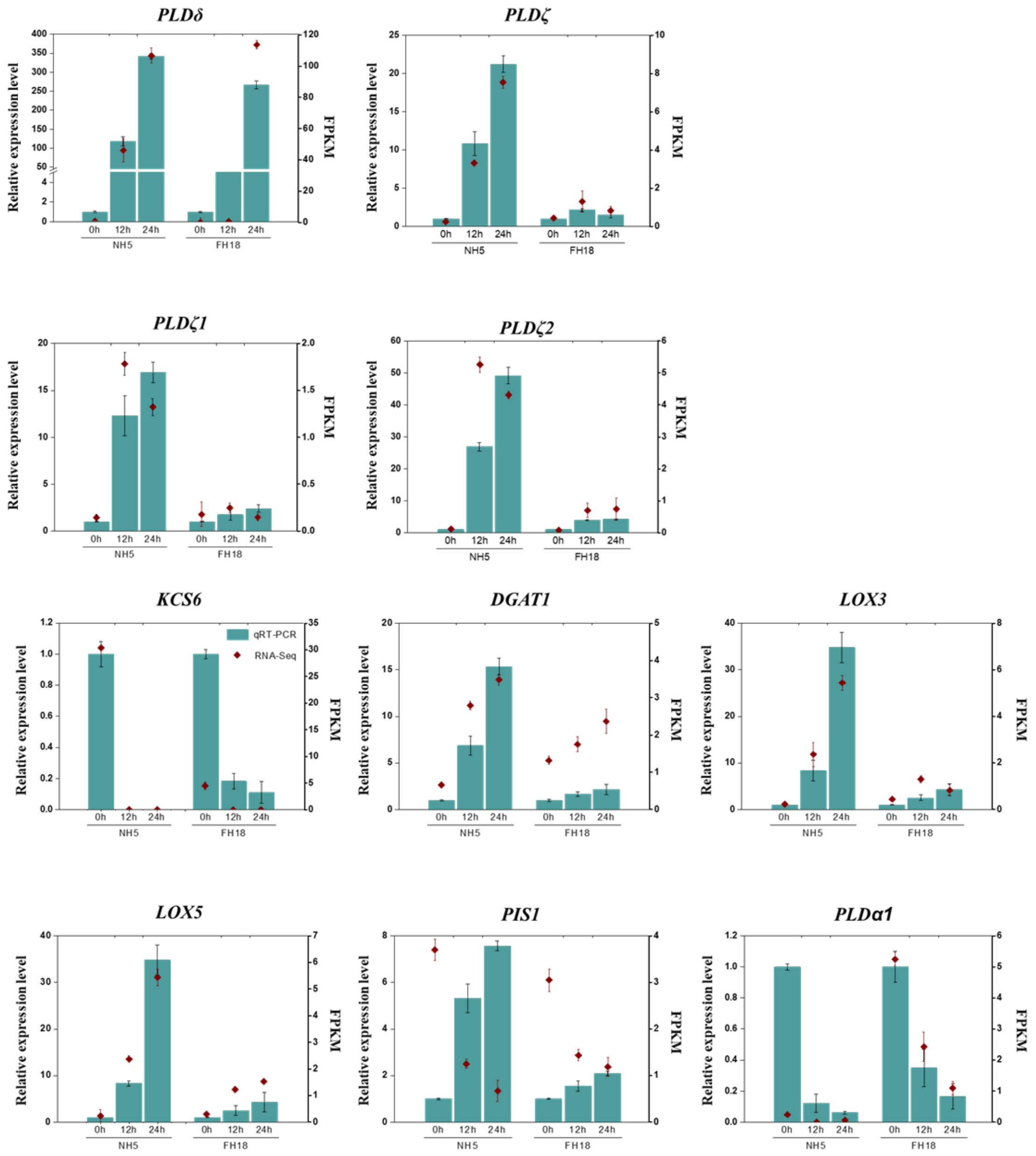

Figure S1. Quantitative real-time PCR (qRT-PCR) validation. The green columns represent qRT- PCR validation and the red spots represent RNA-Seq data. Error bars represent the SD of the means (n=3). DGAT, diacylglycerol acyltransferase; KCS1, 3-ketoacyl-CoA synthase; LOX, lipoxygenase; PIS, CDP-diacylglycerol--inositol 3-phosphatidyltransferase; PLD, phospholipase D.
