# Supplementary material for: Comparative Transcriptome-Based Mining and Expression Profiling of Transcription Factors Related to Cold Tolerance in Peanut
Source: Int J Mol Sci. 2020 Mar 11;21(6):1921. doi: 10.3390/ijms21061921 (PMC7139623; doi:10.3390/ijms21061921)
Supplement: Supplementary file 1 [file ijms-21-01921-s001.zip › Supplementary Material/Figure S2.pdf]

Figure S2. Phylogenetic relationships and subfamily designations of peanut TF families along with model species *Arabidopsis thaliana*, *Oryza sativa* and *Glycine max*. The colored branch indicates the different subfamilies. Red dots indicate peanut TF genes, grey dots indicate other species TF genes.

bHLH

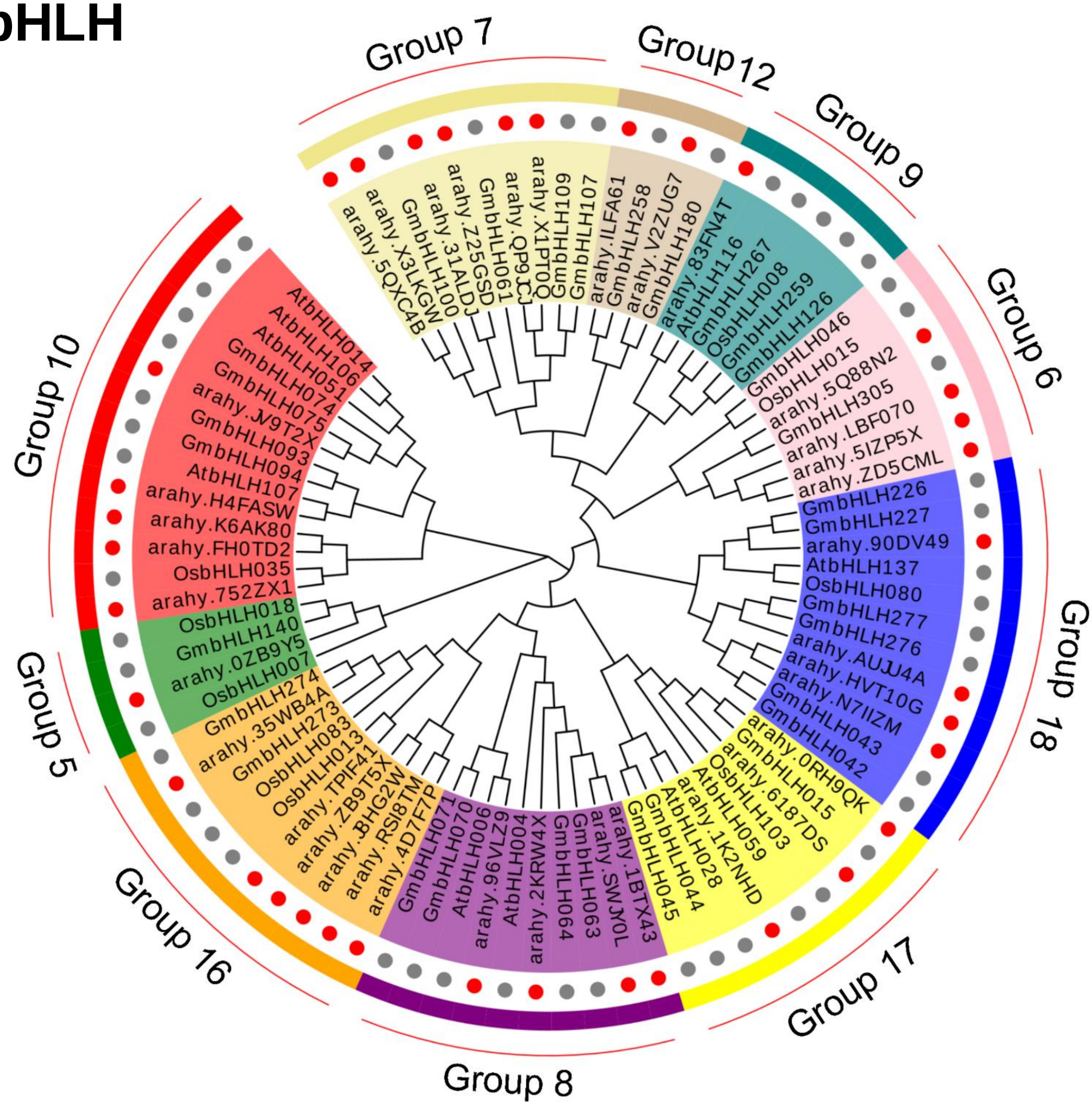

C2H2

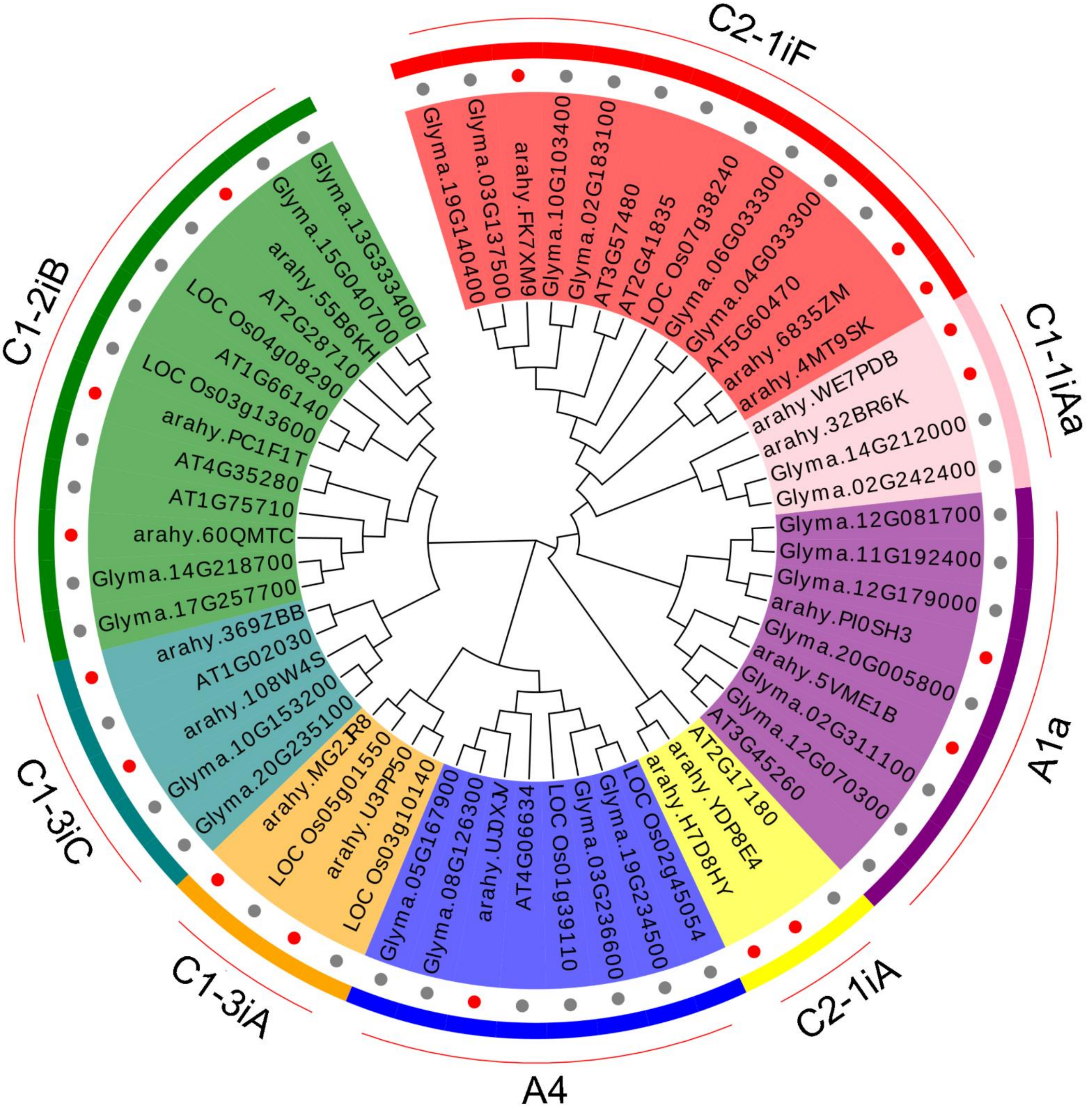
C2-1iF
Glyma.19G140400
Glyma.03G137500
arahy.FK7XM9
Glyma.10G103400
Glyma.02G183100
AT3G57480
AT2G41835
LOC Os07g38240
Glyma.06G033300
Glyma.04G033300
AT5G60470
arahy.6835ZM
arahy.4MT9SK
C1-1iAa
arahy.WE7PDB
arahy.32BR6K
Glyma.14G212000
Glyma.02G242400
A1a
Glyma.12G081700
Glyma.11G192400
Glyma.12G179000
arahy.PI0SH3
Glyma.20G005800
arahy.5VME1B
Glyma.02G311100
Glyma.12G070300
AT3G45260
C2-1iA
AT2G17180
arahy.YDP8E4
arahy.H7D8HY
A4
LOC Os02g45054
Glyma.19G234500
Glyma.03G236600
LOC Os01g39110
AT4G06634
arahy.UJJXJV
Glyma.08G126300
Glyma.05G167900
C1-3iA
LOC Os03g10140
arahy.U3PP50
LOC Os05g01550
arahy.MG2JR8
C1-3iC
Glyma.20G235100
Glyma.10G153200
arahy.108W4S
AT1G02030
arahy.369ZBB
C1-2iB
Glyma.17G257700
Glyma.14G218700
arahy.60QMTC
AT1G75710
AT4G35280
arahy.PC1F1T
LOC Os03g13600
AT1G66140
LOC Os04g08290
AT2G28710
arahy.55B6KH
Glyma.15G040700
Glyma.13G333400

ERF

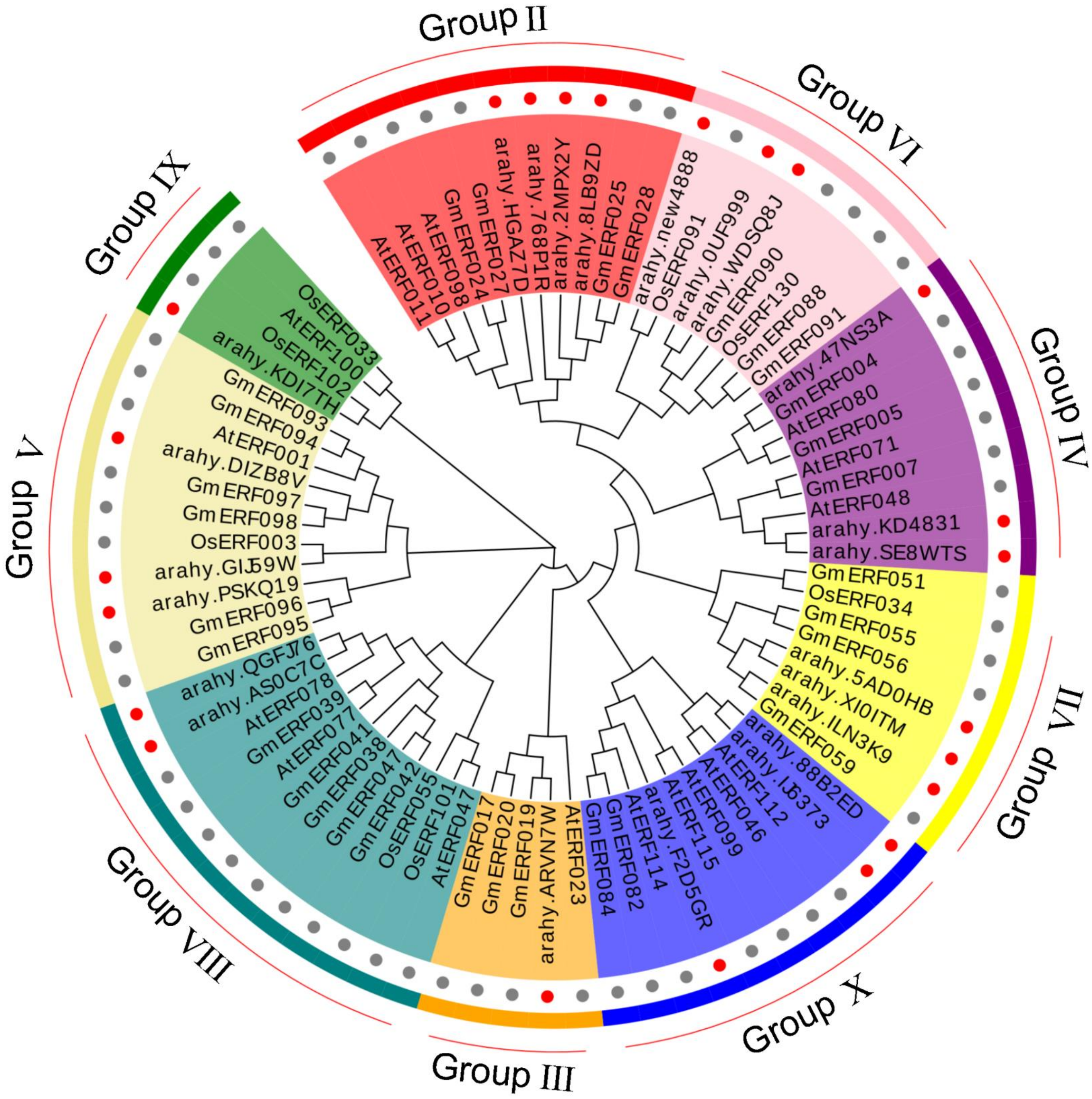
Group II
Group VI
Group IV
Group VII
Group X
Group III
Group VIII
Group V
Group IX
AtERF011
AtERF010
AtERF098
GmERF024
GmERF027
arahy.HGAZ7D
arahy.768P1R
arahy.2MPX2Y
arahy.8LB9ZD
GmERF025
GmERF028
arahy.new4888
OsERF091
arahy.0UF999
arahy.WDSQ8J
GmERF090
OsERF130
GmERF088
GmERF091
arahy.47NS3A
GmERF004
AtERF080
GmERF005
AtERF071
GmERF007
AtERF048
arahy.KD4831
arahy.SE8WTS
GmERF051
OsERF034
GmERF055
GmERF056
arahy.5AD0HB
arahy.XI0ITM
arahy.ILN3K9
GmERF059
arahy.88B2ED
AtERF112
AtERF046
AtERF099
AtERF115
arahy.F2D5GR
AtERF114
GmERF082
GmERF084
AtERF023
arahy.ARVN7W
GmERF019
GmERF020
GmERF017
AtERF047
OsERF101
OsERF055
GmERF042
GmERF047
GmERF038
GmERF041
AtERF077
GmERF039
AtERF078
arahy.AS0C7C
arahy.QGFJ76
GmERF095
GmERF096
arahy.PSKQ19
arahy.GIJ59W
OsERF003
GmERF098
GmERF097
arahy.DIZB8V
AtERF001
GmERF094
GmERF093
arahy.KDI7TH
OsERF102
AtERF100
OsERF033

MYB

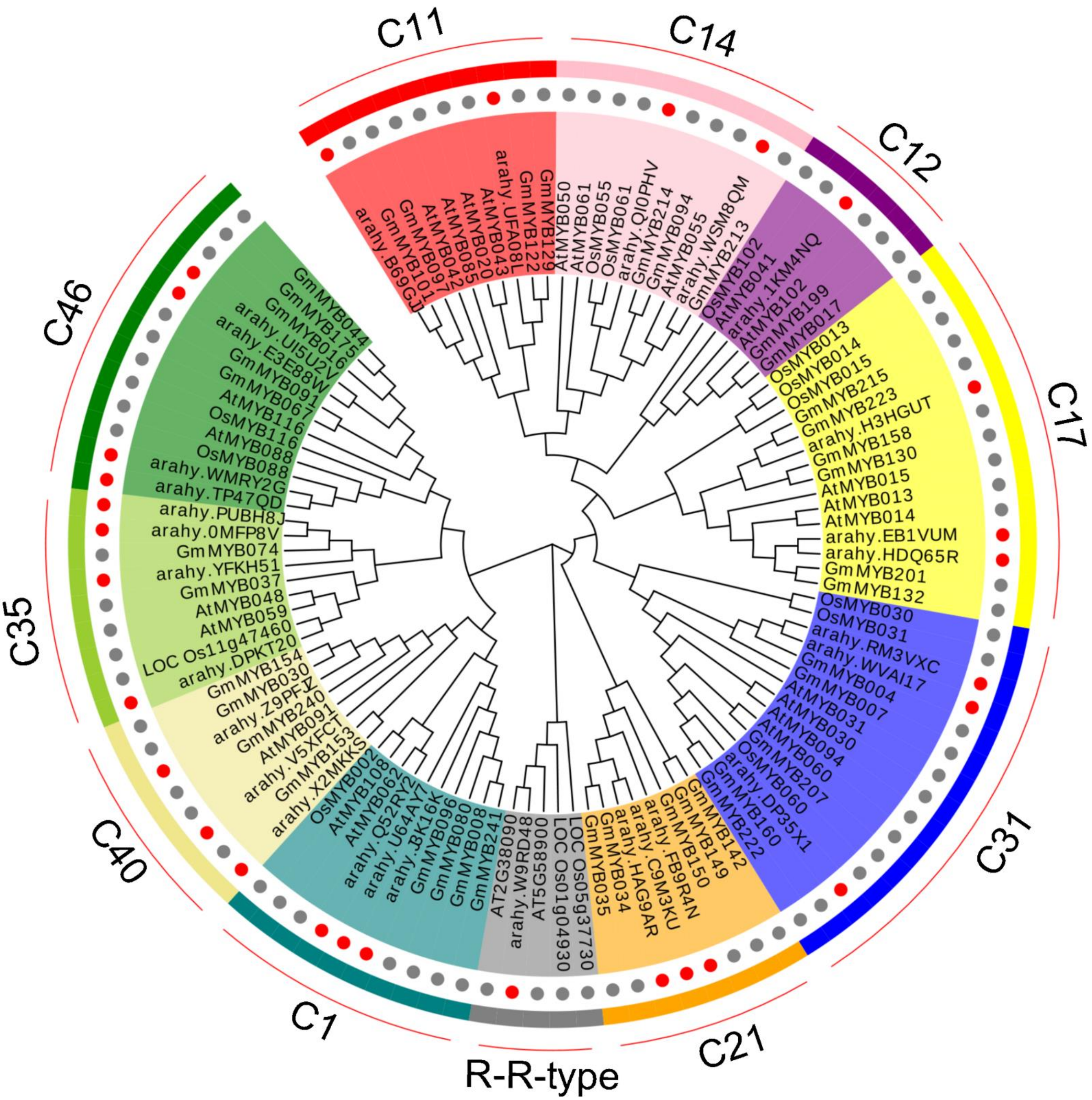
C11
C14
C12
C17
C31
C21
R-R-type
C1
C40
C35
C46
Gm MYB129
Gm MYB122
arahy.UFA08L
AtMYB043
AtMYB020
AtMYB085
AtMYB042
AtMYB097
Gm MYB101
arahy.B69GJI
AtMYB050
AtMYB061
OsMYB055
OsMYB061
arahy.QI0PHV
Gm MYB214
AtMYB094
AtMYB055
arahy.WSM8QM
Gm MYB213
OsMYB102
AtMYB041
arahy.1KM4NQ
AtMYB102
Gm MYB199
Gm MYB017
OsMYB013
OsMYB014
OsMYB015
Gm MYB215
Gm MYB223
arahy.H3HGUT
Gm MYB158
Gm MYB130
AtMYB015
AtMYB013
AtMYB014
arahy.EB1VUM
arahy.HDQ65R
Gm MYB201
Gm MYB132
OsMYB030
OsMYB031
arahy.RM3VXC
arahy.WVAI17
Gm MYB004
Gm MYB007
AtMYB031
AtMYB030
AtMYB094
AtMYB060
Gm MYB207
OsMYB060
arahy.DP35X1
Gm MYB160
Gm MYB222
Gm MYB142
Gm MYB149
Gm MYB150
arahy.FB9R4N
arahy.C9M3KU
arahy.HAG9AR
Gm MYB034
Gm MYB035
LOC Os05g37730
LOC Os01g04930
AT5G58900
arahy.W9RD48
AT2G38090
Gm MYB241
Gm MYB008
Gm MYB080
Gm MYB096
arahy.BK16K
arahy.U64AY7
arahy.Q52RY2
AtMYB062
AtMYB108
OsMYB002
arahy.X2MKKS
Gm MYB153
arahy.V5XFCT
AtMYB091
Gm MYB240
arahy.Z9PFIZ
Gm MYB030
Gm MYB154
arahy.DPKT20
LOC Os11g47460
AtMYB059
AtMYB048
Gm MYB037
arahy.YFKH51
Gm MYB074
arahy.0MFP8V
arahy.PUBH8J
arahy.TP47QD
arahy.WMRY2G
OsMYB088
AtMYB116
OsMYB116
AtMYB067
Gm MYB091
arahy.E3E88W
arahy.UI5U2V
Gm MYB016
Gm MYB175
Gm MYB044

NAC

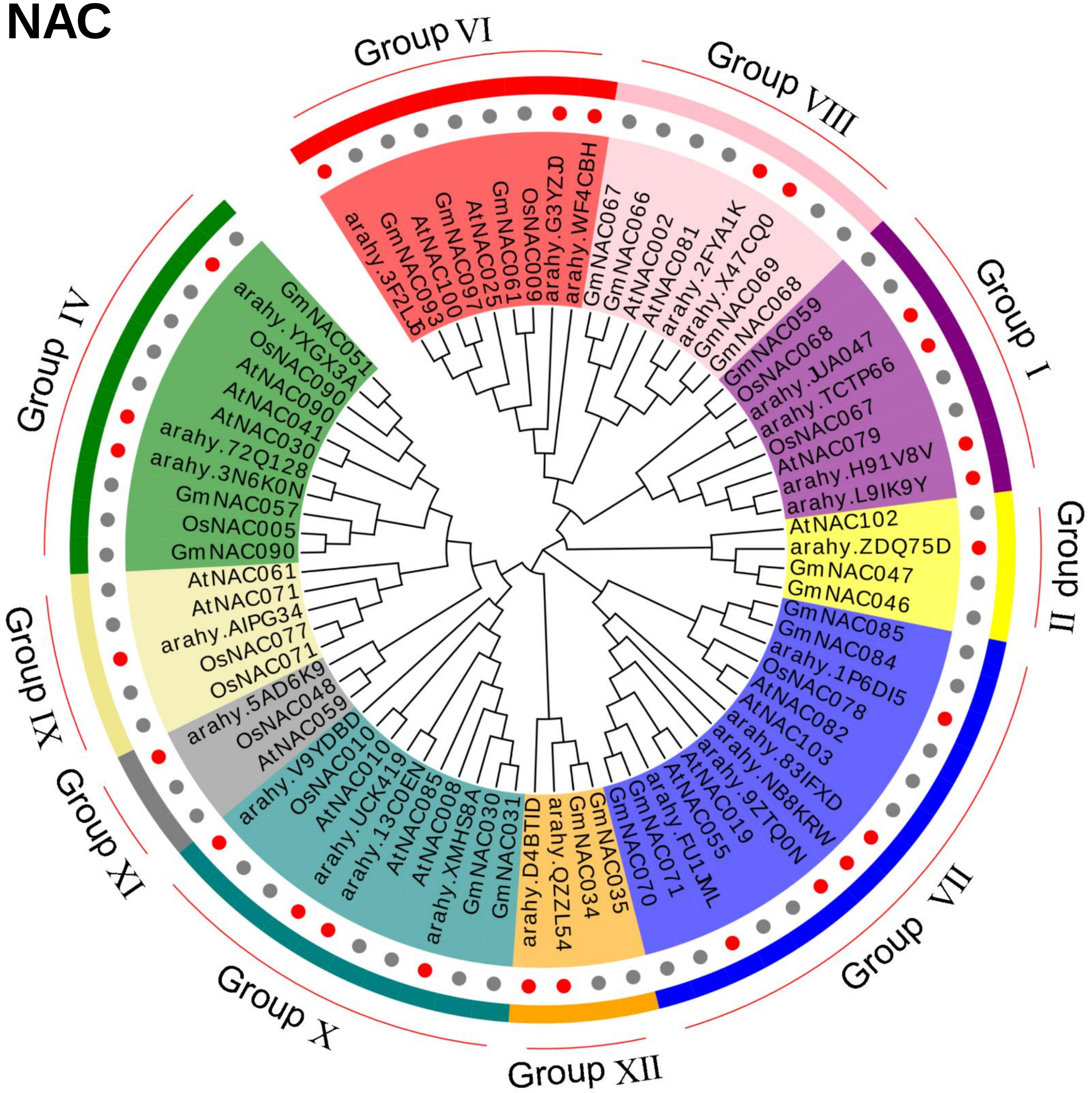
Group VI
Group VIII
Group I
Group II
Group VII
Group XII
Group X
Group XI
Group IX
Group IV
arahy.WF4CBH
OsNAC009
GmNAC061
AtNAC025
GmNAC097
AtNAC100
GmNAC093
GmNAC067
GmNAC066
AtNAC002
AtNAC081
arahy.2FYA1K
arahy.X47CQ0
GmNAC069
GmNAC068
GmNAC059
OsNAC068
arahy.TCTP66
OsNAC067
AtNAC079
arahy.H91V8V
arahy.L9IK9Y
AtNAC102
arahy.ZDQ75D
GmNAC047
GmNAC046
GmNAC085
GmNAC084
arahy.1P6DI5
OsNAC078
AtNAC082
AtNAC103
arahy.83IFXD
arahy.NB8KRW
arahy.9ZTQ0N
AtNAC019
AtNAC055
arahy.FU1JML
GmNAC071
GmNAC070
GmNAC035
GmNAC034
arahy.QZZL54
arahy.D4BTID
GmNAC031
GmNAC030
arahy.XMHS8A
AtNAC008
AtNAC085
arahy.13C0EN
arahy.UCK419
AtNAC010
OsNAC010
arahy.V9YDBD
AtNAC059
OsNAC048
arahy.5AD6K9
OsNAC071
OsNAC077
arahy.AIPG34
AtNAC071
AtNAC061
GmNAC090
OsNAC005
GmNAC057
arahy.3N6K0N
arahy.72Q128
AtNAC030
AtNAC041
AtNAC090
OsNAC090
arahy.YXGX3A
GmNAC051

WRKY

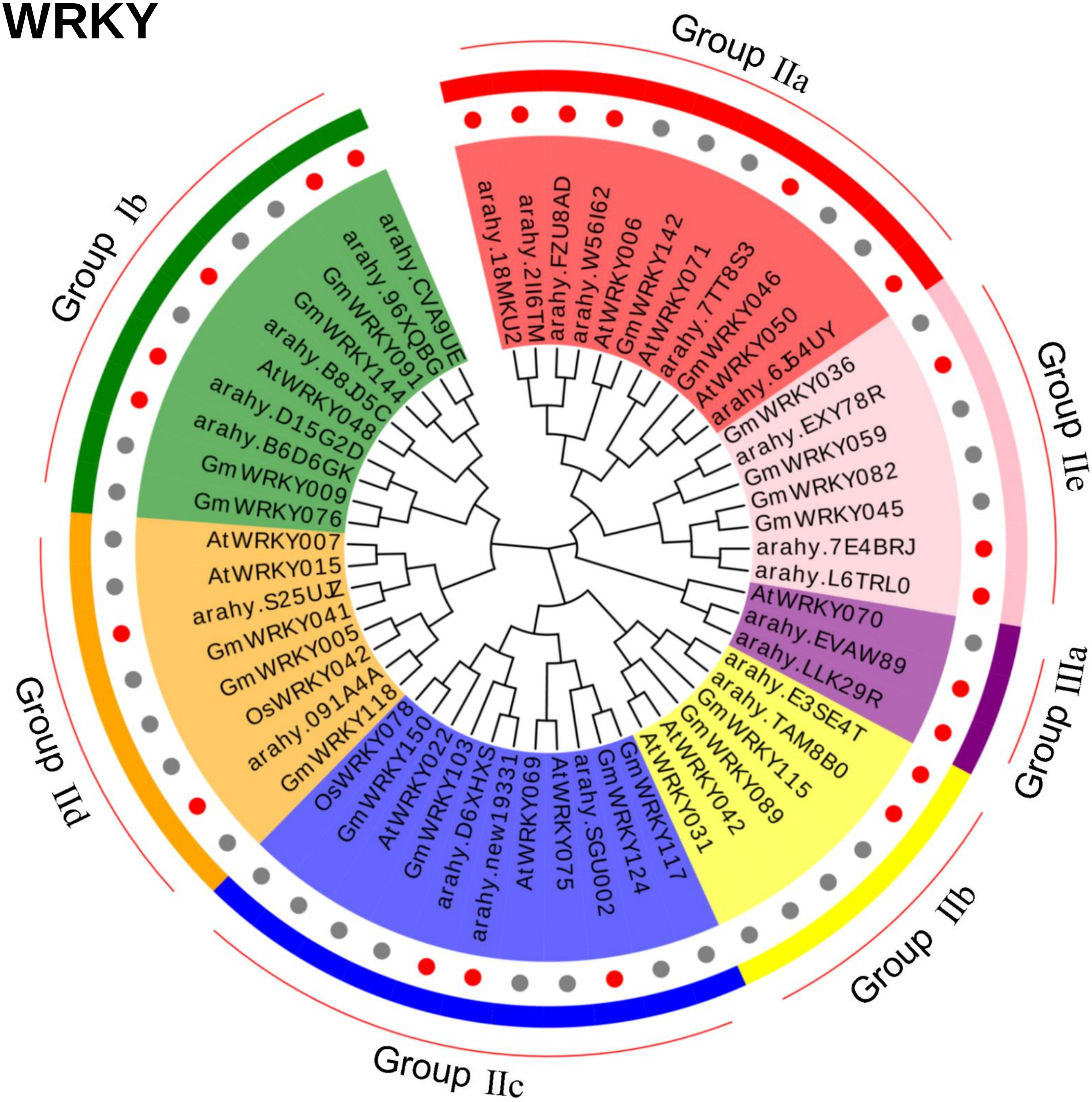
Group IIa
arahy.18MKU2
arahy.2II6TM
arahy.FZU8AD
arahy.W56I62
AtWRKY006
GmWRKY142
AtWRKY071
arahy.7TT8S3
GmWRKY046
AtWRKY050
arahy.6J54UY
Group IIe
GmWRKY036
arahy.EXY78R
GmWRKY059
GmWRKY082
GmWRKY045
arahy.7E4BRJ
arahy.L6TRL0
Group IIIa
AtWRKY070
arahy.EVAW89
arahy.LLK29R
Group IIb
arahy.E3SE4T
arahy.TAM8B0
GmWRKY115
GmWRKY089
AtWRKY042
AtWRKY031
Group IIc
GmWRKY117
GmWRKY124
arahy.SGU002
AtWRKY075
AtWRKY069
arahy.new19331
arahy.D6XHXS
GmWRKY103
AtWRKY022
GmWRKY150
OsWRKY078
Group IId
GmWRKY118
arahy.091A4A
OsWRKY042
GmWRKY005
GmWRKY041
arahy.S25UJZ
AtWRKY015
AtWRKY007
Group Ib
GmWRKY076
GmWRKY009
arahy.B6D6GK
arahy.D15G2D
AtWRKY048
arahy.B8J05C
GmWRKY144
GmWRKY091
arahy.96XQBG
arahy.CVA9UE
