# Supplementary material for: Comparative Transcriptome-Based Mining and Expression Profiling of Transcription Factors Related to Cold Tolerance in Peanut
Source: Int J Mol Sci. 2020 Mar 11;21(6):1921. doi: 10.3390/ijms21061921 (PMC7139623; doi:10.3390/ijms21061921)
Supplement: Supplementary file 1 [file ijms-21-01921-s001.zip › Supplementary Material/Figure S3.pdf]

Figure S3 Conserved motifs of six peanut TF families. All conserved motifs were statistically identified by the MEME program. The protein structures of peanut TFs based on the presence of conserved motifs were arranged corresponding to the phylogenetic tree respective of TF families. Different motifs were highlighted with different colored boxes with number 1 to 10. The sequence details of these conserved motifs were given in Figure S4.

bHLH

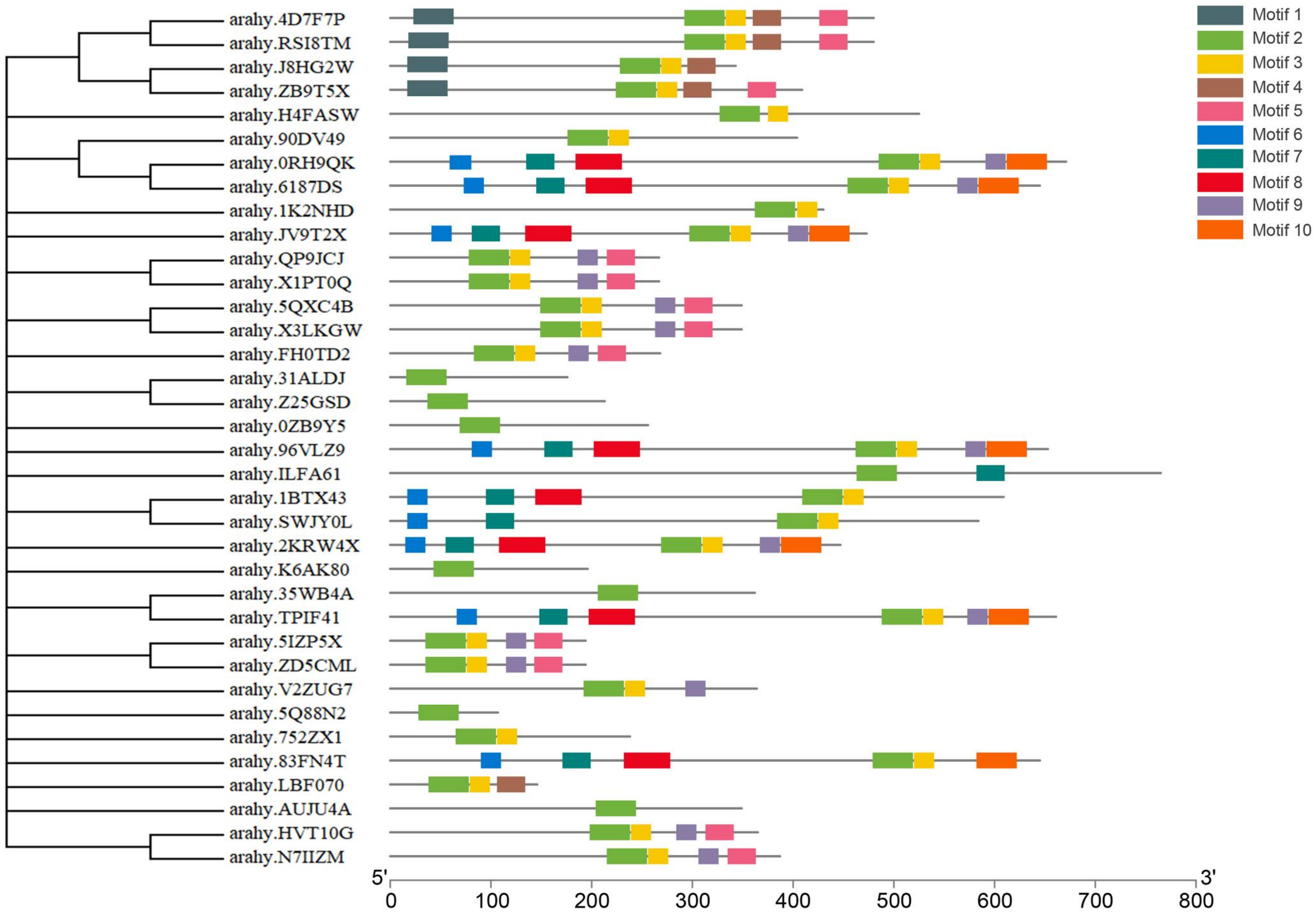

arahy.4D7F7P
arahy.RSI8TM
arahy.J8HG2W
arahy.ZB9T5X
arahy.H4FASW
arahy.90DV49
arahy.0RH9QK
arahy.6187DS
arahy.1K2NHD
arahy.JV9T2X
arahy.QP9JCJ
arahy.X1PT0Q
arahy.5QXC4B
arahy.X3LKGW
arahy.FH0TD2
arahy.31ALDJ
arahy.Z25GSD
arahy.0ZB9Y5
arahy.96VLZ9
arahy.ILFA61
arahy.1BTX43
arahy.SWJY0L
arahy.2KRW4X
arahy.K6AK80
arahy.35WB4A
arahy.TPIF41
arahy.5IZP5X
arahy.ZD5CML
arahy.V2ZUG7
arahy.5Q88N2
arahy.752ZX1
arahy.83FN4T
arahy.LBF070
arahy.AUJU4A
arahy.HVT10G
arahy.N7IIZM
Motif 1
Motif 2
Motif 3
Motif 4
Motif 5
Motif 6
Motif 7
Motif 8
Motif 9
Motif 10
5'
3'
0
100
200
300
400
500
600
700
800

C2H2

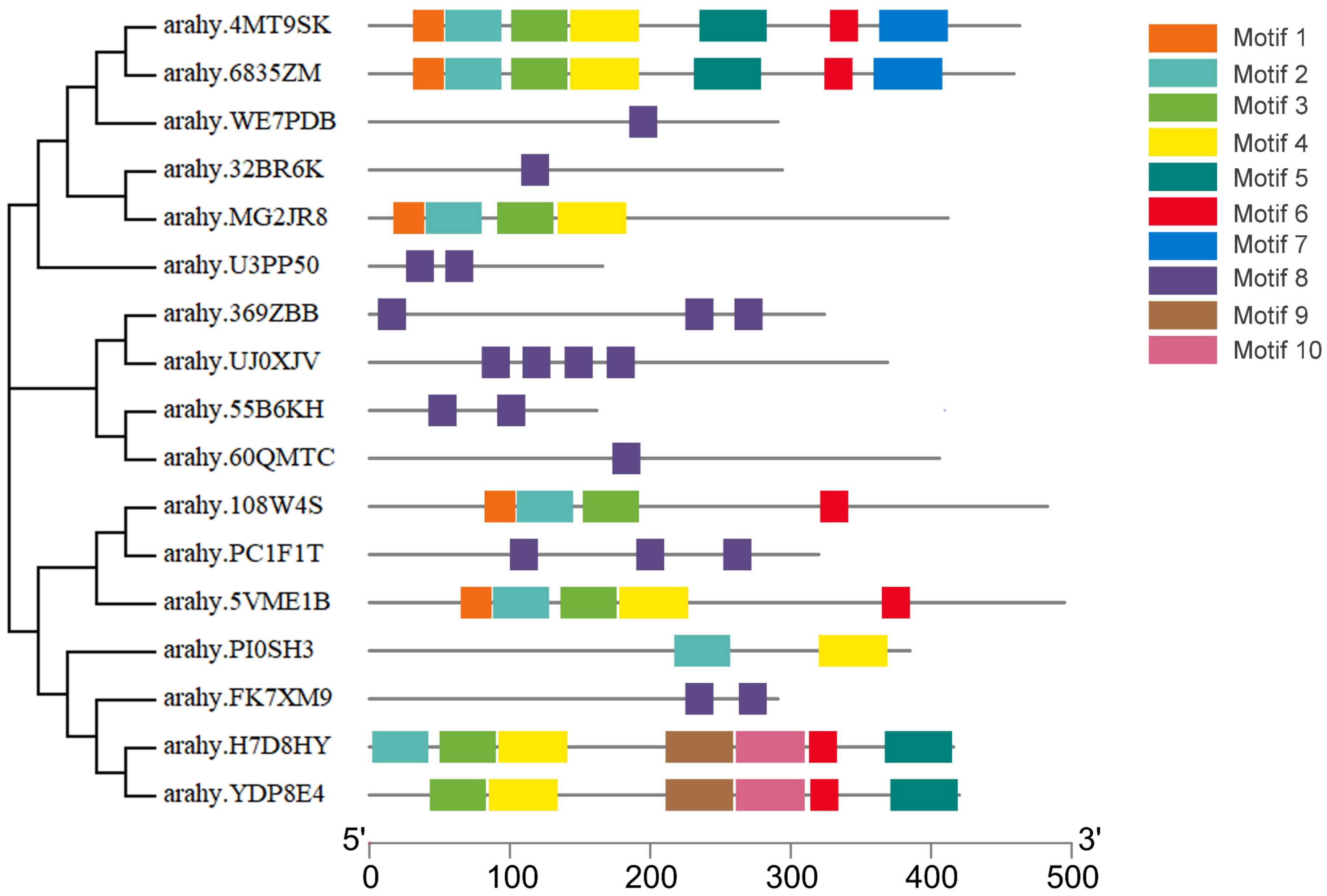

arahy.4MT9SK
arahy.6835ZM
arahy.WE7PDB
arahy.32BR6K
arahy.MG2JR8
arahy.U3PP50
arahy.369ZBB
arahy.UJ0XJV
arahy.55B6KH
arahy.60QMTC
arahy.108W4S
arahy.PC1F1T
arahy.5VME1B
arahy.PI0SH3
arahy.FK7XM9
arahy.H7D8HY
arahy.YDP8E4
Motif 1
Motif 2
Motif 3
Motif 4
Motif 5
Motif 6
Motif 7
Motif 8
Motif 9
Motif 10
5'
3'
0
100
200
300
400
500

ERF

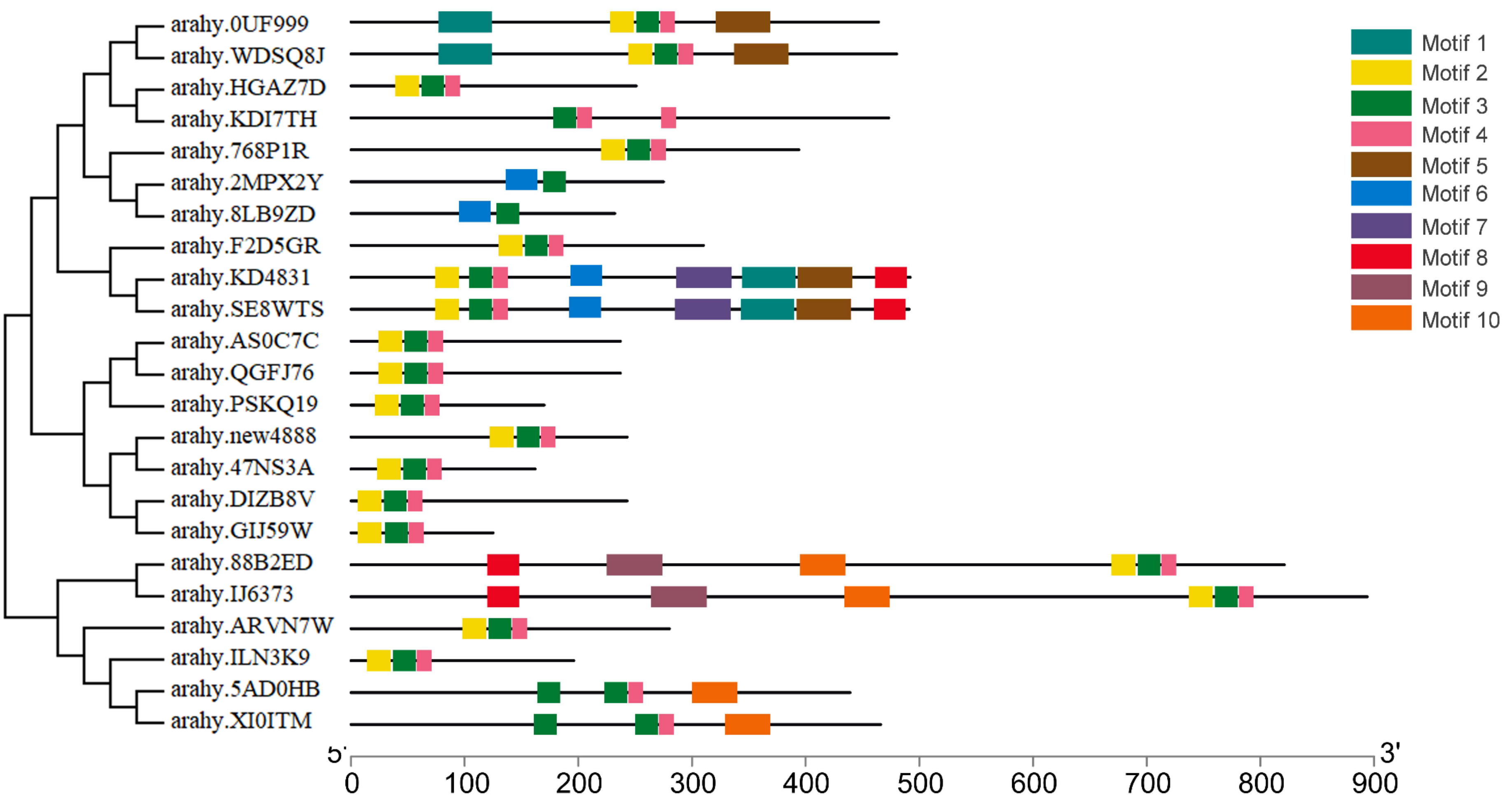

arahy.0UF999
arahy.WDSQ8J
arahy.HGAZ7D
arahy.KDI7TH
arahy.768P1R
arahy.2MPX2Y
arahy.8LB9ZD
arahy.F2D5GR
arahy.KD4831
arahy.SE8WTS
arahy.AS0C7C
arahy.QGFJ76
arahy.PSKQ19
arahy.new4888
arahy.47NS3A
arahy.DIZB8V
arahy.GIJ59W
arahy.88B2ED
arahy.IJ6373
arahy.ARVN7W
arahy.ILN3K9
arahy.5AD0HB
arahy.XI0ITM
5'
3'
0
100
200
300
400
500
600
700
800
900
Motif 1
Motif 2
Motif 3
Motif 4
Motif 5
Motif 6
Motif 7
Motif 8
Motif 9
Motif 10

MYB

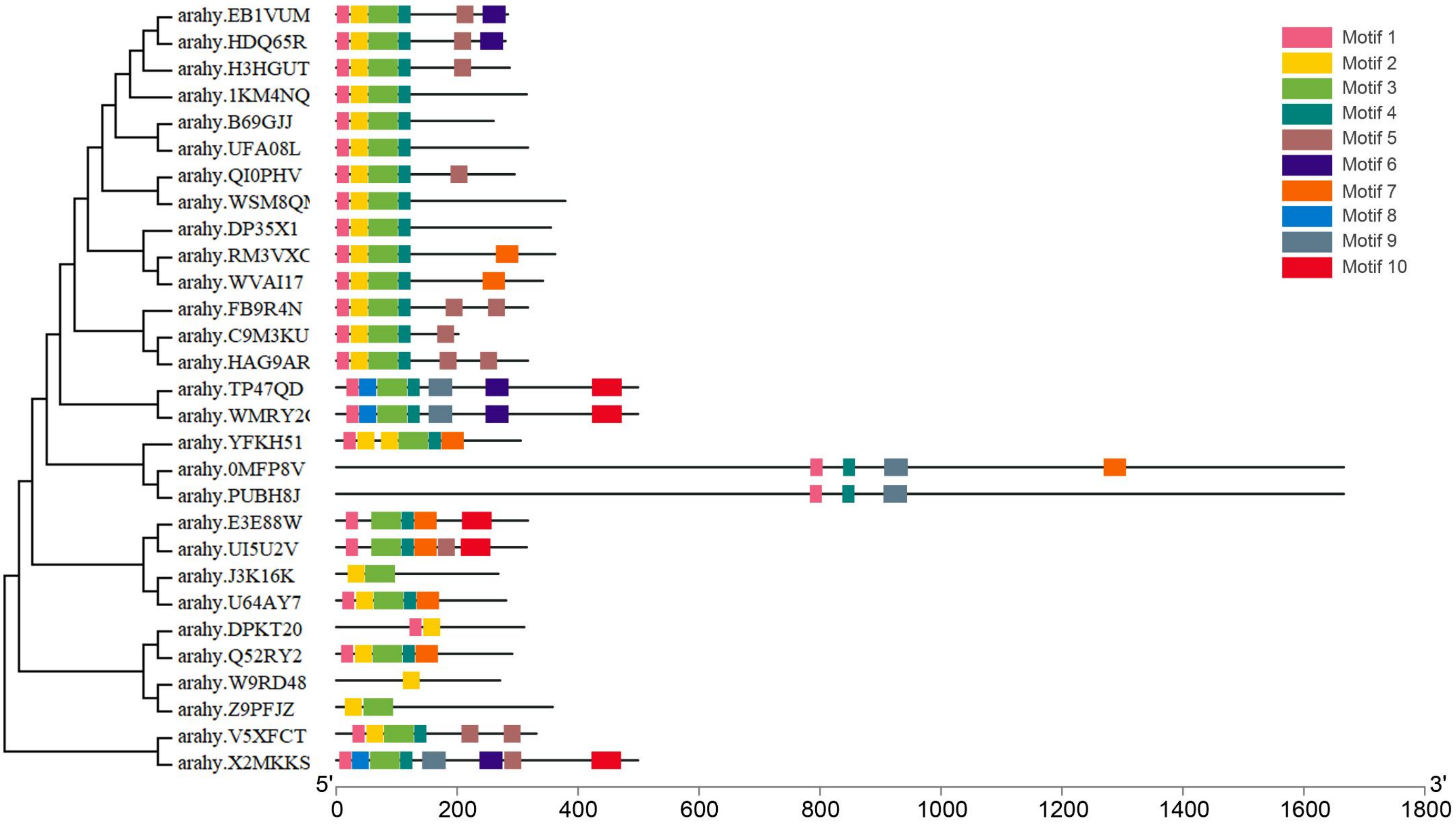

arahy.EB1VUM
arahy.HDQ65R
arahy.H3HGUT
arahy.1KM4NQ
arahy.B69GJJ
arahy.UFA08L
arahy.QI0PHV
arahy.WSM8QI
arahy.DP35X1
arahy.RM3VXC
arahy.WVAI17
arahy.FB9R4N
arahy.C9M3KU
arahy.HAG9AR
arahy.TP47QD
arahy.WMRY2C
arahy.YFKH51
arahy.0MFP8V
arahy.PUBH8J
arahy.E3E88W
arahy.UI5U2V
arahy.J3K16K
arahy.U64AY7
arahy.DPKT20
arahy.Q52RY2
arahy.W9RD48
arahy.Z9PFJZ
arahy.V5XFCT
arahy.X2MKKS
5'
3'
0
200
400
600
800
1000
1200
1400
1600
1800
Motif 1
Motif 2
Motif 3
Motif 4
Motif 5
Motif 6
Motif 7
Motif 8
Motif 9
Motif 10

NAC

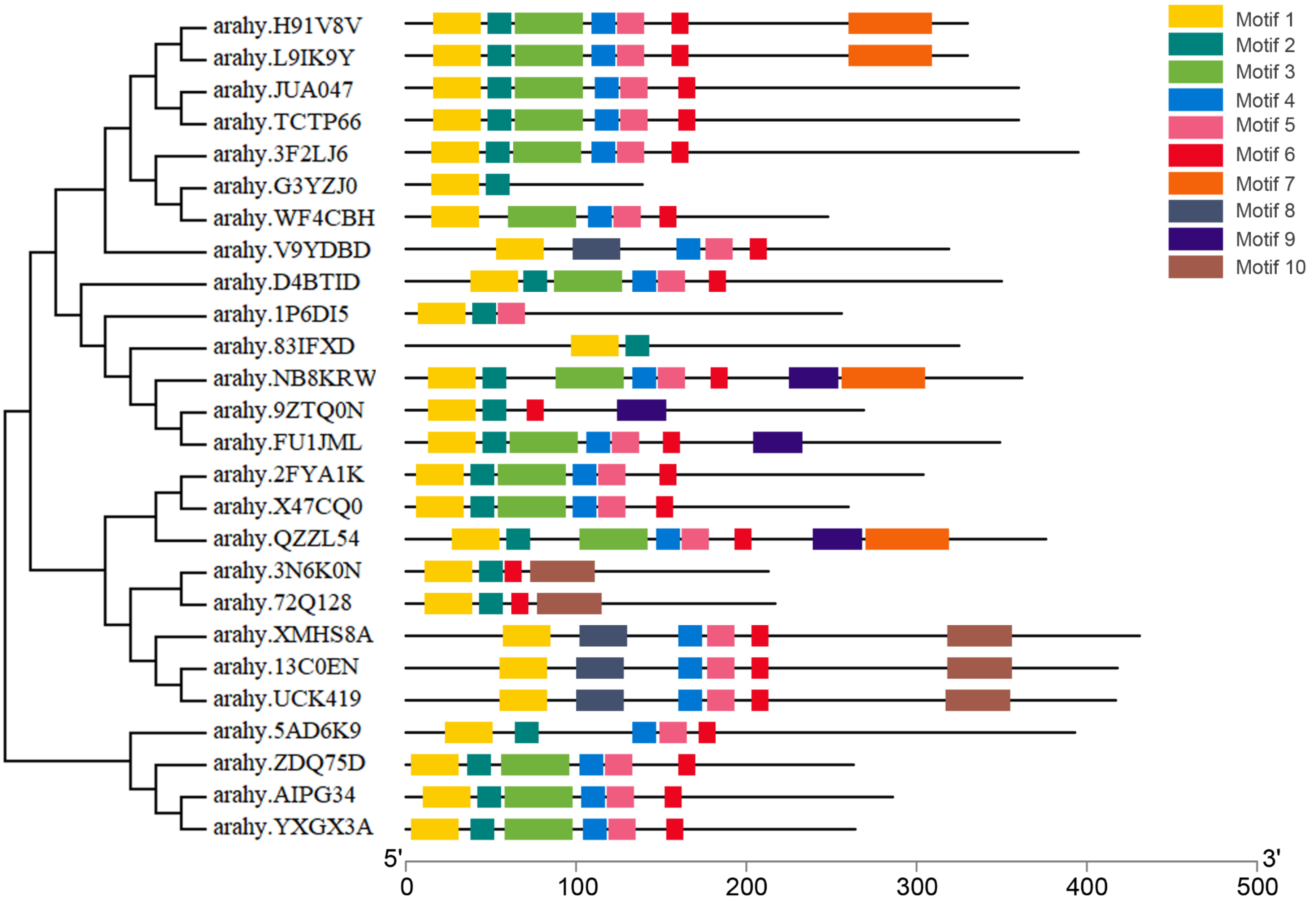

arahy.H91V8V
arahy.L9IK9Y
arahy.JUA047
arahy.TCTP66
arahy.3F2LJ6
arahy.G3YZJ0
arahy.WF4CBH
arahy.V9YDBD
arahy.D4BTID
arahy.1P6DI5
arahy.83IFXD
arahy.NB8KRW
arahy.9ZTQ0N
arahy.FU1JML
arahy.2FYA1K
arahy.X47CQ0
arahy.QZZL54
arahy.3N6K0N
arahy.72Q128
arahy.XMHS8A
arahy.13C0EN
arahy.UCK419
arahy.5AD6K9
arahy.ZDQ75D
arahy.AIPG34
arahy.YXGX3A
5'
3'
0
100
200
300
400
500
Motif 1
Motif 2
Motif 3
Motif 4
Motif 5
Motif 6
Motif 7
Motif 8
Motif 9
Motif 10

WRKY

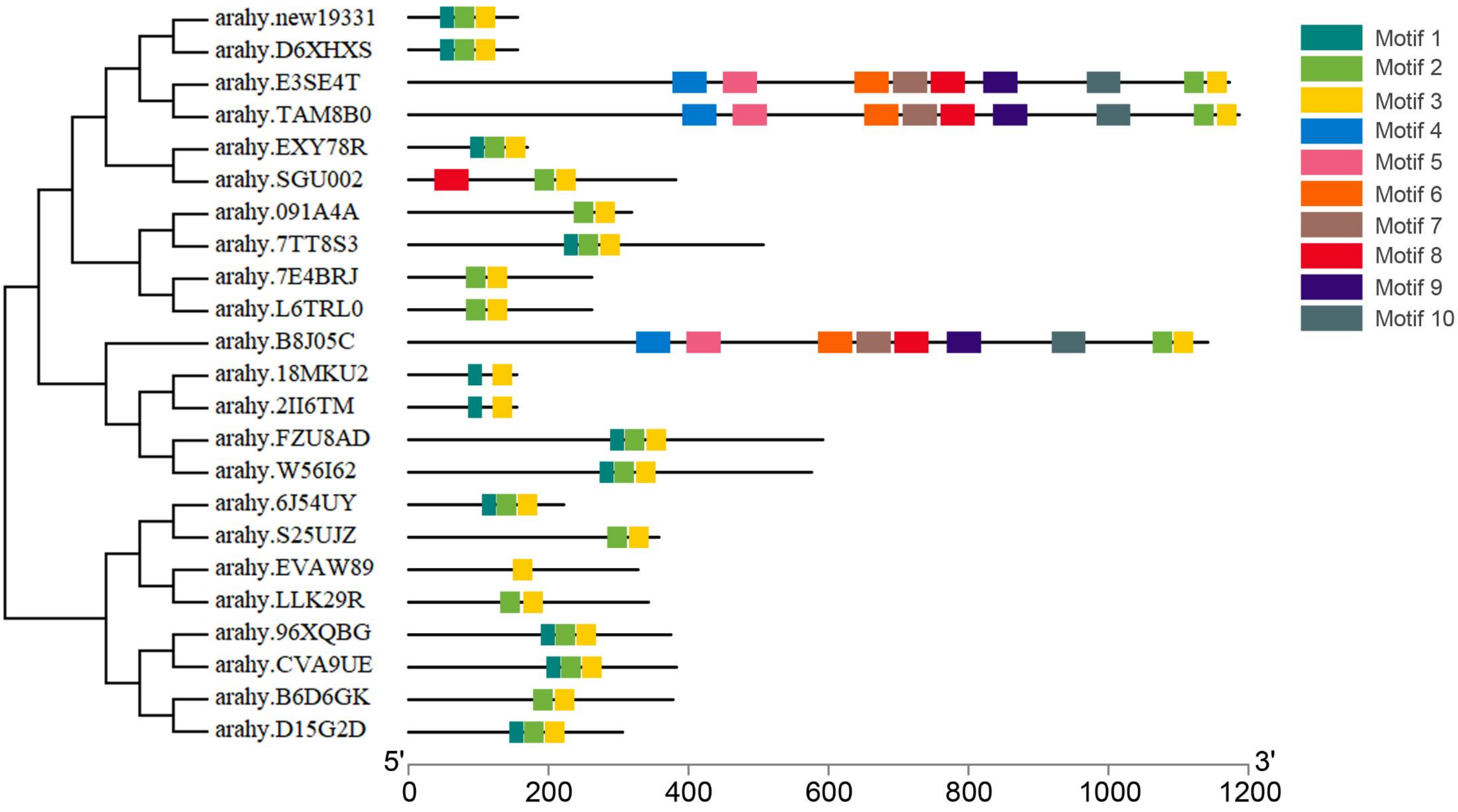
arahy.new19331
arahy.D6XHXS
arahy.E3SE4T
arahy.TAM8B0
arahy.EXY78R
arahy.SGU002
arahy.091A4A
arahy.7TT8S3
arahy.7E4BRJ
arahy.L6TRL0
arahy.B8J05C
arahy.18MKU2
arahy.2II6TM
arahy.FZU8AD
arahy.W56I62
arahy.6J54UY
arahy.S25UJZ
arahy.EVAW89
arahy.LLK29R
arahy.96XQBG
arahy.CVA9UE
arahy.B6D6GK
arahy.D15G2D
5'
3'
0
200
400
600
800
1000
1200
Motif 1
Motif 2
Motif 3
Motif 4
Motif 5
Motif 6
Motif 7
Motif 8
Motif 9
Motif 10
