# Supplementary material for: Comparative Transcriptome-Based Mining and Expression Profiling of Transcription Factors Related to Cold Tolerance in Peanut
Source: Int J Mol Sci. 2020 Mar 11;21(6):1921. doi: 10.3390/ijms21061921 (PMC7139623; doi:10.3390/ijms21061921)
Supplement: Supplementary file 1 [file ijms-21-01921-s001.zip › Supplementary Material/Figure S4.pdf]

Figure S4. Motif logos of peanut TFs obtained from MEME analysis

bHLH

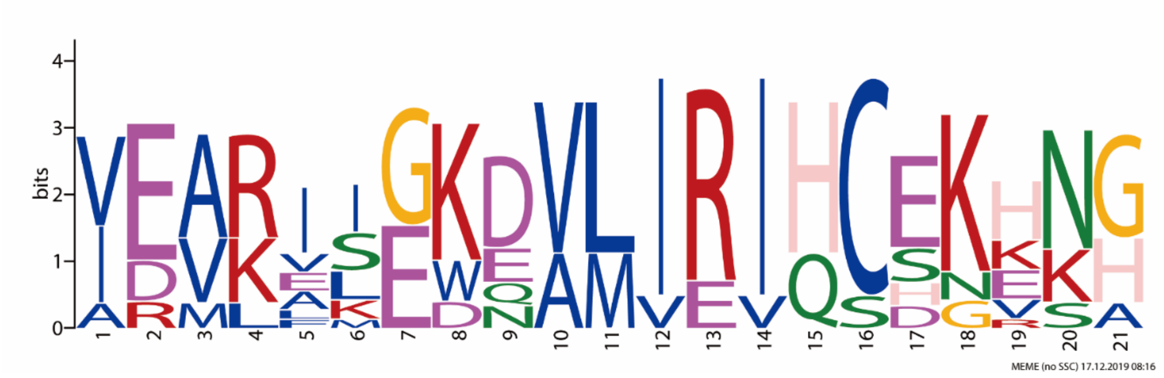

Motif 1

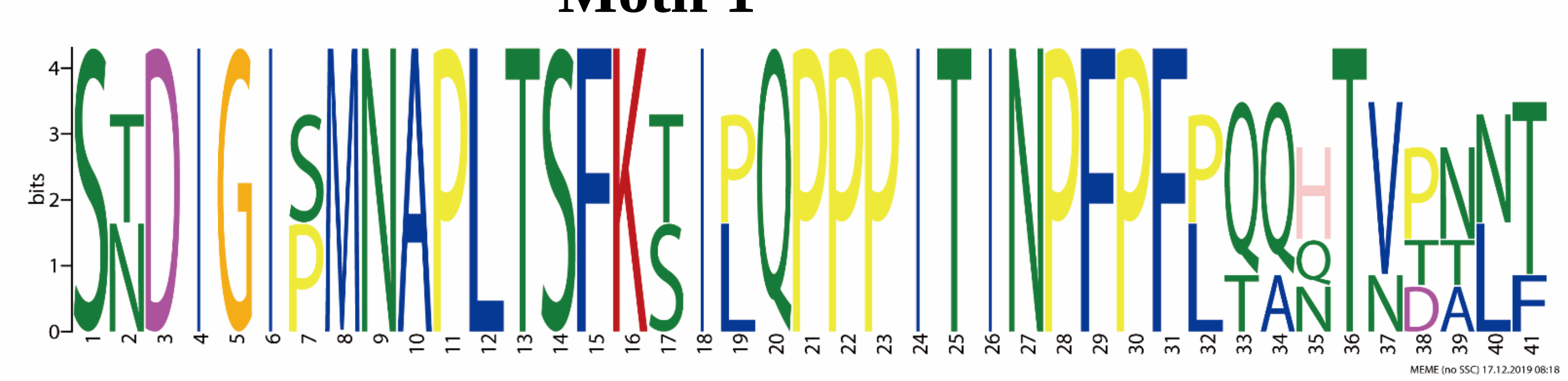

Motif 2

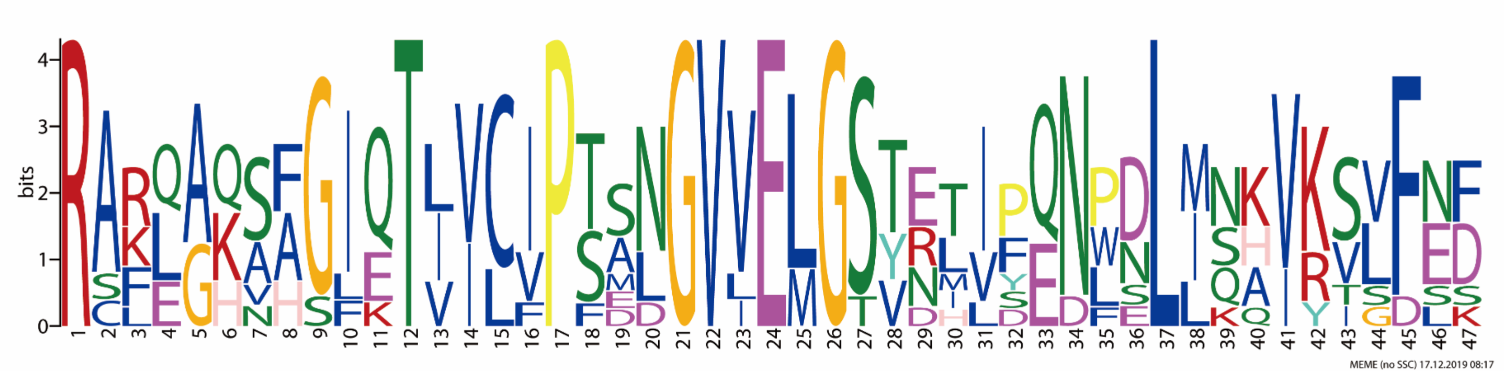

Motif 3

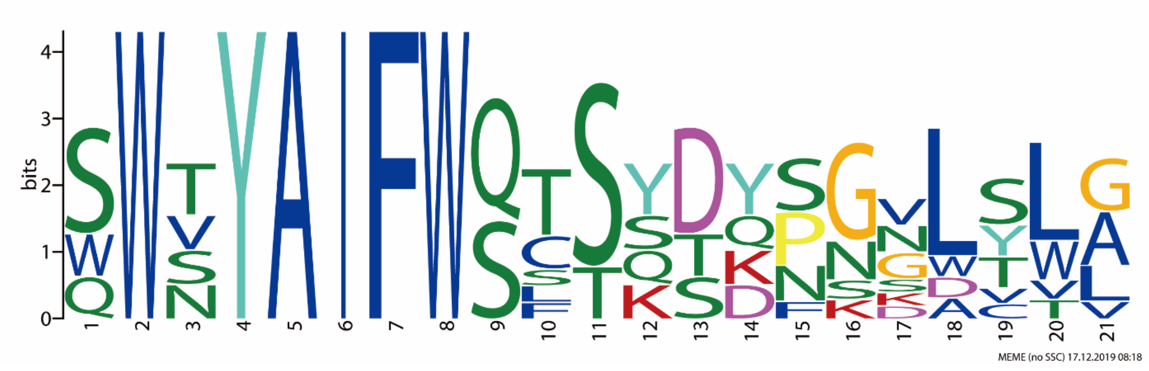

Motif 4

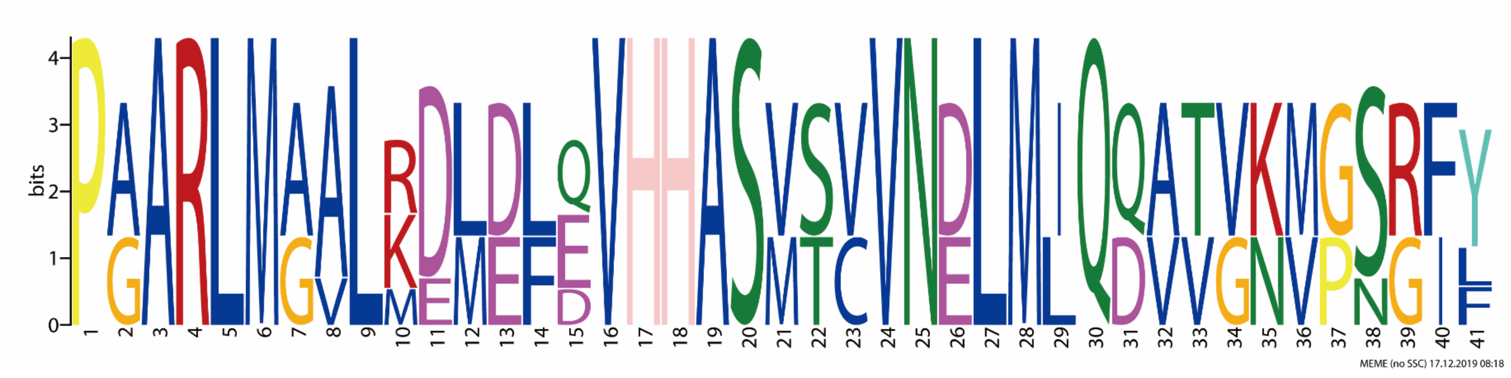

Motif 5

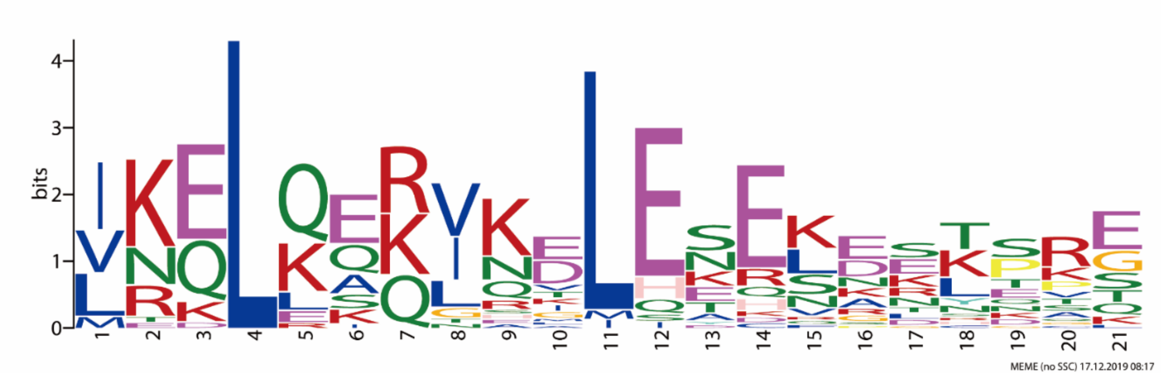

Motif 6

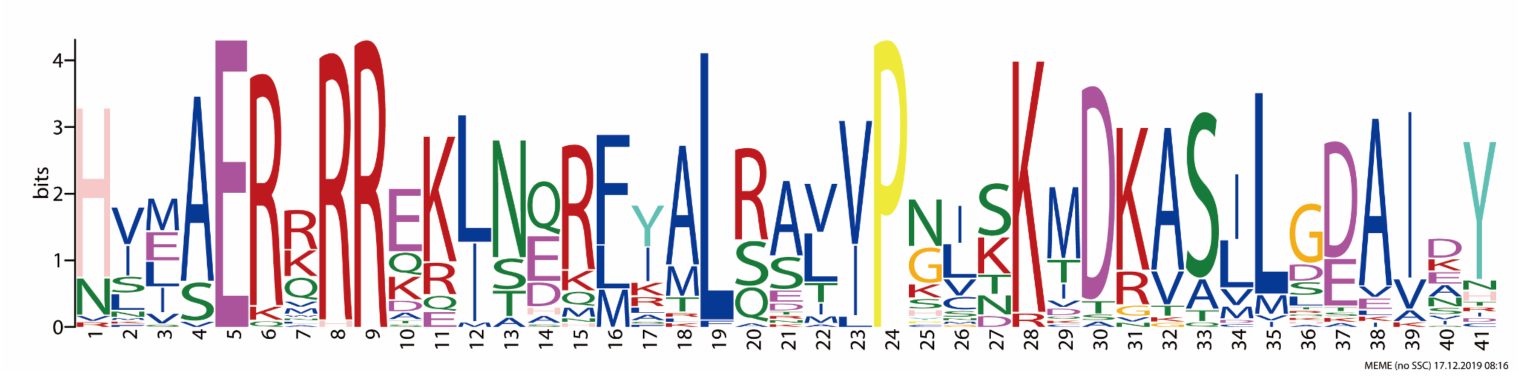

Motif 7

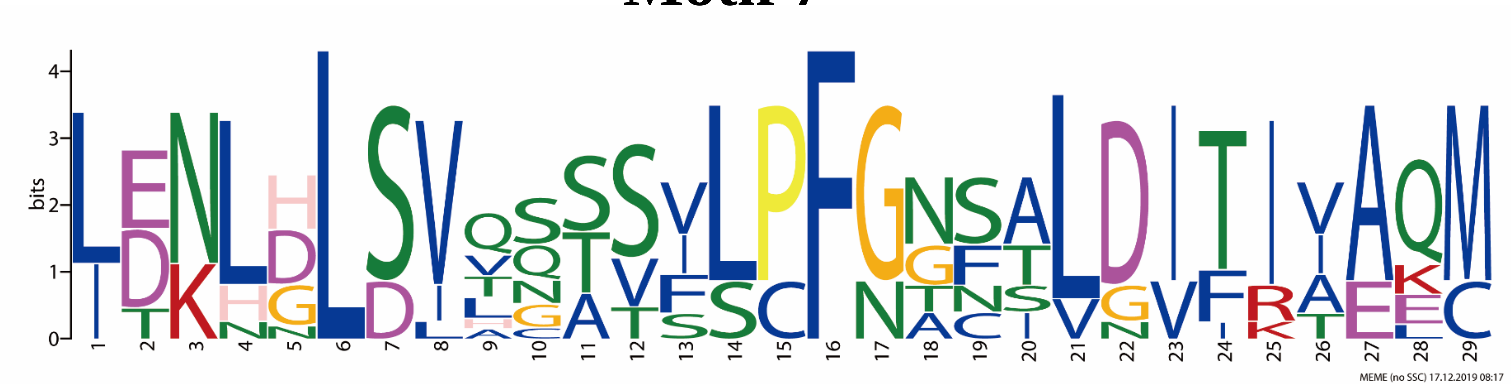

Motif 8

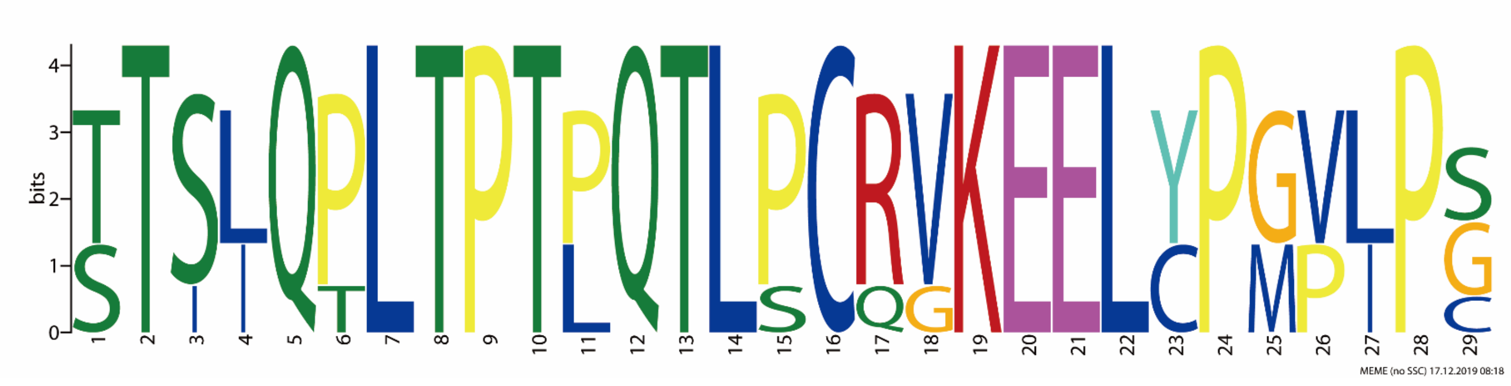

Motif 9

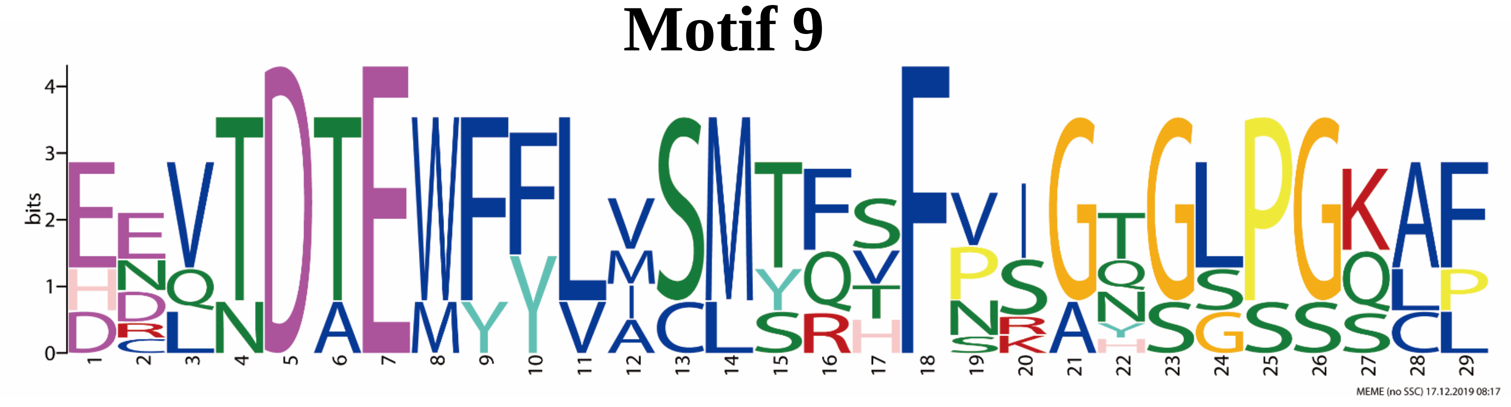

Motif 10

C2H2

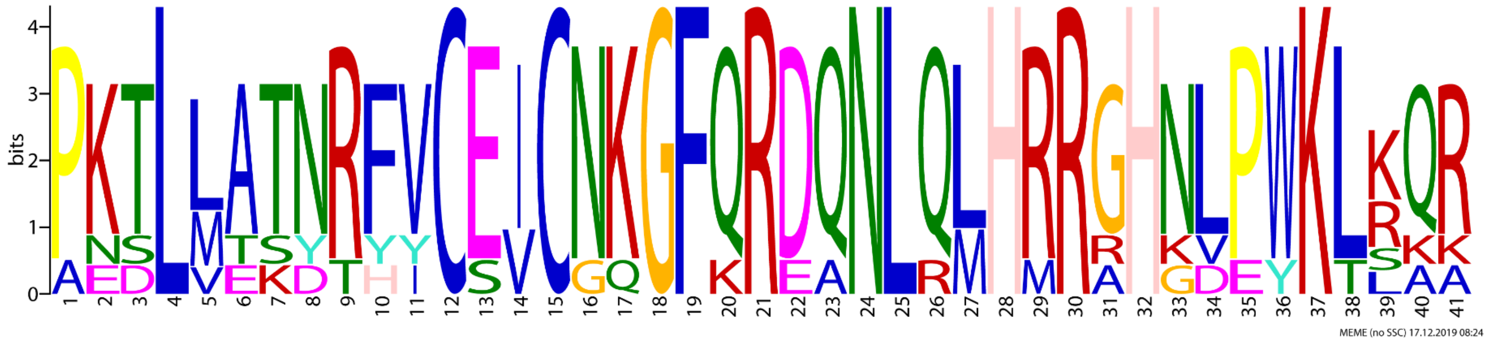

Motif 1

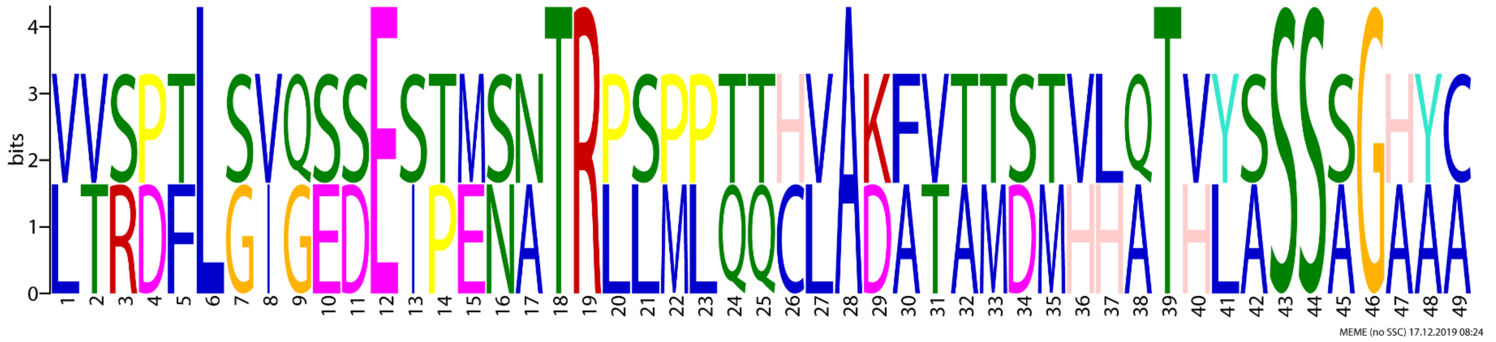

Motif 2

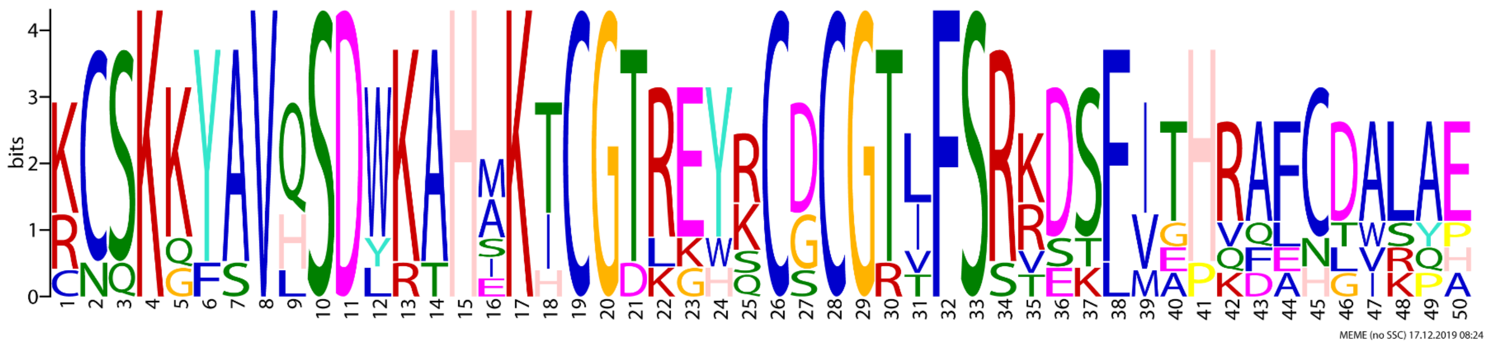

Motif 3

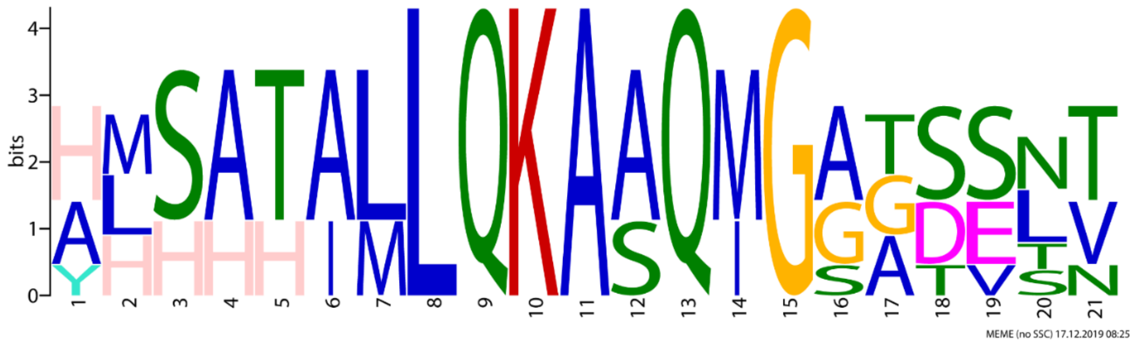

Motif 4

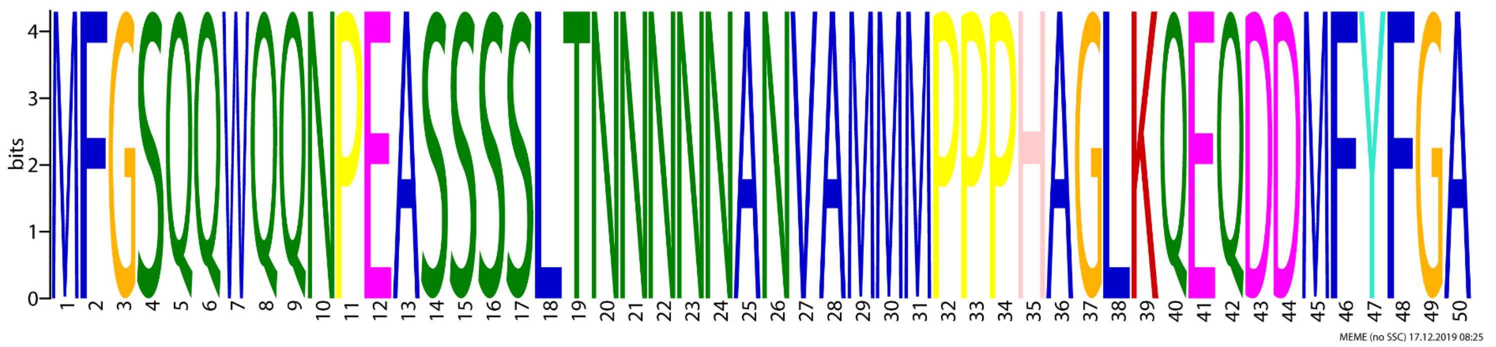

Motif 5

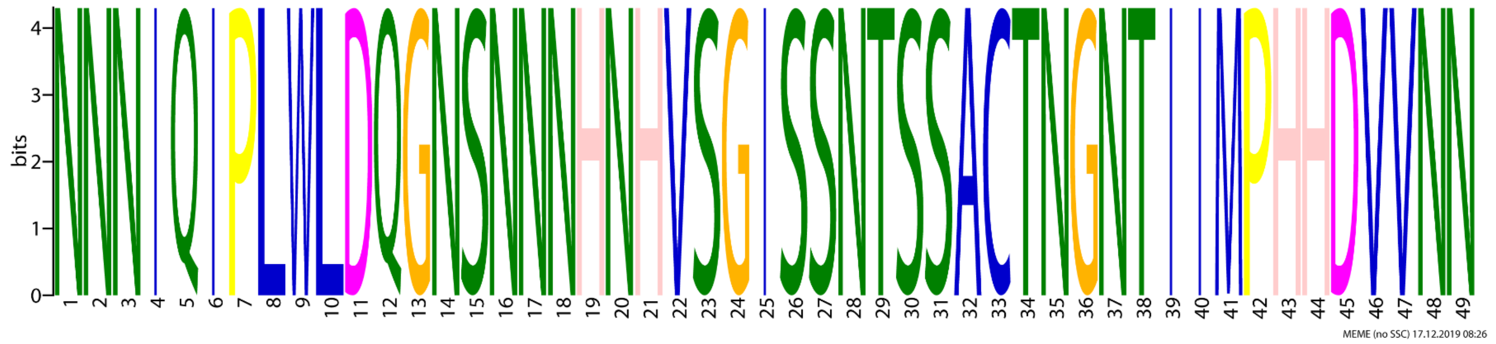

Motif 6

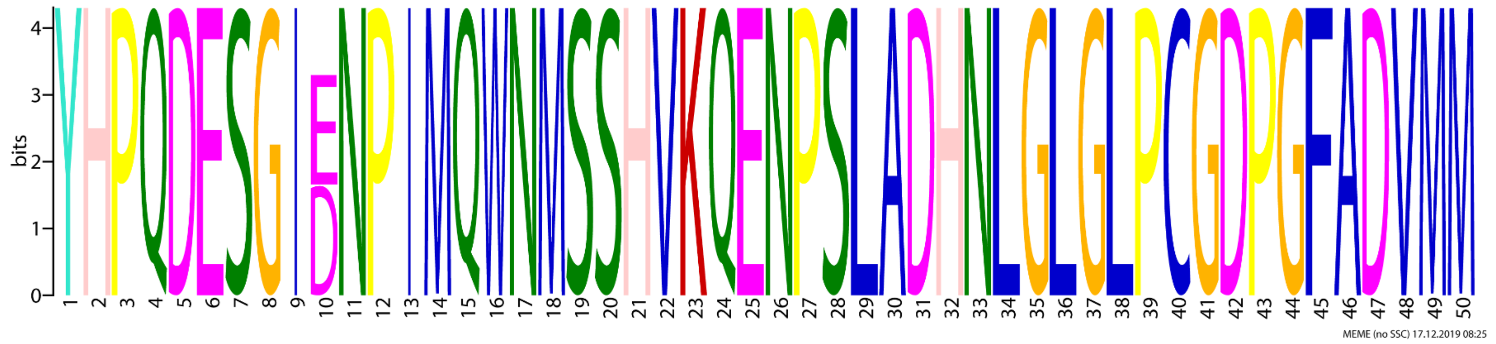

Motif 7

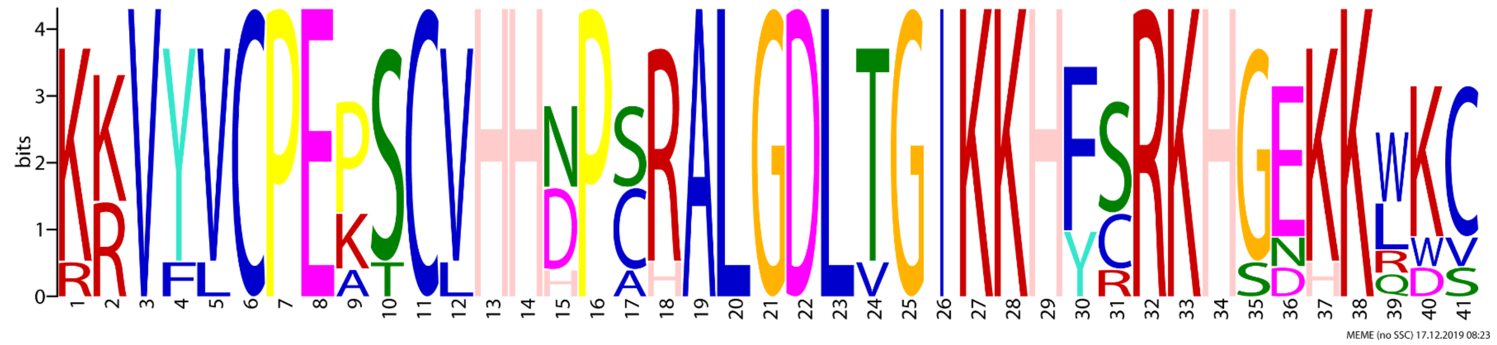

Motif 8

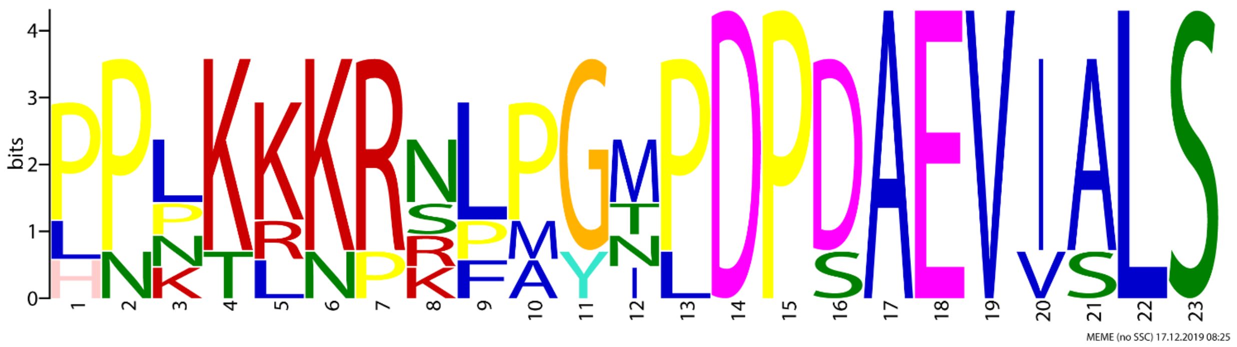

Motif 9

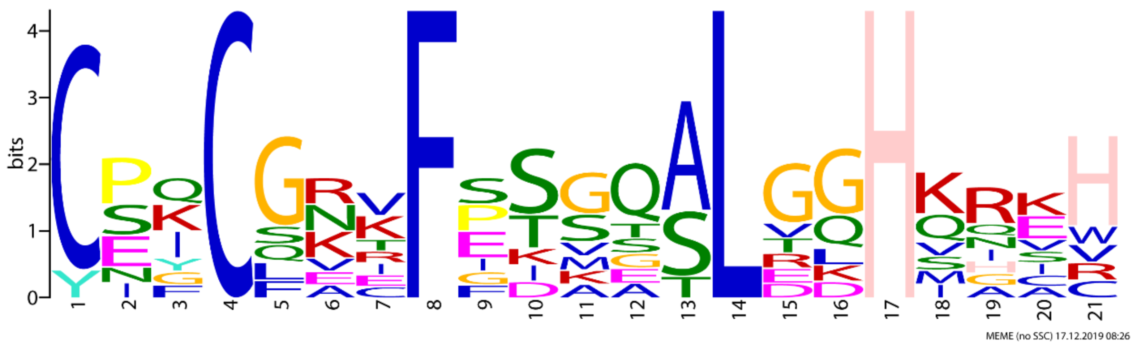

Motif 10

ERF

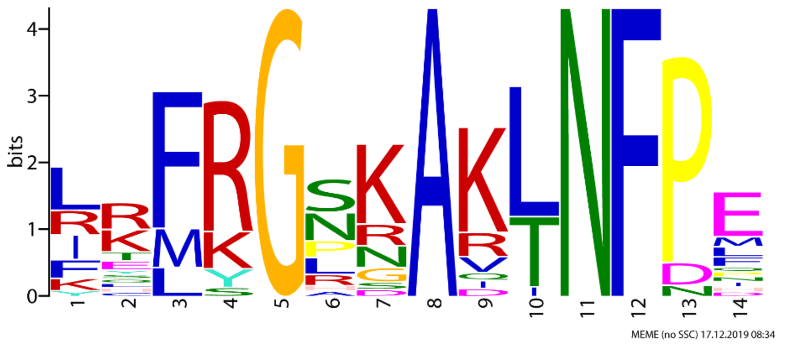

Motif 1

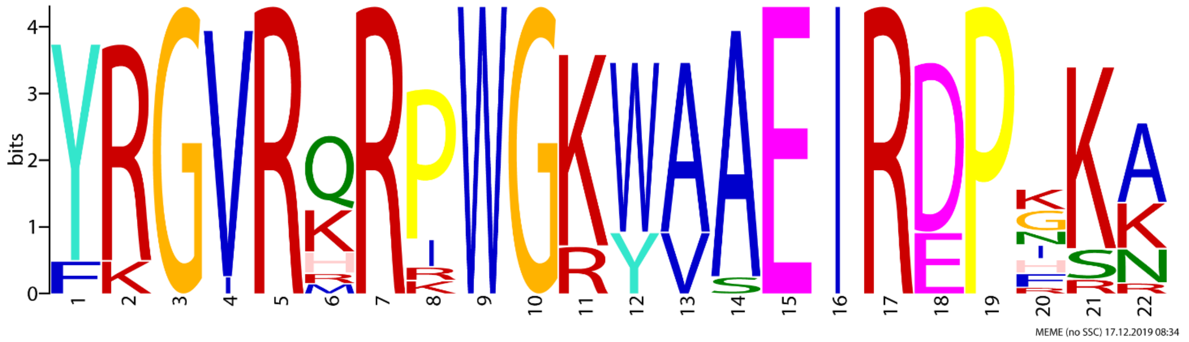

Motif 2

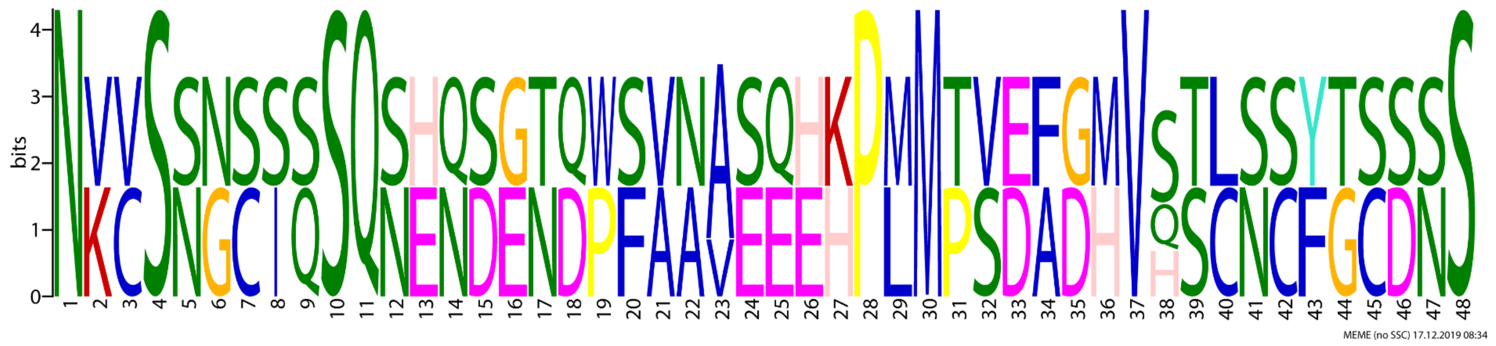

Motif 3

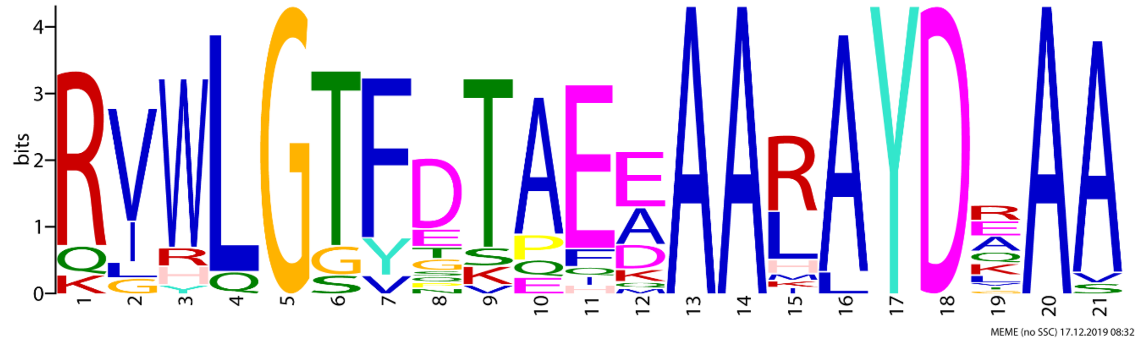

Motif 4

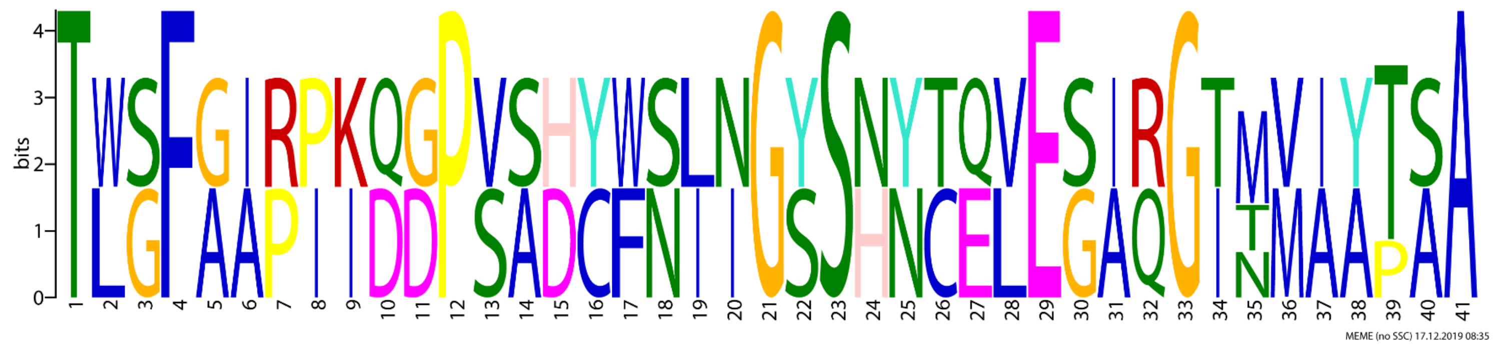

Motif 5

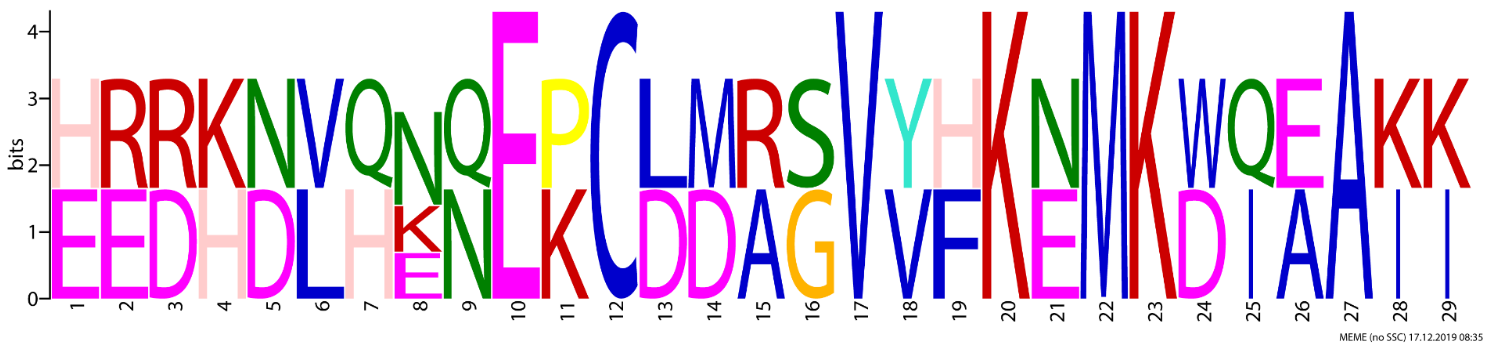

Motif 6

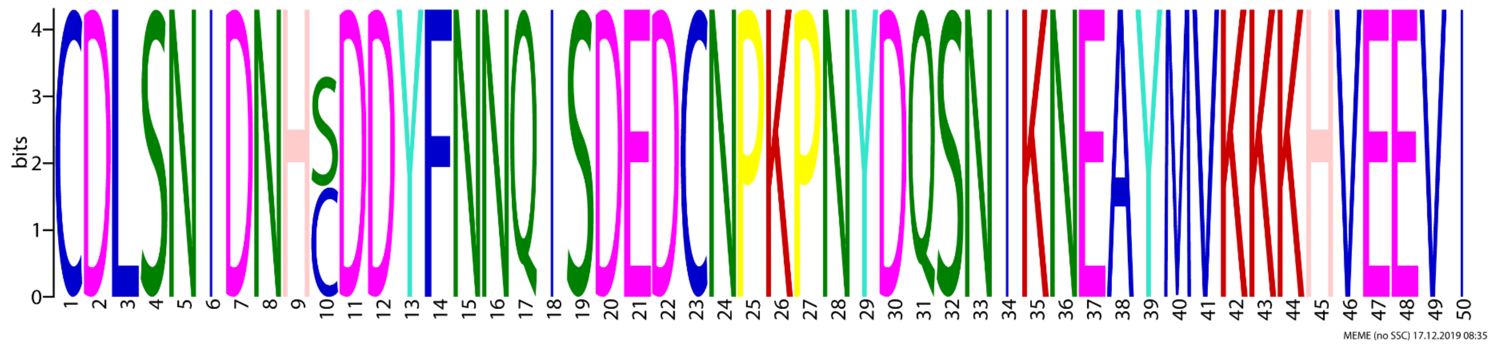

Motif 7

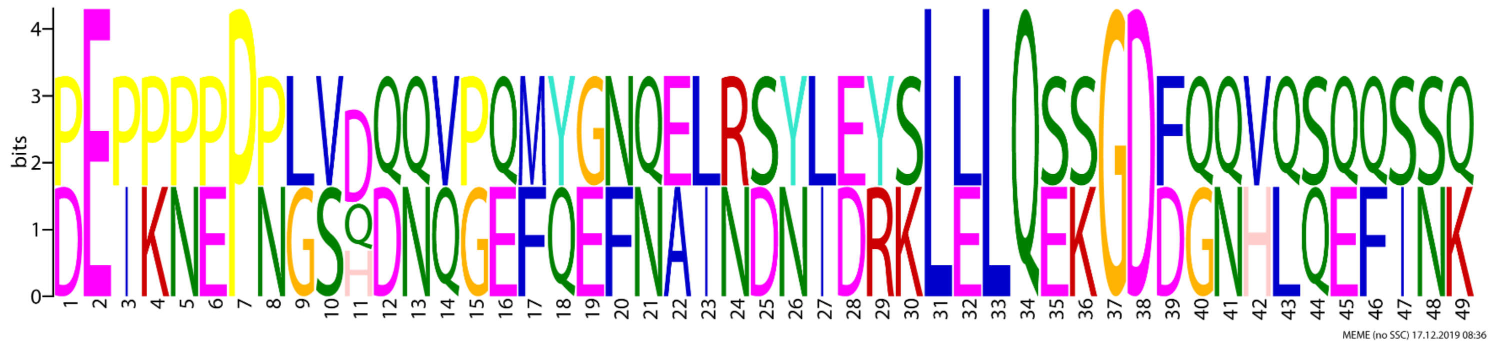

Motif 8

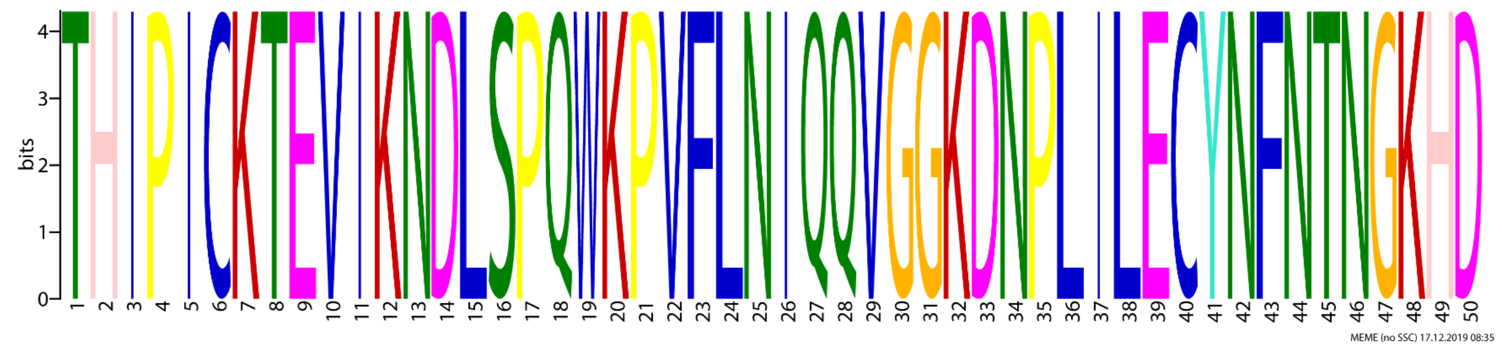

Motif 9

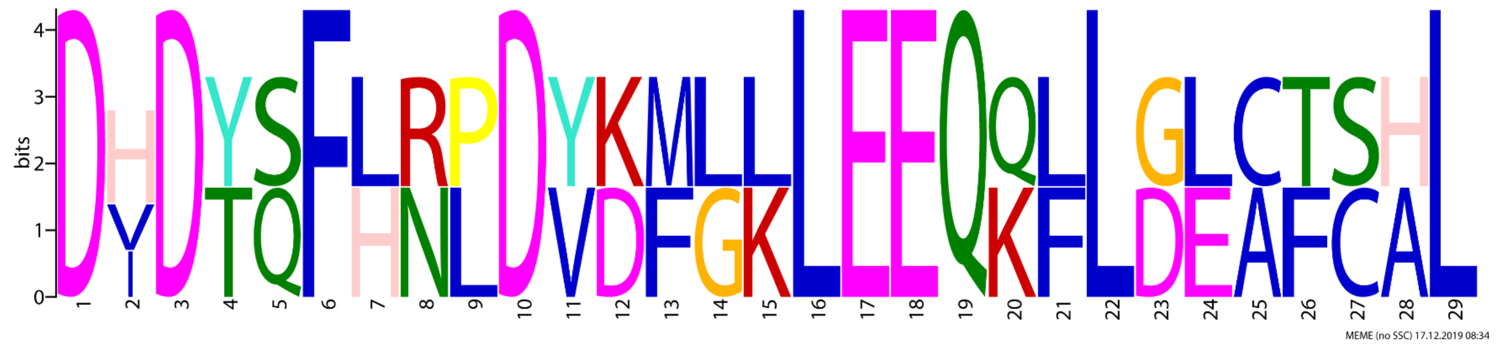

Motif 10

MYB

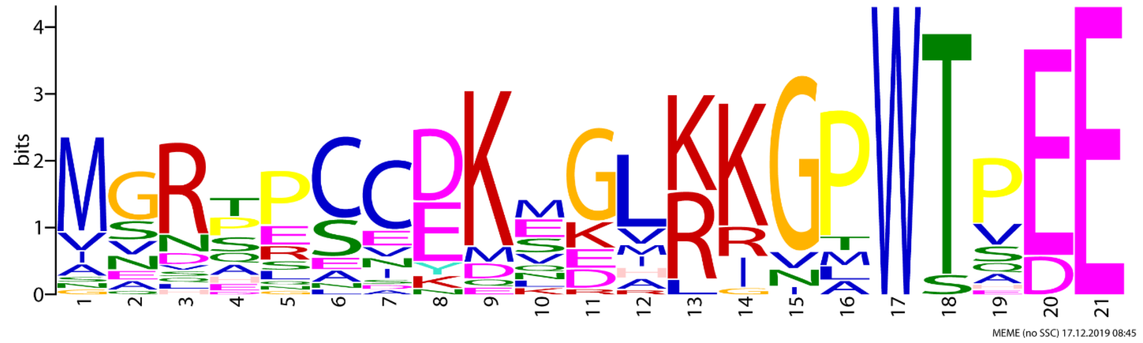

Motif 1

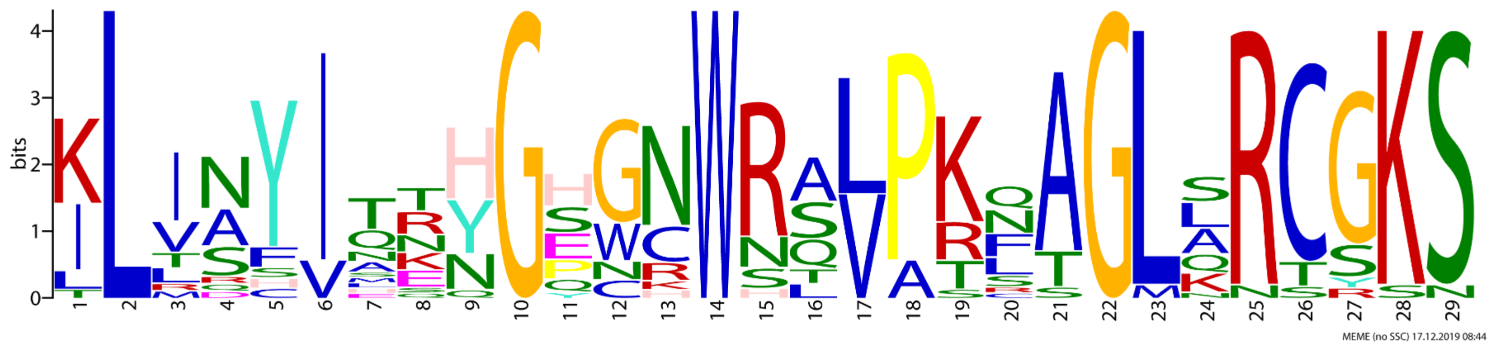

Motif 2

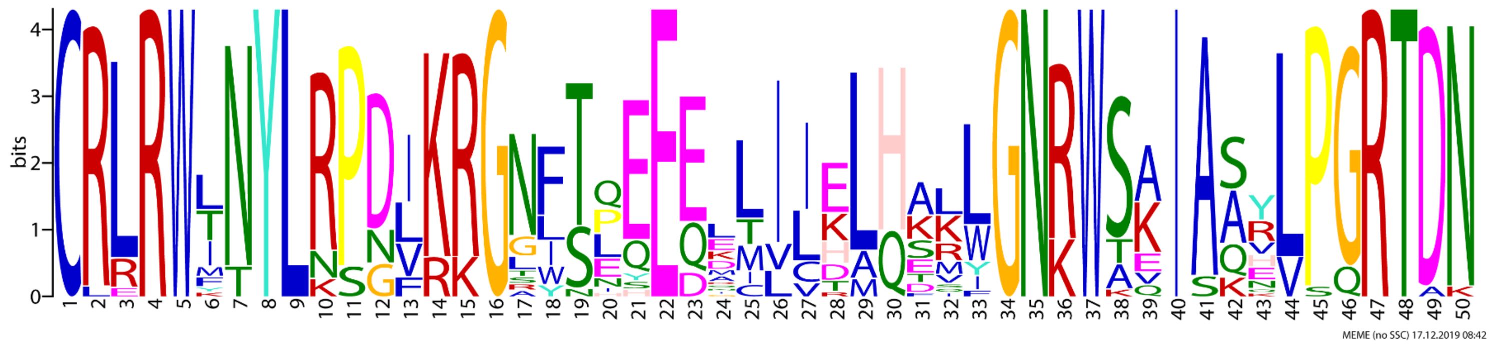

Motif 3

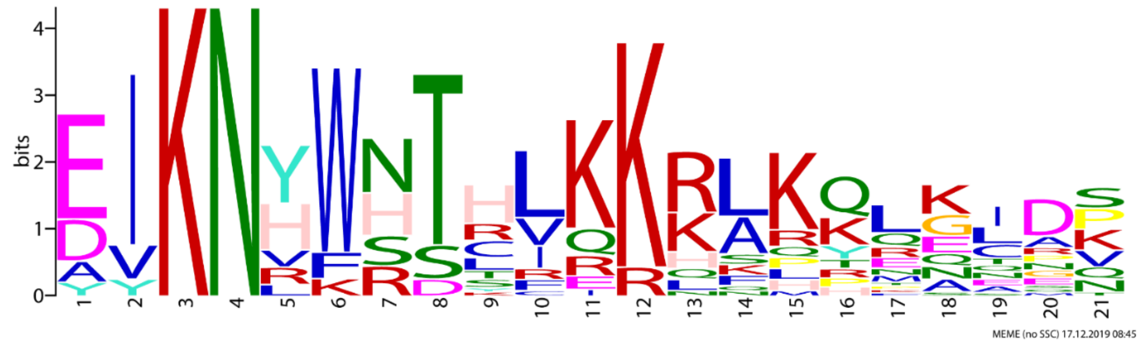

Motif 4

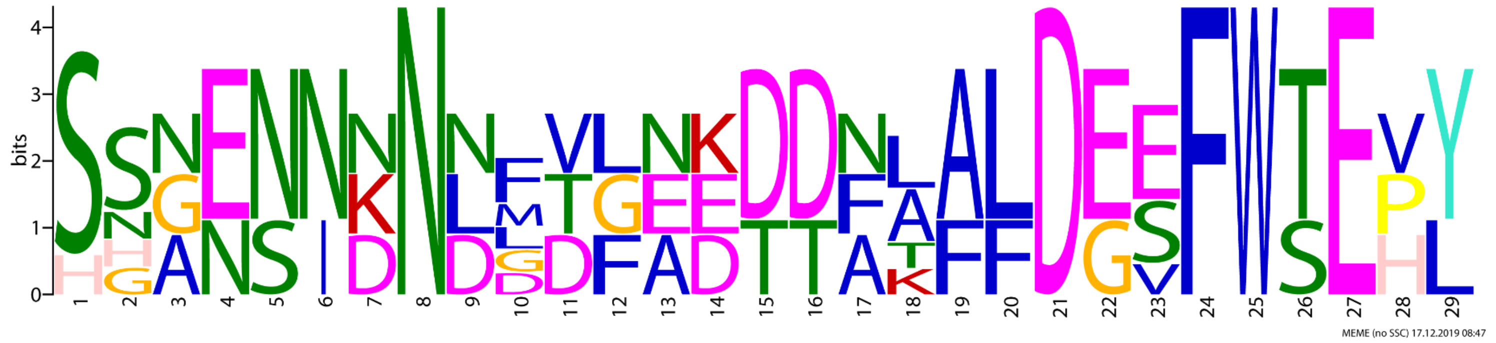

Motif 5

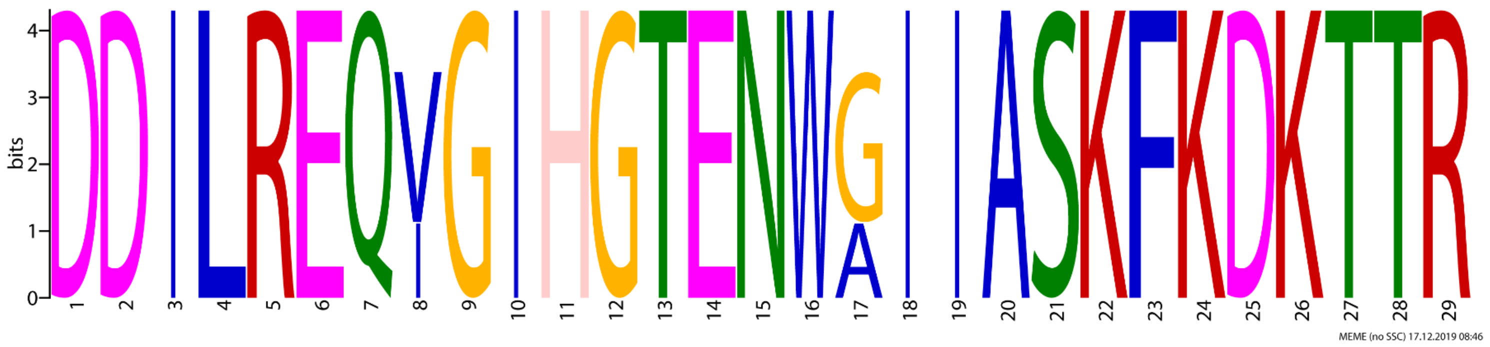

Motif 6

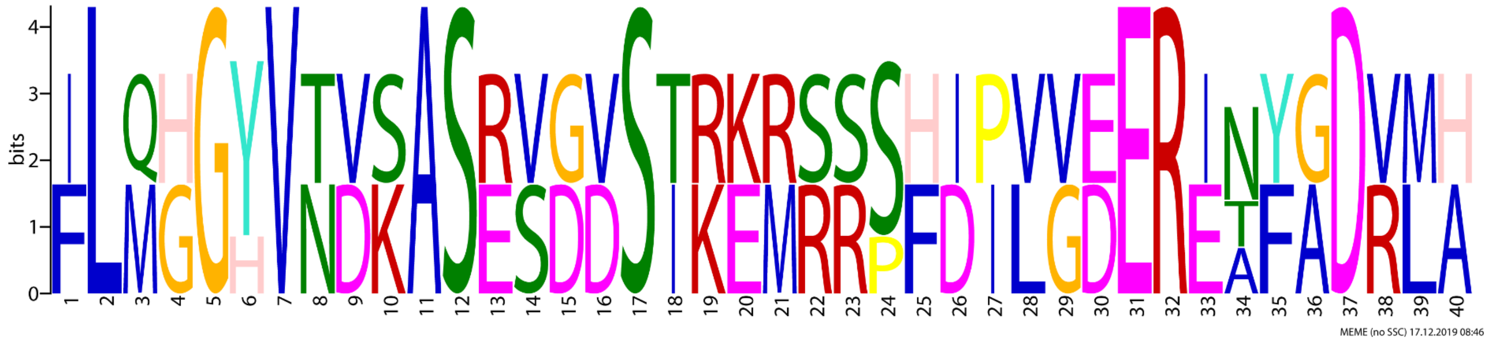

Motif 7

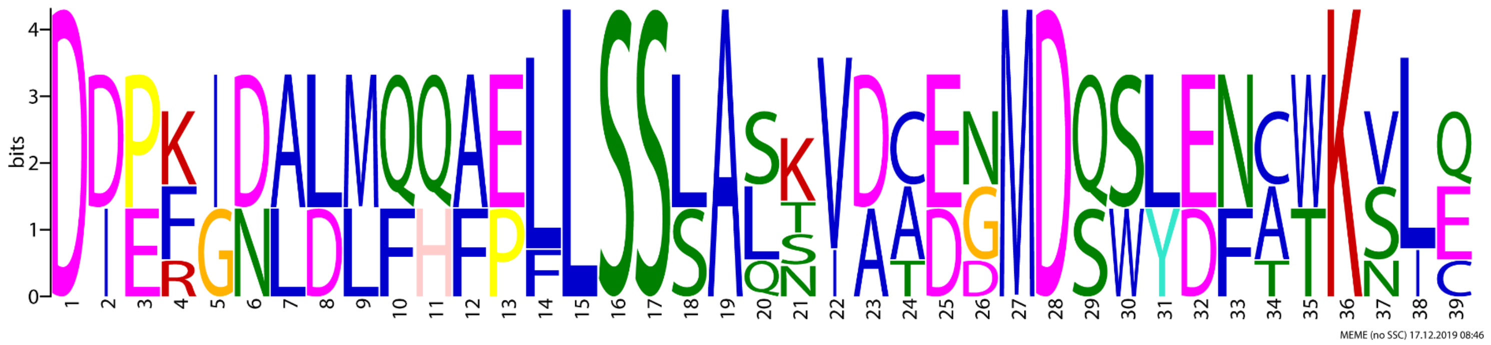

Motif 8

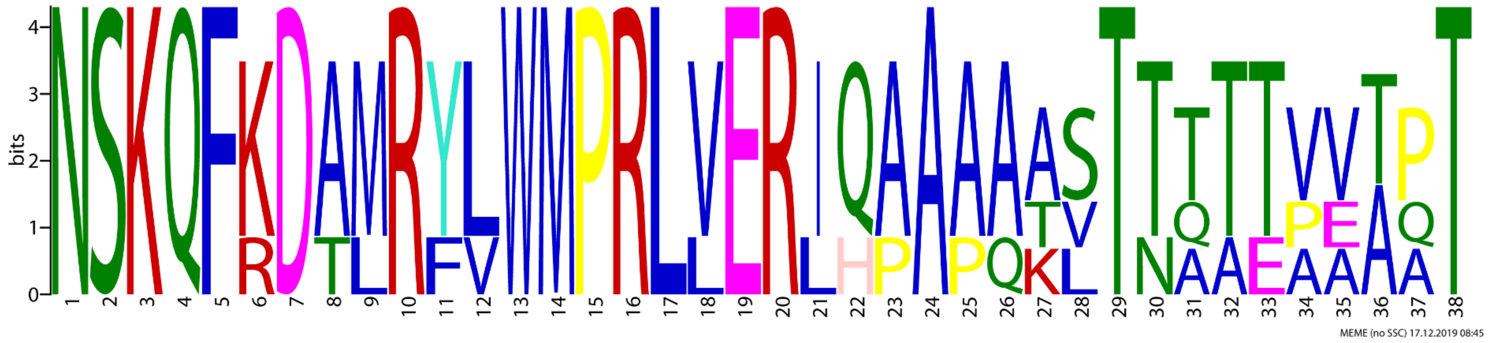

Motif 9

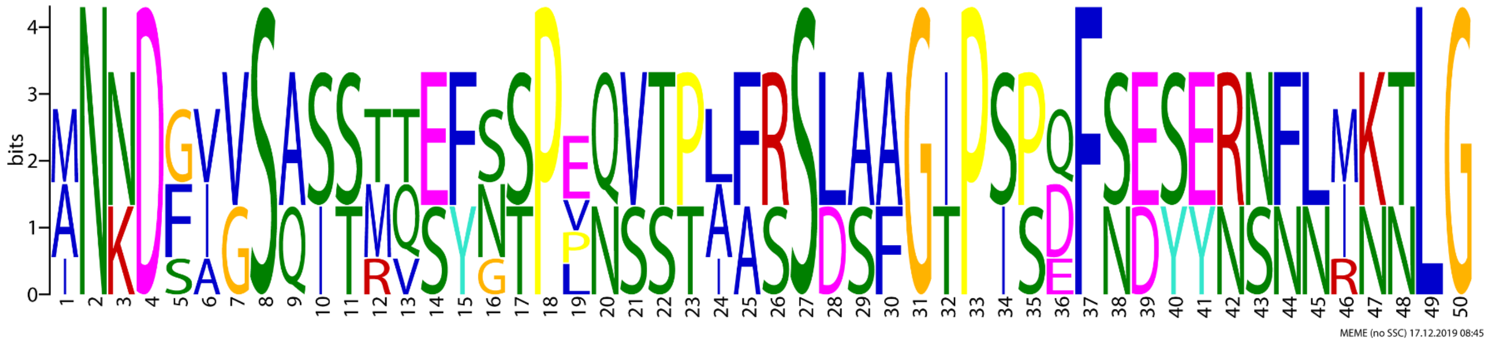

Motif 10

NAC

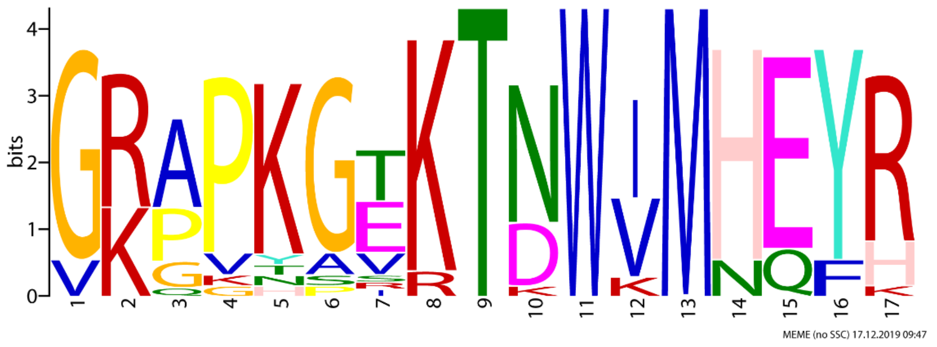

Motif 1

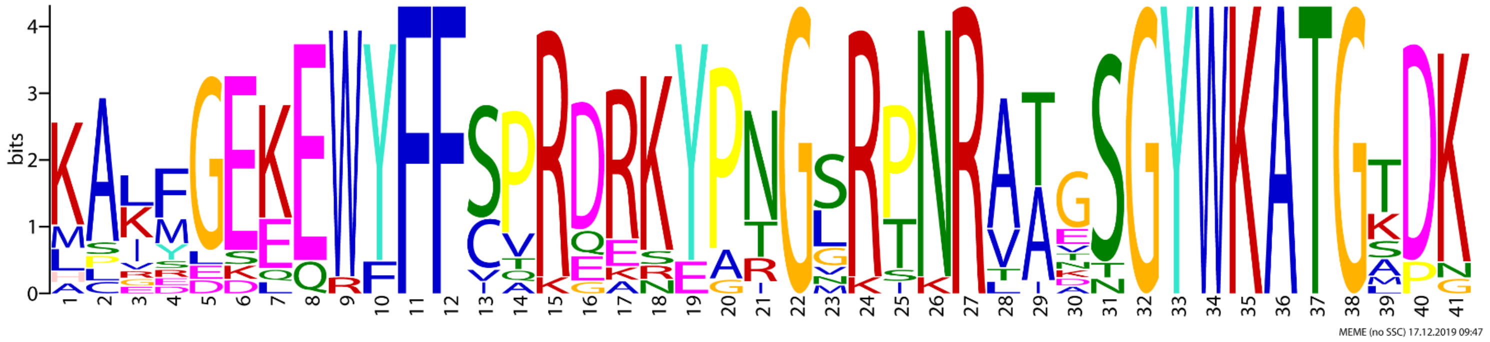

Motif 2

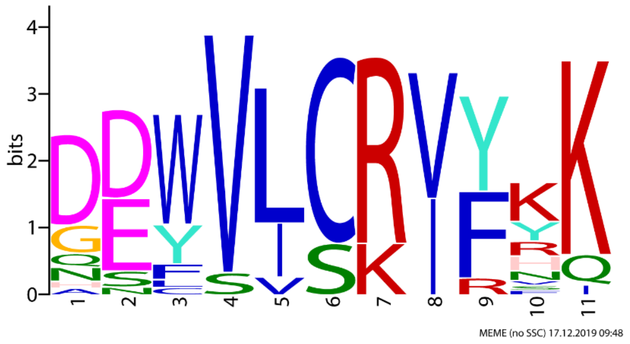

Motif 3

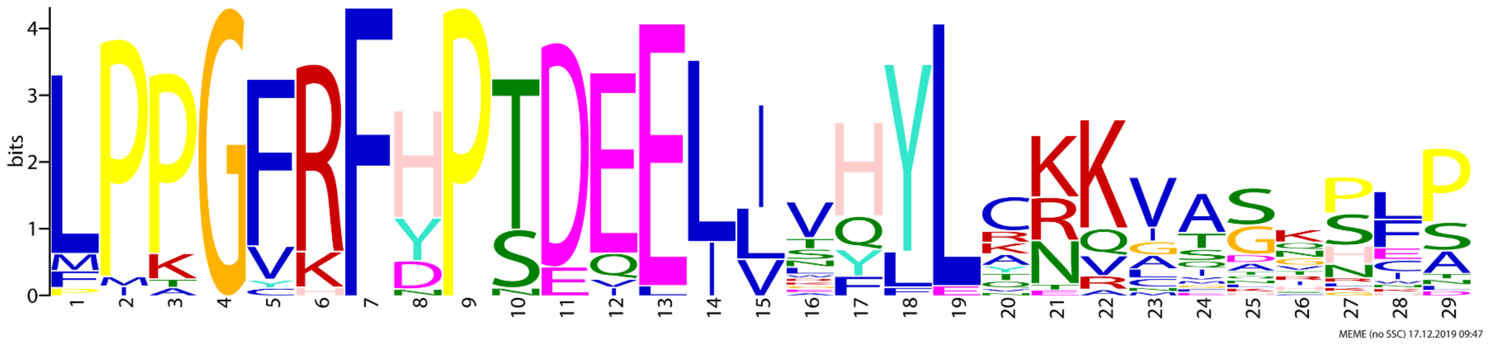

Motif 4

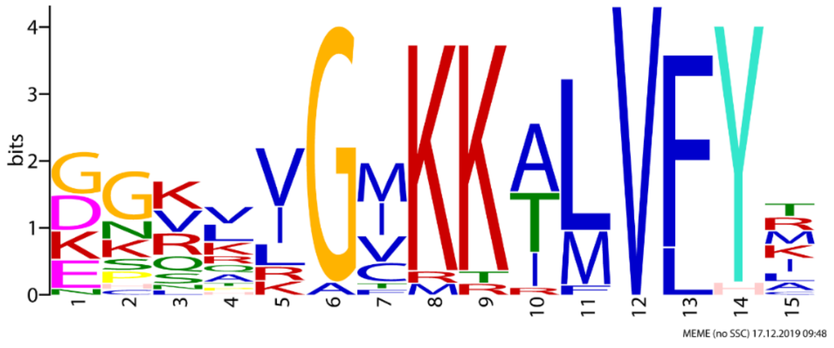

Motif 5

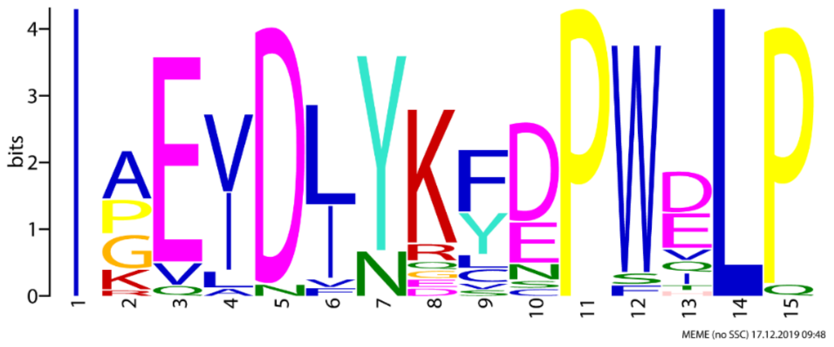

Motif 6

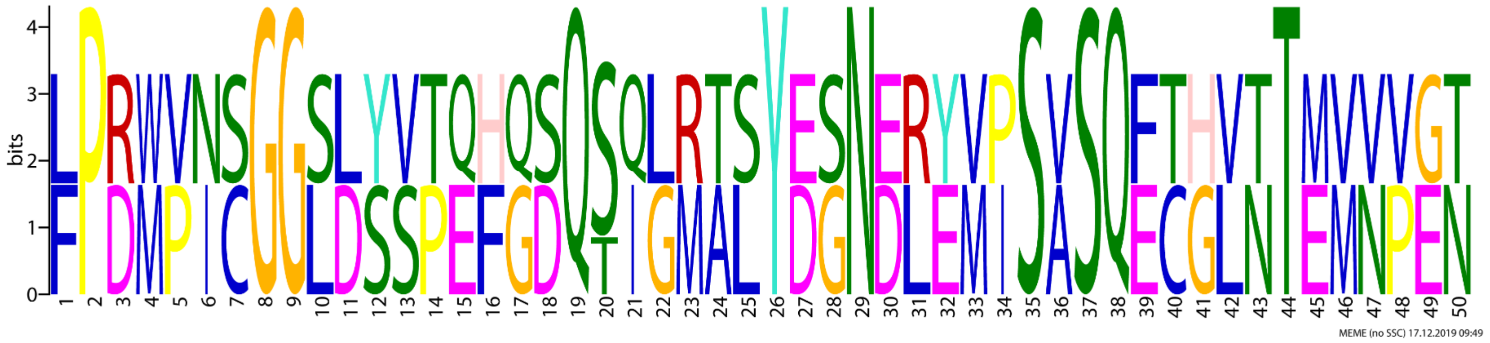

Motif 7

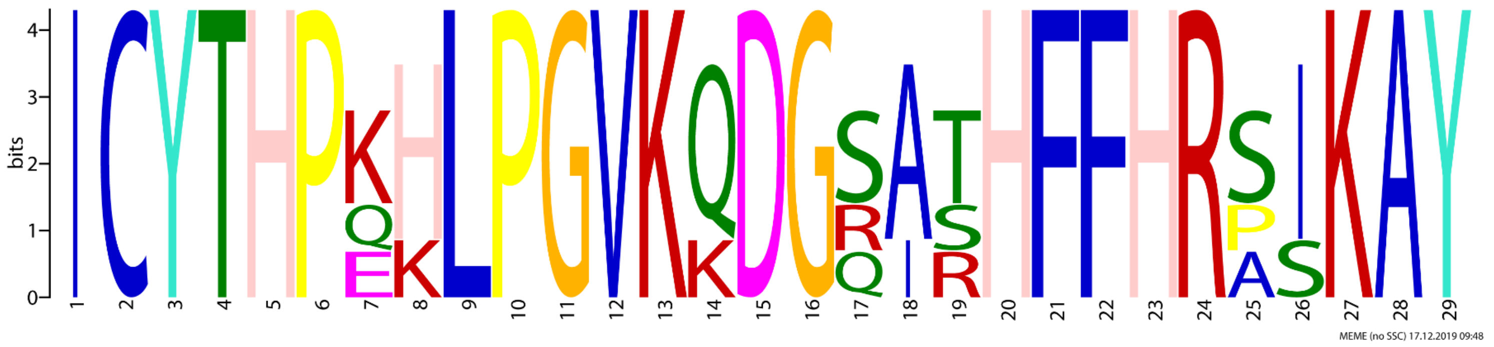

Motif 8

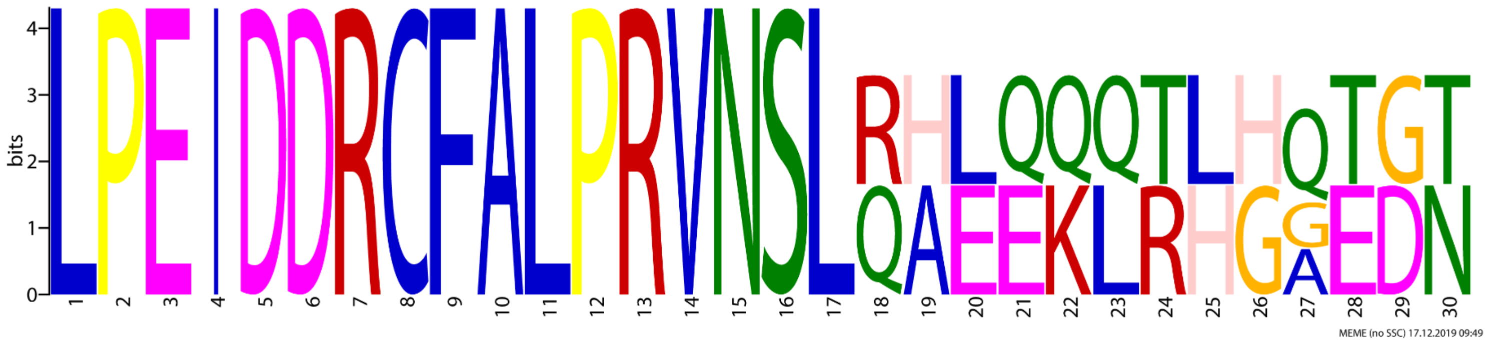

Motif 9

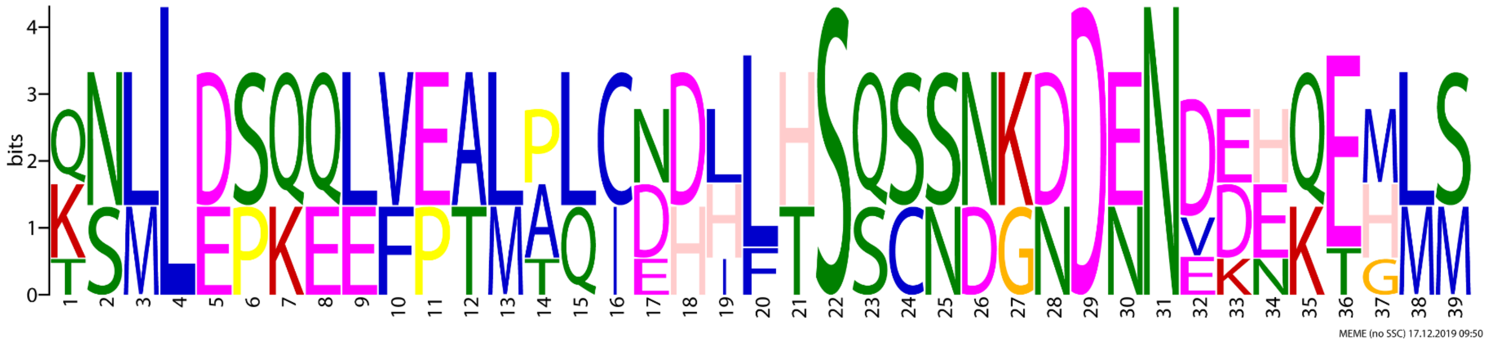

Motif 10

WRKY

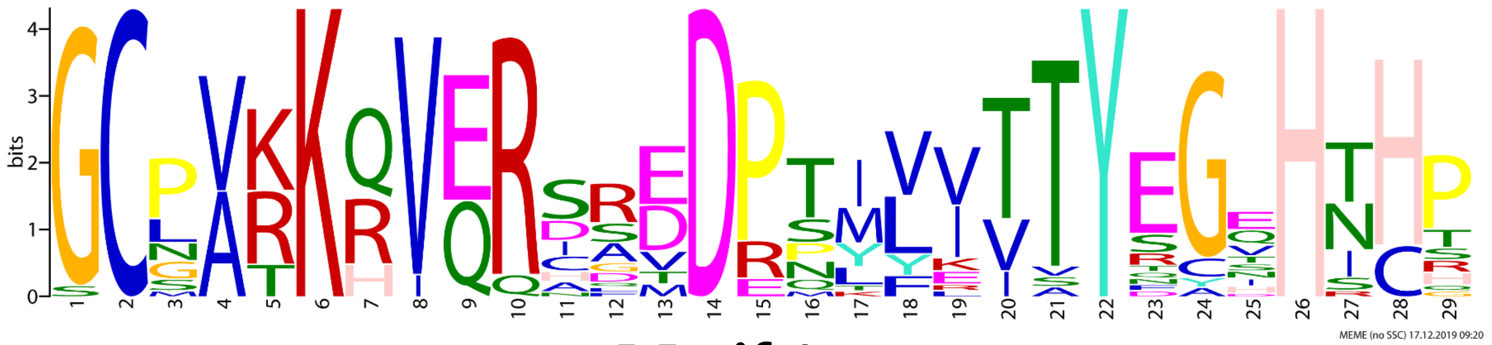

Motif 1

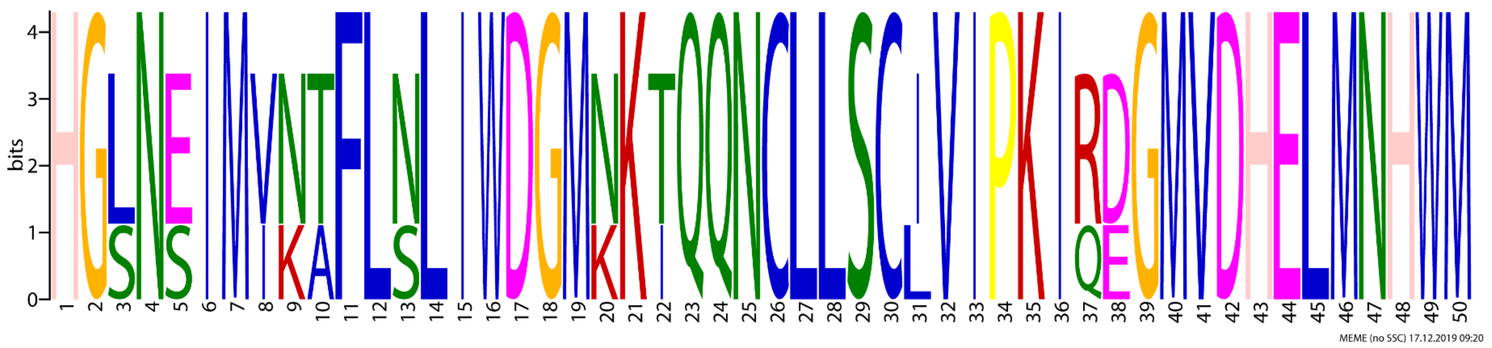

Motif 2

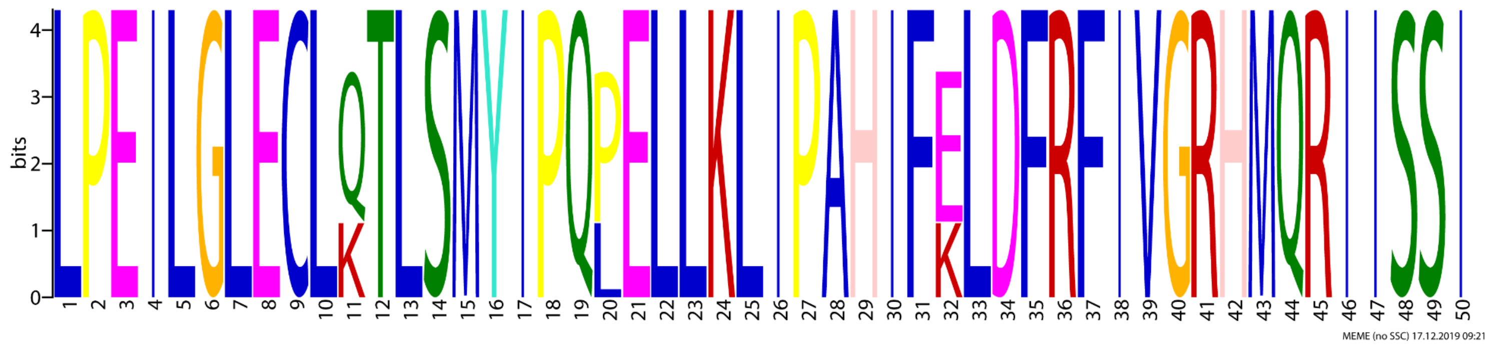

Motif 3

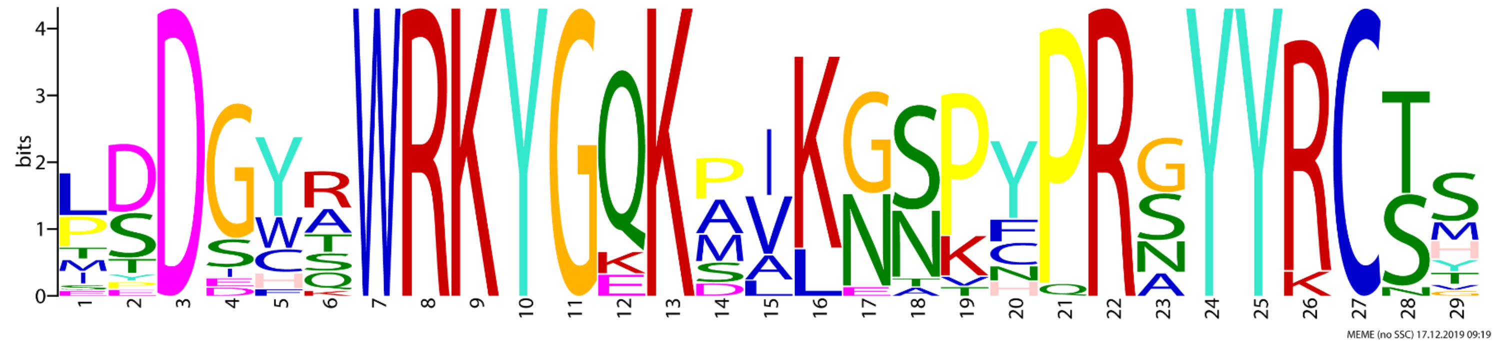

Motif 4

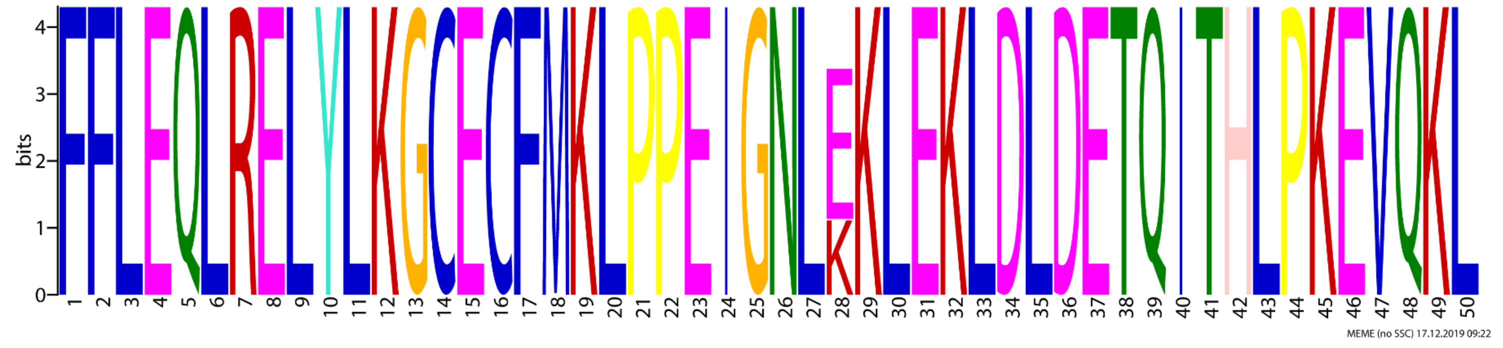

Motif 5

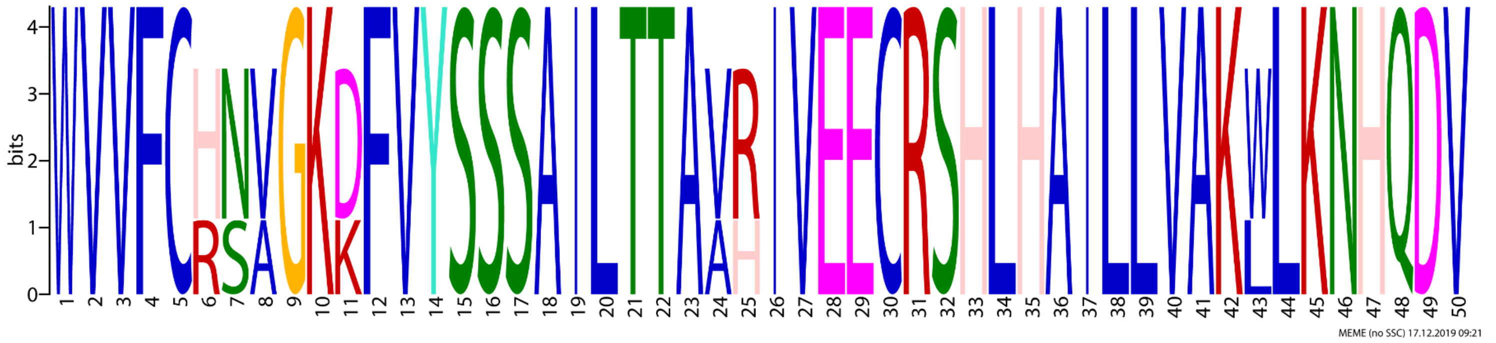

Motif 6

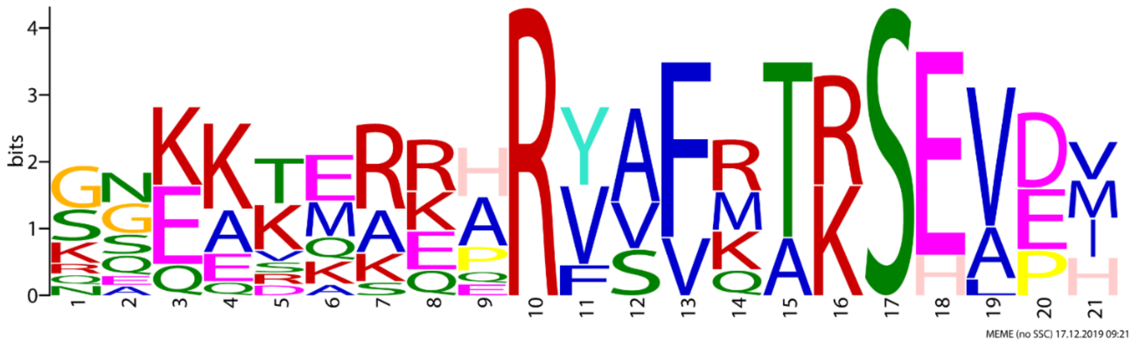

Motif 7

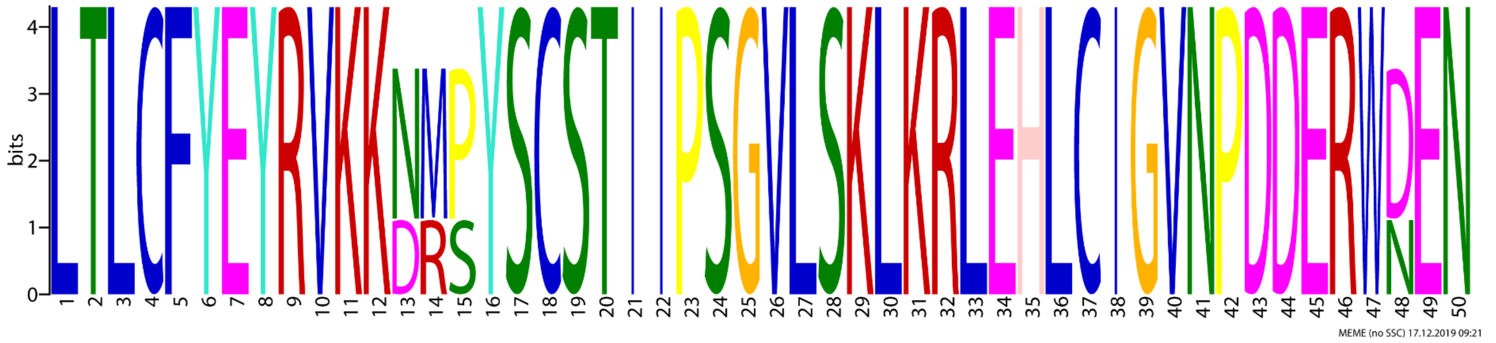

Motif 8

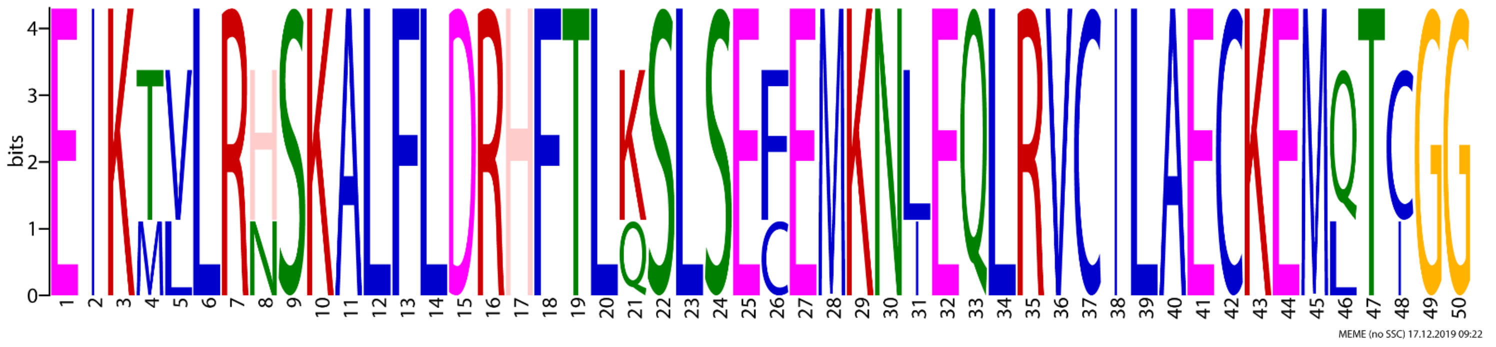

Motif 9

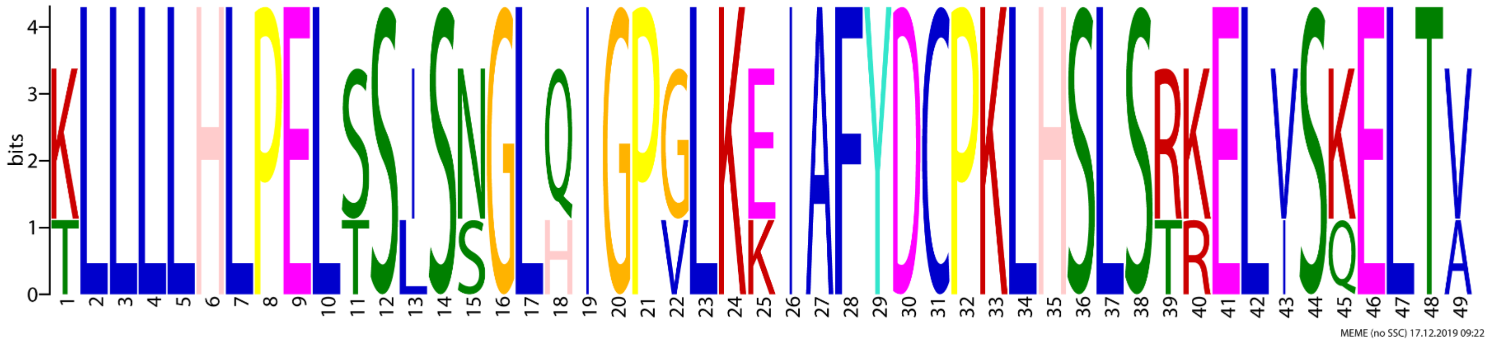

Motif 10
