# Supplementary material for: Comparative Transcriptome-Based Mining and Expression Profiling of Transcription Factors Related to Cold Tolerance in Peanut
Source: Int J Mol Sci. 2020 Mar 11;21(6):1921. doi: 10.3390/ijms21061921 (PMC7139623; doi:10.3390/ijms21061921)
Supplement: Supplementary file 1 [file ijms-21-01921-s001.zip › Supplementary Material/Table S1.docx]

**Table S1** Summary of RNA-Seq reads and their mapping on the peanut genome

| **Samples** | **Total reads** | **Clean reads** | **Clean bases** | **GC content** | **%≥Q30** | **Mapped reads** |
| --- | --- | --- | --- | --- | --- | --- |
| T0-1 | 40,137,800 | 20,068,900 | 5,971,529,812 | 45.40% | 93.56% | 94.21% |
| T0-2 | 39,921,830 | 19,960,915 | 5,955,655,282 | 45.62% | 93.85% | 95.36% |
| T0-3 | 50,730,848 | 25,365,424 | 7,564,675,716 | 46.49% | 93.94% | 91.27% |
| T1-0 | 41,266,812 | 20,633,406 | 6,169,204,568 | 44.13% | 93.34% | 94.61% |
| T1-2 | 40,981,928 | 20,490,964 | 6,126,473,402 | 44.34% | 93.65% | 94.35% |
| T1-3 | 41,575,872 | 20,787,936 | 6,216,244,802 | 44.29% | 93.17% | 94.60% |
| T2-1 | 44,862,806 | 22,431,403 | 6,707,153,856 | 44.75% | 93.46% | 94.66% |
| T2-2 | 41,020,076 | 20,510,038 | 6,120,416,506 | 45.39% | 93.51% | 93.45% |
| T2-3 | 40,685,086 | 20,342,543 | 6,042,441,166 | 45.39% | 93.63% | 89.57% |
| S0-1 | 42,200,230 | 21,100,115 | 6,299,646,916 | 45.38% | 93.50% | 94.66% |
| S0-2 | 44,708,740 | 22,354,370 | 6,670,915,352 | 47.01% | 93.70% | 90.09% |
| S0-3 | 42,006,658 | 21,003,329 | 6,273,938,942 | 45.34% | 93.43% | 94.82% |
| S1-1 | 40,770,860 | 20,385,430 | 6,092,297,710 | 44.34% | 93.41% | 94.33% |
| S1-2 | 43,465,616 | 21,732,808 | 6,493,303,004 | 44.08% | 93.24% | 85.83% |
| S1-3 | 38,946,636 | 19,473,318 | 5,819,343,442 | 44.31% | 93.46% | 94.41% |
| S2-1 | 39,994,358 | 19,997,179 | 5,983,737,418 | 44.26% | 93.22% | 93.23% |
| S2-2 | 46,491,538 | 23,245,769 | 6,938,537,932 | 45.04% | 93.56% | 94.55% |
| S2-3 | 41,156,704 | 20,578,352 | 6,163,111,978 | 44.84% | 93.66% | 94.33% |

*Note.* The 2nd leaves from NH5 (T) and FH18 (S) seedlings after 0-h (T0, S0), 12-h (T1, S1) and 24-h (T2, S2) cold treatments (6°C) was harvested for total RNA extraction, all treatments were repeated three times (-1, -2 and -3). GC content：G and C percentage accounting for the total bases in clean data. Q30: Percentage of the bases whose quality score is greater than or equal to 30.
